# A versatile route to polythiophenes with functional pendant groups using alkyne chemistry

**DOI:** 10.3762/bjoc.12.265

**Published:** 2016-12-09

**Authors:** Xiao Huang, Li Yang, Rikard Emanuelsson, Jonas Bergquist, Maria Strømme, Martin Sjödin, Adolf Gogoll

**Affiliations:** 1 Department of Chemistry - BMC, Uppsala University, Box 576, 751 23 Uppsala, Sweden; 2 Nanotechnology and Functional Materials, Department of Engineering Sciences, Uppsala University, Box 534, 751 21 Uppsala, Sweden

**Keywords:** electropolymerization, functional polymers, polythiophene, Sonogashira coupling, thiophene

## Abstract

A new versatile polythiophene building block, 3-(3,4-ethylenedioxythiophene)prop-1-yne (pyEDOT) (**3**), is prepared from glycidol in four steps in 28% overall yield. pyEDOT features an ethynyl group on its ethylenedioxy bridge, allowing further functionalization by alkyne chemistry. Its usefulness is demonstrated by a series of functionalized polythiophene derivatives that were obtained by pre- and post-electropolymerization transformations, provided by the synthetic ease of the Sonogashira coupling and click chemistry.

## Introduction

Currently organic conjugated polymers are attracting considerable interest for various applications in plastic electronics. In particular, poly(3,4-ethylenedioxythiophene) (PEDOT) [1] and its derivatives [2–7] play an increasingly important role in this field. The attractiveness of PEDOT in organic electronics is due to its electrochemical stability in combination with conductivity and solution processability. Recently demonstrated successful applications include electrochromic materials [8], energy storage materials [2–9], as well as ion sensing devices [10], biosensors [11], and thermoelectric polymers [12]. Therefore, the chemistry of its building block 3,4-ethylenedioxythiophene (EDOT) and the functionalization of the basic structure have been attracting interest as well [13]. The vast amount of research on functionalized polypyrroles [14] and polythiophenes [15] demonstrates that the attachment of functionalized pendant groups to the conjugated polymer backbone provides access to novel properties and applications. An EDOT building block to which highly functionalized pendants can easily be attached thus offers the possibility for rapid access to new functionalized materials.

## Results and Discussion

EDOT functionalization protocols typically involve manipulations of the ethylenedioxy bridge. Thus, the hydroxylmethyl derivative EDOT-CH_2_OH [16], aminomethyl derivative EDOT-CH_2_NH_2_ [17] and the methylenethiol derivative EDOT-CH_2_SH [18], as nucleophiles, as well as the halomethyl derivative EDOT-CH_2_-Cl/Br [6,9] and the exomethylene-EDOT [19] as electrophiles, can be used to form ether, thioether, ester, amine and peptide linkages with the pendant groups. The polar reaction conditions required for their synthesis exclude the use of pendant groups featuring electrophilic or nucleophilic functional moieties, such as esters and alkyl halide, or alcohols and phenols. Furthermore, in the case of EDOT-CH_2_-Cl/Br, the α-hydrogen on the ethylene bridge is acidic and it thus favors β-elimination under basic conditions leading to exomethylene-EDOT [19]. Therefore, the usage of basic nucleophiles is problematic. Moreover, these heteroatom-based linkers between the polymer backbone and the pendant group usually have limited tolerance to acidic or basic conditions promoting hydrolysis, and some of them are electrochemically redox active. The stability of the linker would thus constitute an additional parameter to consider when designing functional polymers.

**Scheme 1:**
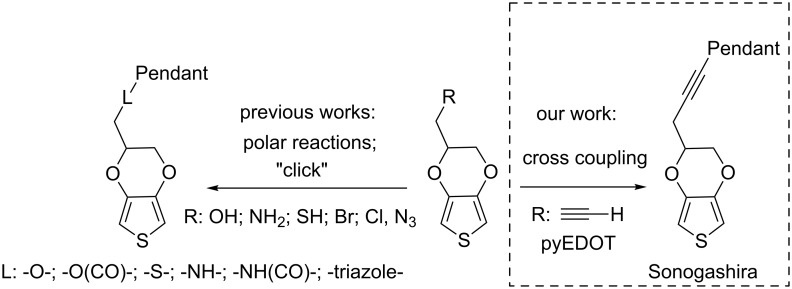
Previous and present EDOT functionalization routes.

The alkyne group is a versatile synthetic building block which can be functionalized in a number of fashions [20–23]. These include, e.g., well-developed cross-coupling reactions, cycloaddition reactions, radical reactions and reductive addition reactions. A terminal alkyne can also react as nucleophile or serve as synthon for pyrrole rings [24–25]. Thus we here fuse the rich alkyne chemistry to the EDOT backbone, resulting in a novel EDOT derivative, the 3-(EDOT)prop-1-yne (pyEDOT, **3**). pyEDOT provides a useful synthon for the synthesis of a variety of EDOT-based polymerizable building blocks. This new EDOT functionalization strategy (Scheme 1), including the polymerization of the resulting building blocks, we illustrate by introducing two examples of such functionalization using a Sonogashira cross-coupling [26] and an azide–alkyne Huisgen cycloaddition [27]. One of the advantages of the cross-coupling and click chemistry is that it allows for reaction conditions tolerant for nearly all of the above mentioned functional groups. Additionally, these functionalizations require only readily available starting materials. A related concept, i.e., attachment of a terminal alkyne moiety to the polymerizable thiophene derivative ProDOT, an EDOT analogue, and its utilization for “click“ chemistry has been reported [28]. However, this involved an ether linkage. Furthermore, substantial differences between ProDOT and EDOT polymers regarding their electrochemical properties have been described [29].

**Scheme 2:**
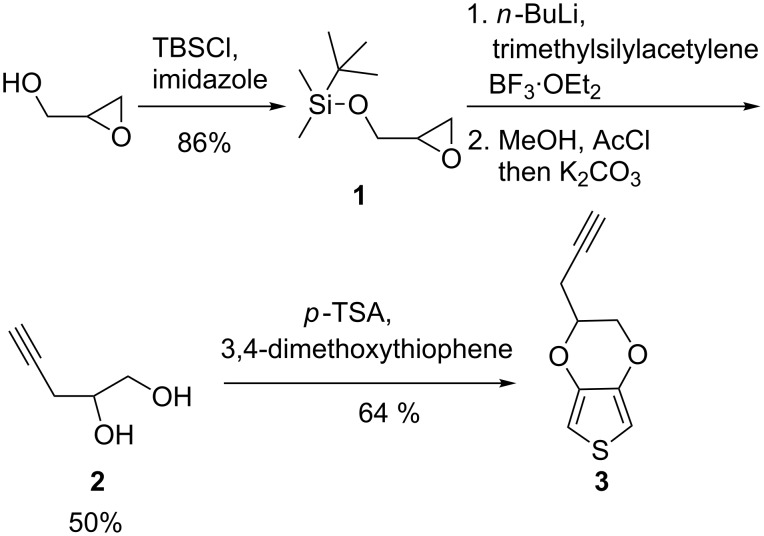
The synthetic route from glycidol to pyEDOT (**3**).

Scheme 2 shows the synthesis of pyEDOT **3**. It starts with glycidol that can be economically prepared by the epoxidation of allyl alcohol [30]. The alkynediol **2** was prepared from glycidol by using a four-step sequence reported by Pattenden and co-workers [31]. The presence of catalytic *p*-toluenesulfonic acid (*p*-TSA) in the toluene solution of 3,4-dimethoxythiophene and **2** resulted in a transetherification reaction to form pyEDOT, which was isolated as light yellow oil by chromatography with a yield of 64%. The yield of the transetherification product was influenced by two factors. Substrate concentrations below 0.5 M resulted in much less polymerization, leaving more diols for transetherification. However, still lower concentrations of less than 0.1 M resulted in lower yields, probably due to decrease of the reaction rate. The yield could also be improved by dividing the addition of the diol **2** into several portions, added over two days. Two or three portions were found to result in the highest yield, with more portions not changing the yield significantly.

**Scheme 3:**
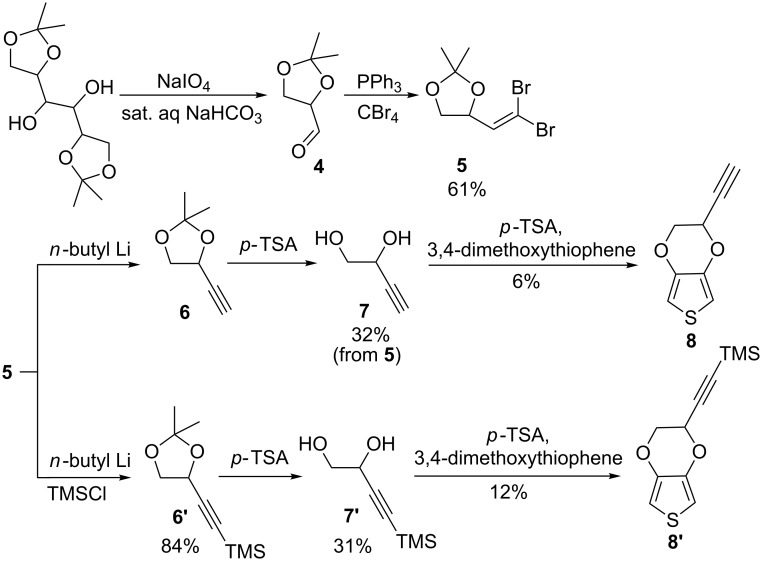
The synthetic route from D-mannitol diketal to eEDOT **8** and TMS-eEDOT **8’**.

Prior to the high yielding pyEDOT synthesis presented here, we attempted to synthesize ethynyl-(EDOT) **8** (eEDOT) and ethynyltrimethylsilane-(EDOT) **8’** (etEDOT) with the alkyne moiety directly attached to the ethylenedioxyl bridge, albeit with much lower yield (Scheme 3). The synthesis started from economically available D-mannitol diketal, 1,2:5,6-bis-O-(1-methylethylidene)-D-mannitol which can be obtained via the hydrogenation of common table sugar [32]. Oxidation of this diketal by NaIO_4_ led to glyceraldehyde [33], which was transformed into the dibromoolefin **5** by Corey–Fuchs reaction. Dibromoolefin **5** was dehalogenated by adding 2 equivalents of *n*-butyllithium to produce 4-ethynyl-2,2-dimethyl-1,3-dioxolane **6**, which was deprotected in acid to give ethynyldiol **7**. However, the transetherification of 3,4-dimethoxythiophene to produce **8** turned out to be difficult, with very low yield of only 6%. Thus, diol **7** was modified by protecting the ethynyl function with a TMS group, yielding **7’**, expecting an improved yield from a better solubility of this diol in toluene. However, the transetherification reaction gave almost no conversion after 2 days. By changing the solvent to dichloroethane a yield of 12% could be obtained, but this would not be sufficient for large scale production, besides considering the environmental impact of requiring a halogenated solvent. Comparing the structural difference between diols **2** and **7**, these results emphasized that the isolation of the alkyne from the ethylenediol by an sp^3^ carbon increased the yield of the transetherification reaction significantly. Attempts were also made to attach an alkyne to EDOT via the reaction between hydroxymethyl EDOT and propargyl tosylate using DABCO as catalyst, but it led to a very low yield and this EDOT-propargyl product was very sensitive to acidic conditions. Considering the robust production of functional alkyne-EDOT and its chemical stability, pyEDOT **3** was thus selected as the precursor of choice for future synthetic and electrochemical studies. pyEDOT can be stored at room temperature over months in an acid-free environment.

**Scheme 4:**
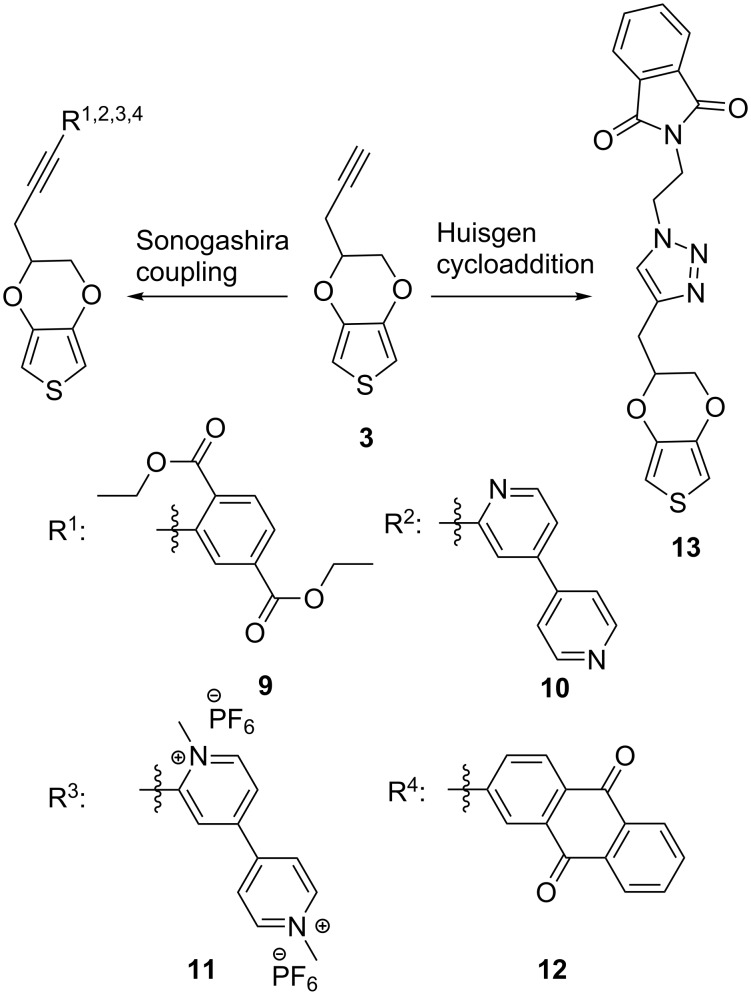
New EDOT derivatives **9–13** accessible from pyEDOT with bromo-pendant group precursors via Sonogashira cross coupling and with an azide pendant group precursor via Huisgen cycloaddition.

The synthetic utility of pyEDOT is demonstrated by the following examples involving a range of pendant groups. The electron acceptor units diethyl terephthalate (DET) and 9,10-anthraquinone (AQ) are of particular interest for their redox chemistry in energy storage applications [34]. Their ester and quinone moieties are vulnerable in nucleophilic and acidic environments. Furthermore, the viologen moiety, available from 4,4’-bipyridine (BP) has potential for energy storage and electrochromic applications [35–36], but it has previously been anchored to a polymer backbone only by oxidative coupling via the nitrogen atom. As shown by our synthesis, all of these electron acceptor units were tolerant to Sonogashira coupling conditions, which produced new EDOT derivatives **9**, **10**, **11** and **12** (Scheme 4). The Sonogashira couplings were performed between the alkyne terminal of pyEDOT and a brominated ring carbon of the pendant group precursors, with yields of 61% (**9**), 82% (**10**) and 65% (**12**), respectively. Methylation of bipyridyl intermediate **10** by methyl iodide produced viologen derivative **11** after exchanging the iodide for the PF_6_^−^ anion. For the methylation step drying of both the reactants **10** and methyl iodide and the solvent was essential, since its omission resulted in polymerization of the viologen-pyEDOT, as indicated by ^1^H NMR (see Supporting Information File 1, Figure S11). We suspect that the polymerization was triggered by the acid generated from methyl iodide reacting with water. Interestingly, the colors of viologen can be tuned by introducing various substituents at the nitrogen sites [37]. The synthesis of **11** illustrates the ease of functionalizing viologen on both nitrogens and it can potentially be used for studying synergic electrochromism coupled with PEDOT [35]. Diethyl 2-bromoterephthalate and 2-bromo-4,4’-bipyridine were prepared according to reported procedures [38–39]. The electron acceptor phthalimide (PT) was attached to EDOT via cycloaddition between the alkyne and an alkylazide to produce derivative **13** (Scheme 4). Regioselective formation of **13** involved the Cu(I)-catalyzed Huisgen 1,3-dipolar cycloaddition between *N*-(2-azidoethyl)phthalimide and the alkyne terminal of pyEDOT under ultrasound conditions in 73% yield. Detailed synthetic routes are presented in Supporting Information File 1.

**Figure 1:**
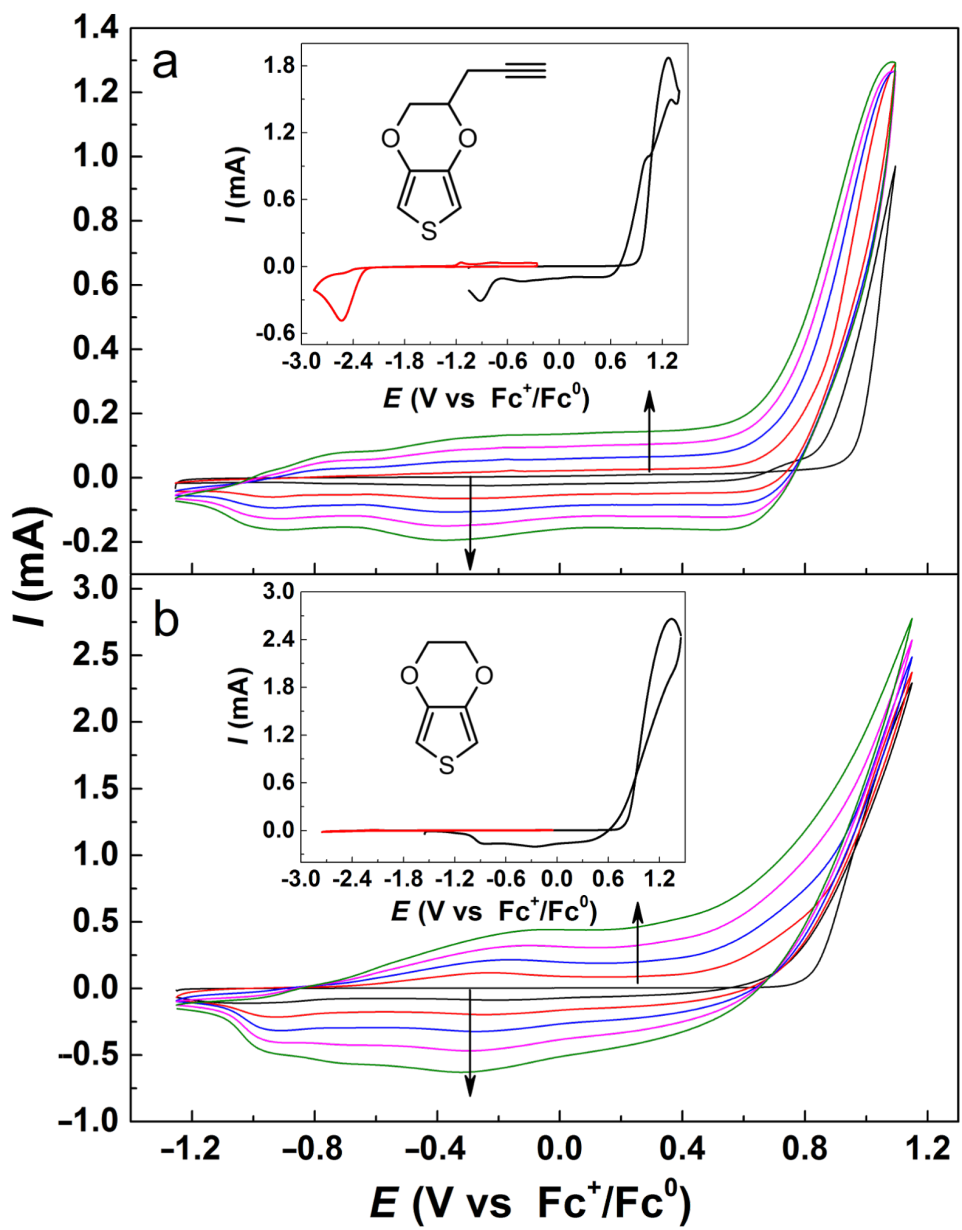
CVs of electrochemical polymerization of (a) pyEDOT **3** and (b) EDOT in MeCN solution with 0.1 M TEAPF_6_, glassy carbon electrode, 0.1 V s^−1^. Insets show the structure and voltammograms for the monomers.

Figure 1a shows the cyclic voltammograms (CVs) for electrochemical polymerization of pyEDOT on a glassy carbon working electrode. For comparison, the CV for electrochemical polymerization of pristine EDOT under the same conditions is shown in Figure 1b. Similar voltammetric responses were obtained for the polymerization of pyEDOT and EDOT, with increased current in the potential region from −1.2 to 0.6 V vs ferrocene/ferrocenium (Fc^+^/Fc^0^) during polymerization cycling. Apparently, the presence of an ethynyl group on pyEDOT did not disturb the polymerization of the EDOT backbone. The CVs of pyEDOT indicated an irreversible reduction reaction at a potential lower than −2.4 V vs Fc^+^/Fc^0^ as shown in the inset of Figure 1a. This is believed to indicate the reduction of the triple bond. The ease of pyEDOT polymerization should also allow for further post-polymerization functionalization [40].

**Figure 2:**
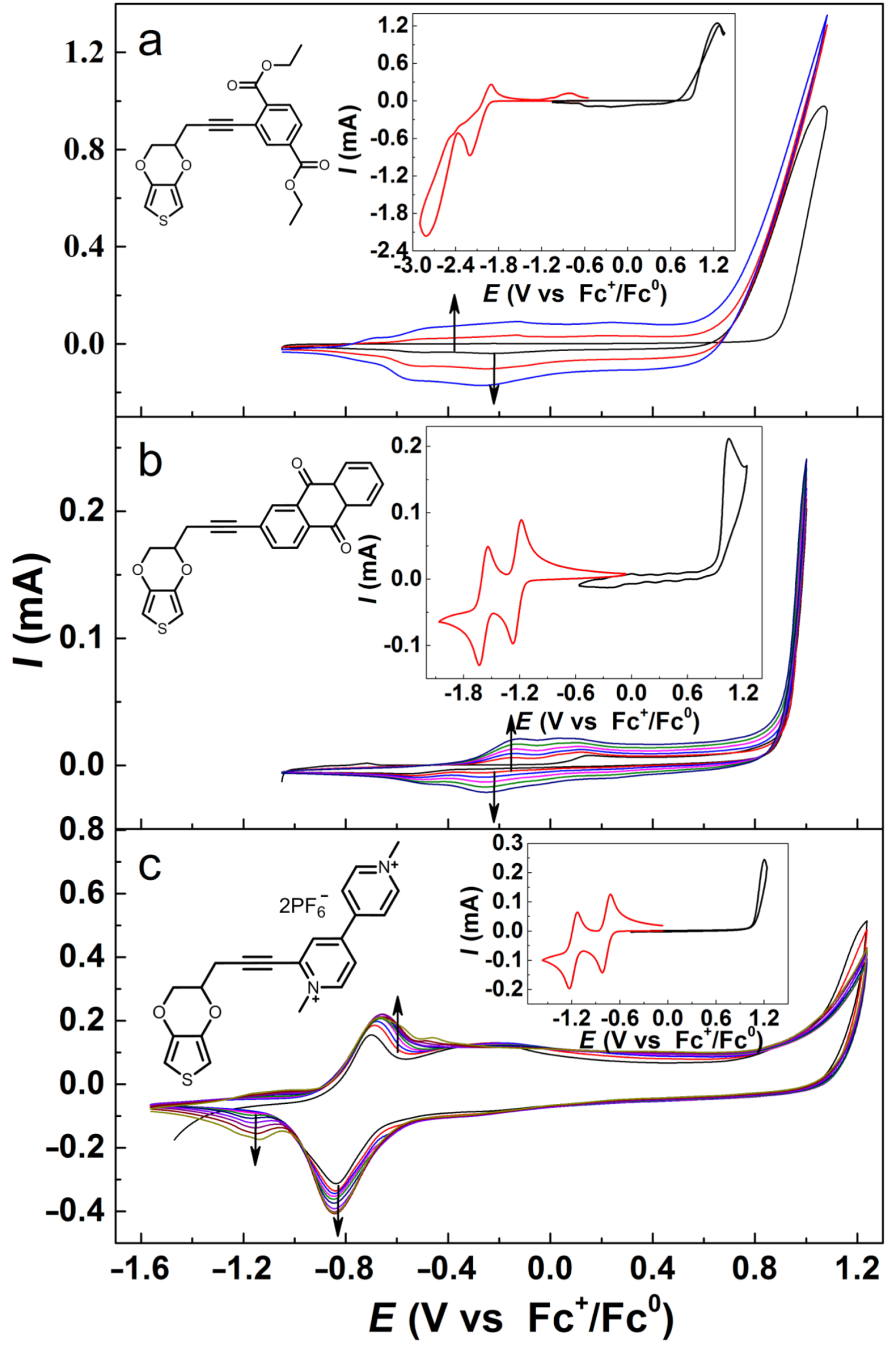
CVs of electrochemical polymerization of (a) pyEDOT-DeT (**9**), (b) pyEDOT-AQ (**12**) and (c) pyEDOT-MVPF6 (**11**) in MeCN with 0.1 M TEAPF_6_, GC, 0.1 V s^−1^. Insets show the structure and voltammograms for the monomers. Polymerization of pyEDOT-MVPF_6_ was performed on a PEDOT-modified GC electrode.

Figure 2 presents the CVs of the pyEDOT derivative monomers and their polymerization in MeCN solution. All compounds showed the electrochemical activities of both the pendant group and the EDOT moiety individually, giving redox reactions of the pendant groups in a negative potential region (the red curves in the insets of Figure 2) and oxidation of the EDOT unit in a more positive potential region (the black curves in the insets of Figure 2). Comparable voltammetric behaviors for the polymerization of pyEDOT-DeT (**9**) and pyEDOT-AQ (**12**) were obtained, similar to those of pyEDOT and EDOT as presented in Figure 1. For pyEDOT-MVPF_6_ (**11**), the polymerization was performed in a potential region covering both EDOT-based oxidation and MV-centered reductions. The build-up of the MV-centered redox peaks is clearly evident at −0.8 V vs Fc^+^/Fc^0^ and −1.2 V vs Fc^+^/Fc^0^ corresponding to the MV^2+/+^ redox reaction and the MV^+/0^ redox reaction, respectively. The CVs of these polymers can be found in Supporting Information File 1, Figure S39, and the polymer properties will be characterized further in a separate study. The successful polymerization of these functionalized pyEDOT monomers exemplifies the capabilities that pyEDOT derivatives can bring for the synthesis of new polymers and property studies.

The phthalimide-EDOT derivative **13** failed to polymerize. A plausible reason could be an interference of the electron rich aromatic moieties with the thiophene radical cations formed during polymerization, in analogy to previous suggestions from Bäuerle et al. for triazolomethyl-substituted EDOT. Post-functionalization of the azido-PEDOT polymer by click chemistry turned out to be a solution to this problem [41]. To confirm the viability of this alternative, in a similar fashion, post-functionalization of the poly(pyEDOT) was performed in an acetonitrile solution of phthalimide-azide in the presence of catalytic amounts of Cu^+^(CH_3_CN)_4_PF_6_^−^ and elemental copper. The reaction to correspondingly functionalized PEDOT was stopped after three days at room temperature. After washing the “click”–functionalized electrode it was characterized electrochemically. The redox peaks appearing at −2.0 to −1.5 V vs (Fc^+^/Fc^0^) indicated the attachment of phthalimide to the PEDOT backbone (Supporting Information File 1, Figure S40).

## Conclusion

In conclusion, we have introduced a new functional pyEDOT featuring a terminal alkyne which endows EDOT or PEDOT functionalization with the rich chemistry of alkynes. We exemplify this application of a C–C bond forming synthesis for the PEDOT functionalization by a Sonogashira coupling yielding the first terephthalate functionalized EDOT monomer as well as a viologen unit with symmetrical N-substitution attached to an EDOT core. These new monomers have been successfully electropolymerized on a glassy carbon electrode, giving a robust electroactive film. Additionally, we show that alkyne-PEDOT can also be post-functionalized in a “click” fashion. All of these proved the synthetic and electrochemical utilities of pyEDOT. Thus, we believe that the pyEDOT synthon provides valuable starting points for future new functionalized EDOT monomers and following polymer or oligomer research.

## Supporting Information

File 1 Experimental details, NMR spectra, IR spectra, and HRMS for all products and electrochemistry data.
