# Supplementary material for: A versatile route to polythiophenes with functional pendant groups using alkyne chemistry
Source: Beilstein J Org Chem. 2016 Dec 9;12:2682–8. doi: 10.3762/bjoc.12.265 (PMC5238539; doi:10.3762/bjoc.12.265)
Supplement: File 1 — Experimental details, NMR spectra, IR spectra, and HRMS for all products and electrochemistry data. [file Beilstein_J_Org_Chem-12-2682-s001.pdf]

Supporting Information

for

A versatile route to polythiophenes with functional pendant groups using alkyne chemistry

Xiao Huang*[1], Li Yang[2], Rikard Emanuelsson[2], Jonas Bergquist[1], Maria Strømme[2], Martin Sjödin[2] and Adolf Gogoll*[1]

Address: [1]Department of Chemistry - BMC, Uppsala University, Box 576, 751 23 Uppsala, Sweden and [2]Nanotechnology and Functional Materials, Department of Engineering Sciences, Uppsala University, Box 534, 751 21 Uppsala, Sweden.

Email: Adolf Gogoll - Adolf.gogoll@kemi.uu.se; Xiao Huang - xiao.huang@kemi.uu.se

*Corresponding author

Experimental details, NMR spectra, IR spectra, and HRMS for all products and electrochemistry data

Contents

Experimental section S2
NMR spectra S16
IR spectra S41
HRMS spectra S50
Eletrochemistry data S59
References S61

Experimental section

General information - organic chemistry. All solvents and other commercially available chemicals were used as received. NMR spectra were recorded on a Varian INOVA (^{1}H at 499.93 MHz, ^{13}C at 125.7 MHz), an Agilent MR (^{1}H 399.97 at MHz, ^{13}C at 100.58 MHz), or a Varian MercuryPlus (^{1}H at 299.9 MHz, ^{13}C at 75.4 MHz) spectrometer. Chemical shifts are reported in δ (ppm) relative to tetramethylsilane (TMS) via the residual solvent signals: $CDCl_3$, ^{1}H at 7.26 and ^{13}C at 77.16 ppm; $(CD_3)_2CO$, ^{1}H at 2.05 and ^{13}C at 29.8 ppm. The following abbreviations were used: s = singlet, d = doublet, t = triplet, q = quartet, m = multiplet, dd = doublet of doublets, br = broad. Signal assignments were derived from COSY [1-2], P.E.COSY [3], gHSQC [4], gHMBC [5], gNOESY [6], ROESY [7], and TOCSY [8] spectra. For clarity, assignments refer to consecutive atom numbers, as indicated in this Supplementary material.

Solvents for extraction and chromatography were technical grade and were used without further purification. Dry tetrahydrofuran (THF) was obtained by passing through activated alumina columns in a solvent purification system. All other solvents used for reactions were analytical grade. Flash chromatography was performed using silica gel (200 ± 300 mesh). IR spectra were recorded with a FTIR spectrometer with UATR accessory. For mass spectra, samples were dissolved in a mixture of water/methanol/formic acid 49:50:1, and 10 μL of this solution were introduced via spray needle into an LTQ Orbitrap Velos Pro ETD mass spectrometer using a spray voltage of 1.8 kV in the *m*/*z* range of 100–1200. The resolution was 60 000 in the Fourier Transform analyzer (Orbitrap). Spectra were collected for 2–3 min.

General information - electrochemistry. Cyclic voltammograms (CVs) of the monomers and their polymers were recorded on a PGSTAT302N potentiostat. The polymers were prepared by electrochemical polymerization as reported previously [9]. A glassy carbon (0.3 mm in diameter) electrode and a platinum electrode were utilized as the working and counter

electrode, respectively. The reference electrode was a compartment with $Ag^+(AgNO_3)/Ag^0$ redox species, which was calibrated against the ferrocene/ferrocenium (Fc^+/Fc^0) redox couple and, unless stated otherwise, potentials are given against Fc^+/Fc^0 throughout this report. All polymers were prepared from an acetonitrile (MeCN) solution containing the monomer and tetraethylammonium hexafluorophosphate ($TEAPF_6$, 0.1 M). The monomer solution was purged with solvent saturated N_2 thoroughly prior to the polymerization and N_2 purging was maintained throughout all electrochemical processes. CVs of the monomers were acquired prior to the polymerization of each monomer. Post-functionalization of poly(pyEDOT) with azide-phthalimide was performed as follows: a Pt-disk electrode coated with poly(pyEDOT) film was dipped into an MeCN solution (2 mL) with 0.6 mmol azide-phthalimide and 5 mol % $Cu(CH_3CN)_4PF_6$ and 0.3 mmol copper powder for 3 days. The electrode was then washed with ethanol and acetone thoroughly before further characterization.

3-(EDOT) prop-1-yne (pyEDOT) (**3**). ***a)*** Glycidol (9.45 g, 128 mmol) was added to a solution of *tert*-butyldimethylsilyl chloride (25.0 g, 166 mmol) and imidazole (13.9 g, 204 mmol) in DMF (80 mL) at 0 °C. The reaction mixture was stirred at 0 °C for 30 minutes, during which time a white precipitate had formed, then allowed to warm to room temperature and stirred for 2 hours. Saturated aqueous NaCl solution (100 mL) was added and the mixture was stirred for an additional 20 minutes. The product was extracted with diethyl ether (3 × 50 mL). The combined organic extracts were washed with water (2 × 200 mL) and saturated aqueous NaCl solution (100 mL) then concentrated in vacuo. The substrate was purified by flash column chromatography (SiO_2; petroleum ether/ethyl acetate, 100:0 to 97:3), yielding after solvent removal in vacuo the *tert*-butyldimethyl(oxiran-2-ylmethoxy)silane (**1**) as a colourless oil (20.7 g, 86%). ^1H NMR (499.93 MHz, $CDCl_3$, 25 °C): δ = 3.85 (dd, *J* = 3.2, 11.9 Hz, 1H), 3.66 (dd, *J* = 4.8, 11.9 Hz, 1H), 3.08 (m, 1H), 2.77 (dd, *J* = 4.0, 5.3 Hz, 1H), 2.63 (dd, *J* = 2.7, 5.3 Hz, 1H), 0.90 (s, 9H), 0.08 (s, 3H), 0.07 (s, 3H). Data are in accordance

with the literature [10]. ***b)*** Trimethylsilylacetylene (17.0 mL, 120 mmol) was added to a solution of *n*-BuLi (48.0 mL, 120 mmol, 2.5 M in hexane) in THF (120 mL) at −78 °C. The reaction mixture was stirred at −78 °C for 40 minutes, then a solution of protected epoxide **1** (19.7 g, 104 mmol) in THF (30 mL) was added, followed by $BF_3·OEt_2$ (15.1 mL, 120 mmol) at −78 °C. The reaction mixture was stirred at −78 °C for 1 hour then allowed to warm to room temperature and stirred for 5 hours. Saturated aqueous NH_4Cl solution (100 mL) was added and the resulting biphasic mixture was separated. The aqueous layer was extracted with diethyl ether (3 × 100 mL). The combined organic extracts were dried over $MgSO_4$ and concentrated in vacuo. The residue was dissolved in methanol (300 mL). Acetyl chloride (0.75 mL, 10.4 mmol) was added and the reaction mixture was stirred for 12 hours. Thereafter, K_2CO_3 (28.9 g, 209 mmol) was added and the reaction mixture was stirred for another 6 hours. The reaction mixture was filtered through a pad of celite then concentrated in vacuo. Saturated aqueous NaCl solution (30 mL) was added and the product was extracted with ethyl acetate (8 × 100 mL). The combined organic extracts were dried over $MgSO_4$ then concentrated in vacuo. The substrate was purified by flash column chromatography (SiO_2; petroleum ether/ethyl acetate, 2:1 to ethyl acetate), followed by solvent removal in vacuo, yielding the pent-4-yne-1,2-diol (**2**) as a colourless oil (5.2 g, 50%). ^{1}H NMR (499.93 MHz, $CDCl_3$, 25 °C): δ = 3.90 (m, 1H), 3.76 (dd, *J* = 3.6, 11.3 Hz, 1H), 3.61 (dd, *J* = 6.4, 11.3 Hz, 1H), 2.50 (bs, 1H), 2.45 (dd, *J* =1.7, 2.7 Hz, 1H), 2.44 (dd, *J* = 1.4, 2.7 Hz, 1H), 2.08 (bs, 1H), 2.06 (t, 2.7 Hz, 1H). Data are in accordance with the literature[10]. ***c)*** In an oven-dried round bottom flask filled with argon gas, 3,4-dimethoxythiophene (3.8 g, 26.3 mmol) was dissolved in toluene (90 mL). To the solution was added toluene-4-sulfonic acid (*p*-TSA, 0.5 g, 2.64 mmol) and diol **2** (2.6 g, 26 mmol), and the solution was heated at 90 °C for 1 day. A second portion of diol **2** (2.6 g, 26 mmol) was added to the warm mixture and it was kept stirring at 90 °C for a second day. The resulting solution was filtered through a pad of celite

and concentrated under vacuum. The crude product was obtained as green-black oil. It was purified by flash column chromatography (SiO_2; petroleum ether/dichloromethane, 4:1), yielding after solvent removal in vacuo the 3-(EDOT)prop-1-yne (pyEDOT) **3** as a yellow oil (3.06 g, 64%). ^1H NMR (299.9 MHz, $CDCl_3$, 25 °C): δ = 6.34 (s, 2H, H-1,4), 4.33-4.29 (m, 2H, H-7,8), 4.06 (dd, *J* = 7.6, 11.9 Hz, 1H, H-7'), 2.68 (ddd, *J* = 2.7, 5.3, 17.0 Hz, 1H, H-12), 2.56 (ddd, *J* = 2.7, 7.6, 17.0 Hz, 1H, H-12'), 2.10 (t, *J* = 2.7 Hz, 1H, H-11). ^{13}C NMR (75.4 MHz, $CDCl_3$, 25 °C): δ = 141.5 (C-3), 141.4 (C-2), 100.0 (C-1/4), 99.9 (C-1/4), 78.5 (C-10), 71.8 (C-8), 71.5 (C-11), 67.3 (C-7), 21.2 (C-12). IR (neat, $\tilde{\nu}$/cm^{-1}): 3287, 2917, 1480, 1182, 1016, 756. HRMS, calculated for $C_9H_9O_2S$: m/z = 181.03178, found: *m/z* = 181.03095 ([M + H^+]).

2-Ethynyl-2,3-dihydrothieno[3,4-*b*][1,4]dioxine (eEDOT) (8). ***a)*** Saturated aqueous $NaHCO_3$ (12 mL) and a solution of 1,2:5,6-bis-O-(1-methylethylidene)-D-mannitol (30.0 g, 114 mmol) in dichloromethane (200 mL) were placed in a round bottom flask (500 mL) equipped with a mechanical stirrer. $NaIO_4$ (48.9 g, 229 mmol) was added in portions, and the solution was stirred for 1.5 hours at room temperature. The reaction mixture was filtered through a pad of celite and the organic filtrate was condensed in vacuo to afford crude 2,2-dimethyl-1,3-dioxolane-4-carbaldehyde (**4**, 27.4 g). ^1H NMR (499.93 MHz, $CDCl_3$, 25 °C): δ = 9.72 (d, *J* = 1.9 Hz, 1H), 4.39 (ddd, *J* = 7.0, 4.7, 1.9 Hz, 1H), 4.17 (dd, *J* = 8.8, 7.0 Hz, 1H), 4.10 (dd, *J* = 8.8, 4.7 Hz, 1H), 1.49 (s, 3H), 1.42 (s, 3H). Data are in accordance with the literature [11]. The product was directly used for the next step without further purification. ***b)*** A 1 L round bottom flask was charged with a stir bar and a solution of triphenylphosphine (220.6 g, 841 mmol) in DCM (400 mL). The mixture was stirred and cooled to 0 °C, after which tetrabromomethane (139.5 g, 421 mmol) was added over a period of 30 min. The reaction was allowed to warm to room temperature under stirring for 30 minutes. It then was cooled to 0 °C, and a solution of aldehyde **4** (27.4 g, 144 mmol) in DCM (30 mL) was added.

The reaction mixture was allowed to warm to room temperature overnight, thereafter cooled to 0 °C, and hexane (300 mL) was added. The mixture was allowed to stir for 1 hour and then filtered through a pad of celite. The remaining insoluble residue was dissolved by as small an amount of DCM as possible and then hexane (200 mL) was added. The mixture was filtered again through a pad of celite. The two combined filtrates were condensed in vacuo. To the residue was added hexane (250 mL), generating an insoluble white solid, which was removed by filtration through a pad of celite. The solution was condensed in vacuo, affording 4-(2,2-dibromovinyl)-2,2-dimethyl-1,3-dioxolane (**5**) as a clear oil (42.2 g, 61 %, purity approx. 97% according to ^{1}H NMR). ^{1}H NMR (499.93 MHz, $CDCl_3$, 25 °C): δ = 6.53 (d, J = 7.6 Hz, 1H), 4.73 (ddd, J = 7.6, 6.5, 6.3 Hz, 1H), 4.19 (dd, J = 8.4, 6.3 Hz, 1H), 3.69 (dd, J = 8.4, 6.5 Hz, 1H), 1.43 (s, 3H), 1.38 (s, 3H). Data are in accordance with the literature[12]. The product was used without further purification in the next step. ***c)*** In a round bottom Schlenk flask (500 mL) under nitrogen atmosphere dibomoolefin **5** (28.93 g, 101 mmol) was dissolved in dry THF (100 mL). The solution was cooled to −78 °C and *n*-butyllithium (88 mL of 2.5 M solution in hexane, 220 mmol) was slowly added. The mixture was stirred at −78 °C for 1 hour and then allowed to warm to rt under stirring for another 1 hour. Thereafter, it was quenched with water (100 mL) and then extracted with diethyl ether (2 × 100 mL). The combined organic layers were washed with brine (2 × 100 mL) and dried over $MgSO_4$. The solvent was removed in vacuo to yield crude 4-ethynyl-2,2-dimethyl-1,3-dioxolane (**6**) as a clear oil (11.6 g). ^{1}H NMR (499.93 MHz, $CDCl_3$, 25 °C): δ = 4.70 (ddd, J = 6.4, 6.2, 2.1 Hz, 1H), 4.17 (dd, J = 8.1, 6.4 Hz, 1H), 3.95 (dd, J = 8.1, 6.2 Hz, 1H), 2.49 (d, J = 2.1 Hz, 1H), 1.50 (s, 3H), 1.38 (s, 3H). Data are in accordance with the literature [13]. The product was used directly for the next step without purification. ***d)*** In a round bottom flask (250 mL) equipped with a condenser alkyne **6** (11.6 g, 91.9 mmol) and pSTA (0.875 g, 4.60 mmol) were dissolved in methanol (80 mL). The mixture was heated at 40 °C overnight, thereafter

condensed in vacuo and the brown residue was dissolved in ethyl acetate (100 mL). The solution was washed with sat. $NaHCO_3$ (30 mL) and brine (2 × 80 mL), and dried over $MgSO_4$. The organic layer was condensed in vacuo to give crude product as a yellow oil. This product was purified by flash column chromatography (SiO_2; pentane/ethyl acetate, 1:1 to 2:3) and yielded but-3-yne-1,2-diol **7** as a white solid (4.59 g, 32 %). ^{1}H NMR (499.93 MHz, $CDCl_3$, 25 °C): δ = 4.47 (m, 1H), 3.74 (m, 2H), 2.53 (br, 1H), 2.50 (d, J = 1.8 Hz, 1H), 2.2 (br, 1H). Data are in accordance with the literature [14]. ***e)*** In an oven-dried round bottle flask filled with argon 3,4-dimethoxythiophene (2.0 g, 13.9 mmol) and diol **7** (1.19 g, 13.8 mmol) were dissolved in toluene (60 mL). To the mixture was added pTSA (0.26 g, 1.39 mmol) and the solution was heated at 90 °C for 1 day. A second portion of diol **7** (1.19 g, 13.8 mmol) was added to the warm mixture which was kept stirring at 90 °C for another three days. The resulting solution was filtered through a pad of celite and concentrated under vacuum. The crude product appeared as green-black oil. It was purified by flash column chromatography (SiO_2; petroleum ether/dichloromethane, 4:1) to yield the ethynyl-(EDOT) (eEDOT) **8** as colorless oil (0.145 g, 6.3 %). ^{1}H NMR (499.93 MHz, $CDCl_3$, 25°C): δ = 6.41 (d, J = 3.8 Hz, 1H, H-1/4), 6.37 (d, J = 3.8 Hz, 1H, H-1/4), 4.90 (dt, J = 6.6, 2.3 Hz, 1H, H-7), 4.27 (dd, J = 11.6, 2.3 Hz, 1H, H-8), 4.13 (dd, J = 11.6, 6.6 Hz, 1H, H-8'), 2.61 (d, J = 2.3 Hz, 1H, H-11). ^{13}C NMR (75.4 MHz, $CDCl_3$, 25 °C): δ = 140.8 (C-2/3), 140.4 (C-2/3), 101.0 (C-1/4), 100.4 (C-1/4), 77.3 (C-10), 76.5 (C-7), 67.6 (C-8), 64.3 (C-11). IR (neat, $\tilde{\nu}$/cm^{-1}): 3264, 2963, 1482, 1258, 1011, 788. HRMS, calculated for $C_8H_7O_2S$: m/z = 167.01613, found: m/z = 167.01602 ([M + H$^+$]).

((2,3-Dihydrothieno[3,4-*b*][1,4]dioxin-2-yl)ethynyl)trimethylsilane (etEDOT) (8'). ***a)*** In a round bottom Schlenk flask (250 mL) under nitrogen atmosphere dibromoolefin **5** (7.24 g, 25.3 mmol) was dissolved in dry THF (60 mL). The solution was cooled to −78 °C and *n*-butyllithium (22 mL of 2.5 M solution in hexane, 54.9 mmol) was slowly added. The mixture

was stirred at −78 °C for 1 hour and then allowed to warm to rt while stirring for another 1 hour. The solution was then cooled to −78 °C. Chlorotrimethylsilane (3.3 g, 30.4 mmol) was slowly added, and the solution was allowed to warm to rt overnight. The reaction mixture was separated between diethyl ether (150 mL) and water (100 mL), and the organic layer was washed with brine (3 × 50 mL) and dried over $MgSO_4$. The solvent was removed in vacuo, affording the pure ((2,2-dimethyl-1,3-dioxolan-4-yl)ethynyl)trimethylsilane (**6'**) as colorless oil (4.2 g, 84 %). ^1H NMR (499.93 MHz, $CDCl_3$, 25 °C): δ = 4.70 (dd, J = 6.6, 6.3 Hz, 1H, H-2), 4.14 (dd, J = 8.0, 6.3 Hz, 1H, H-1), 3.89 (dd, J = 8.0, 6.6 Hz, 1H, H-1'), 1.49 (s, 3H, H-8/9), 1.37 (s, 3H, H-8/9), 0.17 (s, 9H, H-11,12,13). ^{13}C NMR (125.7 MHz, $CDCl_3$, 25 °C): δ = 104.5 (C-6), 96.7 (C-3), 85.2 (C-4), 64.2 (C-2), 60.1 (C-1), 20.4 (C-8/9), 20.2 (C-8/9), -6.1 (C-11,12,13). IR (neat, $\tilde{\nu}$/cm^{-1}): 2959, 2928, 2856, 2177, 1711, 1250, 1060. HRMS, calculated for $C_{10}H_{19}O_2Si$: m/z = 199.11488, found: m/z = 199.11482 ([M + H^+]). ***b)*** In a round bottom flask (250 mL) equipped with a condenser TMS-protected alkyne **6'** (2.0 g, 10.1 mmol) and *p*-STA (96.0 mg, 0.504 mmol) were dissolved in methanol (80 mL) and the mixture was heated at 40 °C overnight. The solvent was removed in vacuo and the brown residue was dissolved in ethyl acetate (100 mL), the resulting solution washed with sat. aqueous $NaHCO_3$ (30 mL) and brine (2 × 80 mL), and dried over $MgSO_4$. The organic solvent was then removed in vacuo, giving the crude product as a yellow oil. This was purified by flash column chromatography (SiO_2; pentane/ethyl estate, 1:1 to 2:3), yielding 4-(trimethylsilyl)but-3-yne-1,2-diol (**7'**) as a white solid (0.5 g, 31 %). ^1H NMR (499.93 MHz, $CDCl_3$, 25 °C): δ = 4.43 (dd, J = 7.4, 3.5 Hz, 1H), 3.72 (dd, J = 11.5, 3.5 Hz, 1H), 3.63 (dd, J = 11.5, 7.4 Hz, 1H), 3.63 (br, 1H), 3.34 (br., 1H), 0.16 (s, 9H). ^{13}C NMR (125.7 MHz, $CDCl_3$, 25 °C): δ = 103.2, 91.2, 66.5, 63.7, −0.1. Data is in accordance with the literature [15]. ***c)*** In an oven-dried round bottle flask filled with argon 3,4-dimethoxythiophene (250 mg, 1.73 mmol) and diol **7'** (250 mg, 1.08 mmol) were dissolved in dichloroethane (15 mL). To the

mixture was added pTSA (33 mg, 0.173 mmol) and the solution was heated at 90 °C for 1 day. A second portion of **7'** (250 mg, 1.08 mmol) was added to the warm mixture and it was kept stirring at 90 °C for another day. The resulting solution was filtered through a pad of celite and concentrated in vacuo, resulting in the crude product as a green-black oil. It was purified by flash column chromatography (SiO_2; petroleum ether/dichloromethane, 4:1) and yielded the ethynyltrimethylsilane-(EDOT) (etEDOT) (**8'**) as colorless oil (50 mg, 12 %). ^{1}H NMR (499.93 MHz, $CDCl_3$, 25 °C): δ = 6.39 (d, J = 3.7 Hz, 1H, H-1/4), 6.34 (d, J = 3.7 Hz, 1H, H-1/4), 4.85 (dd, J = 8.1, 2.4 Hz, 1H, H-7), 4.27 (dd, J = 11.6, 2.4 Hz, 1H, H-8), 4.07 (dd, 11.6, 8.1 Hz, 1H, H-8'), 0.2 (s, 9H, H-13,14,15). ^{13}C NMR (125.7 MHz, $CDCl_3$, 25 °C): δ = 141.0 (C-2/3), 140.8 (C-2/3), 100.7 (C-1/4), 100.1 (C-1/4), 97.8 (C-10), 94.3 (C-11), 67.8 (C-7), 65.2 (C-8), -0.22 (C-13,14,15). IR (neat, $\tilde{\nu}$/cm^{-1}): 3116, 2960, 2183, 1481, 1183, 1056, 839. HRMS, calculated for $C_{11}H_{15}O_2SSi$: m/z = 239.05565, found: m/z = 239.05561 ([M + H^+]).

Diethyl 2-(3-(2,3-dihydrothieno[3,4-*b*][1,4]dioxin-2-yl)prop-1-yn-1-yl)terephthalate (**9**). In a Schlenk round bottom flask pyEDOT (329 mg, 1.83 mmol) and 2-bromoterephthalic ethyl ester [16] (500 mg, 1.66 mmol) were dissolved in dry THF (15 mL). The solution was degassed with nitrogen for 30 minutes. CuI (32 mg, 0.166 mmol), $Pd(PPh_3)_4$ (58 mg, 0.050 mmol) and triethylamine (336 mg, 3.32 mmol) were then consecutively added under nitrogen atmosphere. The flask was closed with a glass stopper and the mixture was heated at 75 °C for 24 hours. The reaction mixture was filtered through a pad of celite. The filtrate was separated between diethyl ether (20 mL) and sat. aqueous NH_4Cl solution (20 mL). The organic layer was then washed with brine (2 × 20 mL) and dried over $MgSO_4$. The organic solvent was removed in vacuo, affording crude product as dark brown oil. The oil was purified by flash column chromatography (SiO_2; pentane/ethyl acetate, 4:1), yielding **9** as white solid (404 mg, 61 %). ^{1}H NMR (499.93 MHz, $CDCl_3$, 25 °C): δ = 8.16 (d, J = 1.4 Hz, 1H, H-14), 7.99 (dd, J

= 1.4, 8.2 Hz, 1H, H-16), 7.95 (d, J = 8.2 Hz, 1H, H-17), 6.36 (d, J = 3.7 Hz, 1H, H-1/4), 6.35 (d, J = 3.7 Hz, 1H, H-1/4), 4.46 - 4.42 (m, 2H, H-7,8), 4.40 (q, J = 5.8 Hz, 2H, H-25/27), 4.39 (q, J = 5.8 Hz, 2H, H-25/27), 4.20 (m, 1H, H-7), 2.97 (dd, J = 5.0, 17.1 Hz, 1H, H-12), 2.86 (dd, J = 8.6, 17.1 Hz, 1H, H-12'), 1.41 (t, J = 5.8 Hz, 3H, H-26/28), 1.40 (t, J = 5.8 Hz, 3H, H-26/28). ^{13}C NMR (125.7 MHz, $CDCl_3$, 25 °C): δ= 165.6 (C-19), 165.3 (C-22), 141.6 (C-3), 141.4(C-2), 136.0 (C-18), 135.3 (C-14), 133.3 (C-15), 130.4 (C-17), 128.7 (C-16), 123.8 (C-13), 100.0 (C-1/4), 99.5 (C-1/4), 90.1 (C-10), 81.4 (C-11), 72.0 (C-8), 67.4 (C-7), 61.8 (C-25/27), 61.7 (C-25/27), 22.4 (C-12), 14.4 (C-26,28). IR (neat, $\tilde{\nu}$/cm^{-1}): 3116, 2983, 2230, 1718, 1486, 1017, 750. *mp* 87-90 °C. HRMS, calculated for $C_{21}H_{21}O_6S$: *m/z* = 401.10534, found: *m/z* = 401.10564 ([M + H$^+$]); calculated for $C_{21}H_{20}NaO_6S$: *m/z* = 423.08728, found: *m/z* = 423.08729 ([M + Na$^+$]).

2-(3-(2,3-Dihydrothieno[3,4-*b*][1,4]dioxin-2-yl)prop-1-yn-1-yl)-4,4'-bipyridine (**10**) and **2-(3-(2,3-dihydrothieno[3,4-*b*][1,4]dioxin-2-yl)prop-1-yn-1-yl)-*N*,*N*'-dimethylviologen hexafluorophosphate(V)** (**11**). ***a)*** Under Ar atmosphere *n*-BuLi (12.78 mL of a 2.5 M solution in hexanes 31.96 mmol) was added to a solution of iPr_2NH (4.44 mL, 31.96 mmol) in THF (100 mL) at −45 °C. After stirring for 0.5 h, the reaction mixture was further cooled to −95 °C (toluene/liquid N_2), and 2-bromopyridine (3.02 mL, 31.65 mmol) was added. The resulting mixture was stirred at -95 °C to −90 °C for 3 to 4 h and I_2 (8.03 g, 31.65 mmol) was added in portions. After slowly warming to rt overnight, the mixture was quenched with 10% aq. HOAc solution (pH 8–9) and then distilled water (50 mL) was added. The organic material was extracted with diethyl ether and the resulting solution was dried over anhydrous K_2CO_3. The solvent was evaporated in vacuo and the crude 2-bromo-3-iodopyridine was obtained as a brown powder. The brown powder was then dried in vacuo in a desicator together with KOH pellets over 3 hours. The 2-bromo-3-iodopyridine (7.66 g, 85%) was highly pure as judged from ^{1}H NMR and it was directly used for the next step. ^{1}H NMR

(499.93 MHz, $CDCl_3$, 25 °C): δ = 8.05 (dd, J = 0.6, 5.2 Hz, 1H), 7.91 (dd, J = 0.6, 1.5 Hz, 1H), 7.62 (dd, J = 1.5, 5.2 Hz, 1H). Data are in accordance with the literature [17]. ***b)*** 4-In a heavy-walled Smith process vial (25 mL) iodo-2-bromo-pyridine (400 mg, 1.41 mmol) and pyridin-4-ylboronic acid (173 mg, 1.41 mmol) were dissolved in a mixture of iPrOH (8 mL) and DME (2 mL). The solution was degassed with nitrogen over 30 minutes. A N_2-purged aqueous Na_2CO_3 solution (2 M, 1.4 mL, 2.82 mmol) and $Pd(PPh_3)_4$ (49 mg, 0.04 mmol) were added to the above mixture. The vial was sealed with a Teflon cap under N_2 atmosphere and was heated at 100 °C in an oil bath for 36 h. The reaction mixture was diluted with DCM (40 mL), filtered through a pad of celite, the filtrate was washed with 0.1 M NaOH solution (2 × 20 mL) and brine (2 × 20 mL), and the organic layer was dried over $MgSO_4$. The solvent was removed in vacuo, affording crude product as a white yellow solid (320 mg). The crude product contained triphenylphospine oxide as an impurity which is known for its difficult chromatographic removability. ^{1}H NMR quantification indicated 88% purity of the obtained 2-bromo-4,4'-bipyridine that was used for the next step without further purification. ^{1}H NMR (499.93 MHz, $CDCl_3$, 25 °C): δ = 8.80 - 8.78 (m, 2H), 8.53 (d, J = 5.2 Hz, 1H), 7.75 (d, J = 1.6 Hz, 1H), 7.62 - 7.59 (m, 2H), 7.50 (dd, J = 1.6, 5.2 Hz, 1H). Data are in accordance with the literature [18]. ***c)*** In a heavy-walled Smith process vial (25 mL) 2-bromo-4,4'-bipyridine (246 mg, 88 %, 0.923 mmol) and pyEDOT (183 mg, 1.02 mmol) were dissolved in dry THF (15 mL). The solution was degassed with nitrogen for 30 minutes. CuI (18 mg, 0.092 mmol), $Pd(PPh_3)_4$ (28 mg, 0.028 mmol) and TEA (0.258 mL, 1.85 mmol) were consecutively added to the mixture under N_2 atmosphere. The heavy-walled Smith process vial was sealed with a Teflon cap under N_2 atmosphere and was heated at 70 °C in an oil bath overnight. The reaction mixture was filtered through a pad of celite, and the filtrate was evaporated in vacuo and the residue was dissolved in DCM (30 mL). After washing with 1 M NaOH (2 × 15 mL) followed by brine (2 × 20 mL) the organic layer was dried over anhydrous Na_2CO_3. Removal

of solvent in vacuo afforded crude product as a brown oil. This oil was purified by column chromatography (SiO_2; DCM/Methanol, 20:1). To remove the triphenylphosphine oxide impurity, the chromatographed product was dissolved in DCM (20 mL) and to this solution was added anhydrous $MgCl_2$ (ca. 1 g). The mixture was allowed to stir at rt overnight, and then filtered through a pad of celite. Solvent was removed from the filtrate in vacuo to afford pyEDOT derivative **10** as a yellow waxy oil (280 mg, 82%). ^{1}H NMR (499.93 MHz, $CDCl_3$, 25 °C): δ = 8.72 (dd, *J* = 1.7, 4.4 Hz, 2H, H-9,11), 8.64 (dd, *J* = 0.9, 5.2 Hz, 1H, H-1), 7.63 (dd, *J* = 0.9, 1.8 Hz, 1H, H-4), 7.49 (dd, *J* = 1.7, 4.4 Hz, 2H, H-8,12), 7.43 (dd, *J* = 1.8, 5.2 Hz, 1H, H-6), 6.33 (d, *J* = 3.6 Hz, 1H, H-22/24) 6.32 (d, *J* = 3.6 Hz, 1H, H-22/24), 4.4 (m, 1H, H-16), 4.34 (dd, *J* = 2.2, 11.6 Hz, 1H, H-21), 4.11 (dd, *J* = 7.1, 11.6 Hz, 1H, H-21'), 2.93 (dd, *J* = 5.3, 17.0 Hz, 1H, H-15), 2.83 (dd, *J* = 8.1, 17.0 Hz, 1H, H-15'). ^{13}C NMR (125.7 MHz, $CDCl_3$, 25 °C): δ = 150.8 (C-1), 150.7 (C-9,11), 146.0 (C-5), 144.8 (C-7), 144.0 (C-3), 141.3 (C-18/19), 141.2 (C-18/19), 124.8 (C-4), 121.4 (C-8,12), 120.6 (C-6), 100.0 (C-22/24), 99.9 (C-22/24), 85.2 (C-14), 82.6 (C-13), 71.6 (C-16), 67.3 (C-21), 22.0 (C-15). R (neat, $\tilde{v}$/cm^{-1}): 3110, 3042, 2914, 2234, 1585, 1481, 1182, 1066, 811, 757. APCI-MS: 335.06 (M+H$^+$). HRMS, calculated for $C_{19}H_{15}N_2O_2S$: *m/z* = 335.08487, found: *m/z* = 335.08455 ([M + H$^+$]). d) In a heavy-walled Smith process vial under nitrogen atmosphere bipyridine-pyEDOT **10** (48 mg, 0.144 mmol) was dissolved in dry DCM (3 mL). Methyl iodide (0.09 mL, 1.43 mmol) was pre-treated with anhydrous K_2CO_3 before it was added to this solution. The vial was sealed with a Teflon cap and heated at 50 °C for 24 h. The viologen iodide product precipitated from solution as a red colored solid during evaporation of the solvent in vacuo. The red precipitate was then washed with DCM using an ultrasound bath followed by centrifugation (3×). The washed solid was dried on a filter paper after suction filtration yielding the viologen iodide (40 mg, 0.065 mmol). The thus isolated red solid was dissolved in water (5 mL), and the solution was slowly added to an aqueous solution of NH_4PF_6 (63 mg,

0.388 mmol in 5 mL). A yellow precipitate was quickly formed within a few minutes. This precipitate was collected on a filter paper using suction filtration, then washed with water (3 × 10 mL). The resulting solid was dried on the filter paper by applying vacuum suction overnight, yielding pyEDOT derivative **11** as a yellow solid, *mp* 168-171 °C (45 mg, 40%). ^{1}H NMR (499.93 MHz, $(CD_3)_2CO$, 25 °C): δ = 9.38 (d, *J* = 6.5 Hz, 1H, H-31), 9.37 (d, *J* = 6.1 Hz, 2H, H-35,37), 8.95 (d, *J* = 1.6 Hz, 1H, H-28), 8.84 (d, *J* = 6.1 Hz, 2H, H-34,38), 8.73 (dd, *J* = 1.6, 6.5 Hz, 1H, H-30), 6.52 (d, *J* = 3.6 Hz, 1H, H-15/18), 6.51 (d, *J* = 3.6 Hz, 1H, H-15/18), 4.73 (s, 3H, H-40), 4.71 (s, 3H, H-39), 4.63 (m, 1H, H-22), 4.42 (dd, *J* = 2.3, 12.0 Hz, 1H, H-21), 4.19 (dd, *J* = 7.2, 12.0 Hz, 1H, H-21'), 3.35 (dd, *J* = 5.7, 18.0 Hz, 1H, H-26), 3.29 (dd, *J* = 6.4, 18.0 Hz, 1H, H-26'). ^{13}C NMR (100.58 MHz, $(CD_3)_2CO$, 25 °C): δ = 150.3 (C-29), 150.11 (C-33), 149.1 (C-31), 147.9 (C-35,37), 142.2 (C-16/17), 142.1 (C-16/17), 140.0 (C-27), 131.4 (C-28), 127.7 (C-34，38), 126.0 (C-30), 108.3 (C-24), 100.9 (C-15/18), 100.7 (C-15/18), 74.7 (C-25), 71.9 (C-22), 67.8 (C-21), 49.5 (C-40), 48.5 (C-39), 23.0 (C-26). IR (neat, ν/cm^{-1}): 3118, 2244, 1637, 1486, 1194, 829. HRMS, calculated for $C_{21}H_{20}N_2O_2S$: *m/z* = 182.06173, found: *m/z* = 182.06175 ([M^{2+}]).

2-(3-(2,3-Dihydrothieno[3,4-*b*][1,4]dioxin-2-yl)prop-1-yn-1-yl)anthracene-9,10-dione (**12**). In a heavy-walled Smith process vial (25 mL) 2-bomoanthraquinone (300 mg, 1.05 mmol) and pyEDOT (207 mg, 1.15 mmol) were dissolved in dry THF (15 mL). The solution was then degassed with nitrogen over 30 minutes. CuI (20 mg, 0.104 mmol), $Pd(PPh_3)_4$ (36 mg, 0.031 mmol) and TEA (0.3 mL, 2.09 mmol) were consecutively added. The vial was sealed with a Teflon cap under nitrogen atmosphere, and heated at 75 °C in an oil bath overnight. The resulting black slurry was filtered through a pad of celite by suction filtration. The filtrate was concentrated under vacuum, affording the crude product as a black-brown solid. This solid was purified through column chromatography (SiO_2; pentane/DCM, 1:2) to afford pyEDOT derivative **12** as a yellow solid, *mp* 165-169 °C. (290 mg, 65%). ^{1}H NMR

(499.93 MHz, $CDCl_3$, 25 °C) δ = 8.33 – 8.30 (m, 3H, H-14,23,26), 8.26 (dd, *J* = 0.5, 8.0 Hz, 1H, H-17), 7.81 (m, 2 H, H-24,25), 7.79 (dd, *J* = 1.7, 8.0 Hz, 1H, H-18), 6.38 (d, *J* = 3.6 Hz, 1H, H-1/4), 6.37 (d, *J* = 3.6 Hz, 1H, H-1/4), 4.43 (m, 1H, H-8), 4.37 (dd, *J* = 2.2, 11.6 Hz, 1H, H-7), 4.15 (dd, *J* = 7.1, 11.6 Hz, 1H, H-7'), 2.97 (dd, *J* = 5.5, 17.2 Hz, 1H, H-12), 2.88 (dd, *J* = 7.9, 17.2 Hz, 1H, H-12'). ^{13}C NMR (125.7 MHz, $CDCl_3$, 25 °C): δ = 182.7 (C-19), 182.6 (C-22), 141.4 (C-2/3), 141.3 (C-2/3), 136.8 (C-18), 134.5 (C-24/25), 134.4 (C-24/25), 133.6 (C-20/21), 133.5 (C-20/21), 133.5 (C-15), 132.6 (C-16), 130.5 (C-14), 129.4 (C-13), 127.5 (C-23/26), 127.4 (C-23/26), 100.2 (C-1/4), 100.1 (C-1/4), 89.3 (C-10), 82.3 (C-11), 71.8 (C-8), 67.4 (C-7), 22.3 (C-12). IR (neat, ṽ/cm^{-1}): 2919, 1672, 1591, 1485, 1304, 709. APCI-MS: 387.59 ($M+H^+$). HRMS, calculated for $C_{23}H_{15}O_4S$: *m/z* = 387.06856, found: *m/z* = 387.06828 ([M + H^+]).

2-(2-(4-((2,3-Dihydrothieno[3,4-*b*][1,4]dioxin-2-yl)methyl)-1*H*-1,2,3-triazol-1-yl)ethyl)isoindoline-1,3-dione (13). a) In a round bottom flask (100 mL) *N*-(2-bromoethyl) phthalimide (1.00 g, 3.94 mmol) was dissolved in dry DMF (20 mL). Sodium azide (281 mg, 4.3 mmol) was then added to the above solution under nitrogen atmosphere. The mixture was stirred vigorously at room temperature for 12 hours, thereafter quenching the reaction by addition of distilled water (20 mL). After allowing the reaction mixture to cool to room temperature, it was extracted with diethyl ether (2 × 30 mL) and the organic layer was washed with water (20 mL) and brine (2 × 20 mL). The separated organic layer was dried over $MgSO_4$ and then evaporated to dryness, leaving the crude product as a colorless oil. Purification by column chromatography (SiO_2; pentane/ethyl acetate, 4:1) afforded *N*-(2-azidoethyl)phthalimide as a white solid (600 mg, 71 %). ^1H NMR (499.93 MHz, $CDCl_3$, 25 °C): δ = 7.88 (m, 2H), 7.74 (m, 2H), 3.90 (t, *J* = 6.1 Hz, 2H), 3.60 (t, *J* = 6.1 Hz, 2H). Data are in accordance with the literature [19]. b) In a heavy-walled smith process vial (15 mL) pyEDOT (95 mg, 0.53 mmol) and *N*-(2-azidoethyl)phthalimide (114 mg, 0.53 mmol) were

dissolved in DMF (2 mL). CuI (20 mg, 0.10 mmol) and Et_3N (1 drop) were added to this solution, and the mixture was sonicated at 35 °C for 30 minutes in an ultrasound bath. The resulting reaction solution was separated between ethyl acetate (20 mL) and water (15 mL), and the organic layer was washed with water (2 × 10 mL) and brine (2 × 10 mL), then dried over $MgSO_4$. After evaporating the solvent in vacuo the residue was purified through column chromatography (SiO_2; pentane/ethyl acetate, 4:1 to 1:1) to afford EDOT derivative **13** as white solid, *mp* 112-116 °C. (153 mg, 73 %). ^{1}H NMR (499.93 MHz, $CDCl_3$, 25 °C), δ = 7.78 (m, 2H, H-23,26), 7.70 (m, 2H, H-24,25), 7.51 (s, 1H, H-12), 6.30 (d, *J* = 3.7 Hz, 1H, H-1/4), 6.25 (d, *J* = 3.7 Hz, 1H, H-1/4), 4.67 (td, *J* = 5.9, 3.0 Hz, 2H, H-16), 4.36 (m, 1H, H-7), 4.20 (dd, *J* = 11.7, 2.1 Hz, 1H, H-8), 4.14 (t, *J* = 5.9 Hz, 2H, H-17), 3.87 (dd, *J* = 11.7, 7.5 Hz, 1H, H-8'), 3.09 (dd, *J* = 15.1, 6.4 Hz, 1H, H-10), 3.04 (dd, *J* = 15.1, 6.5 Hz, 1H, H-10'). ^{13}C NMR (125.7 MHz, $CDCl_3$, 25 °C), δ = 167.7 (C-19,22), 142.9 (C-11), 141.7 (C-2/3), 141.6 (C-2/3), 134.4 (C-24,25), 131.7 (C-20,21), 123.6 (C-23,26), 122.8 (C-12), 99.7 (C-1/4), 99.6 (C-1/4), 72.9 (C-7), 67.5 (C-8), 48.1 (C-16), 37.9 (C-17), 27.5 (C-10). IR (neat, $\tilde{v}$/cm^{-1}): 2956, 1772, 1708, 1484, 1395, 1006, 719. HRMS, calculated for $C_{19}H_{17}N_4O_4S$: *m/z* = 397.09650, found: *m/z* = 397.09632 ([M + H^+]).

NMR spectra

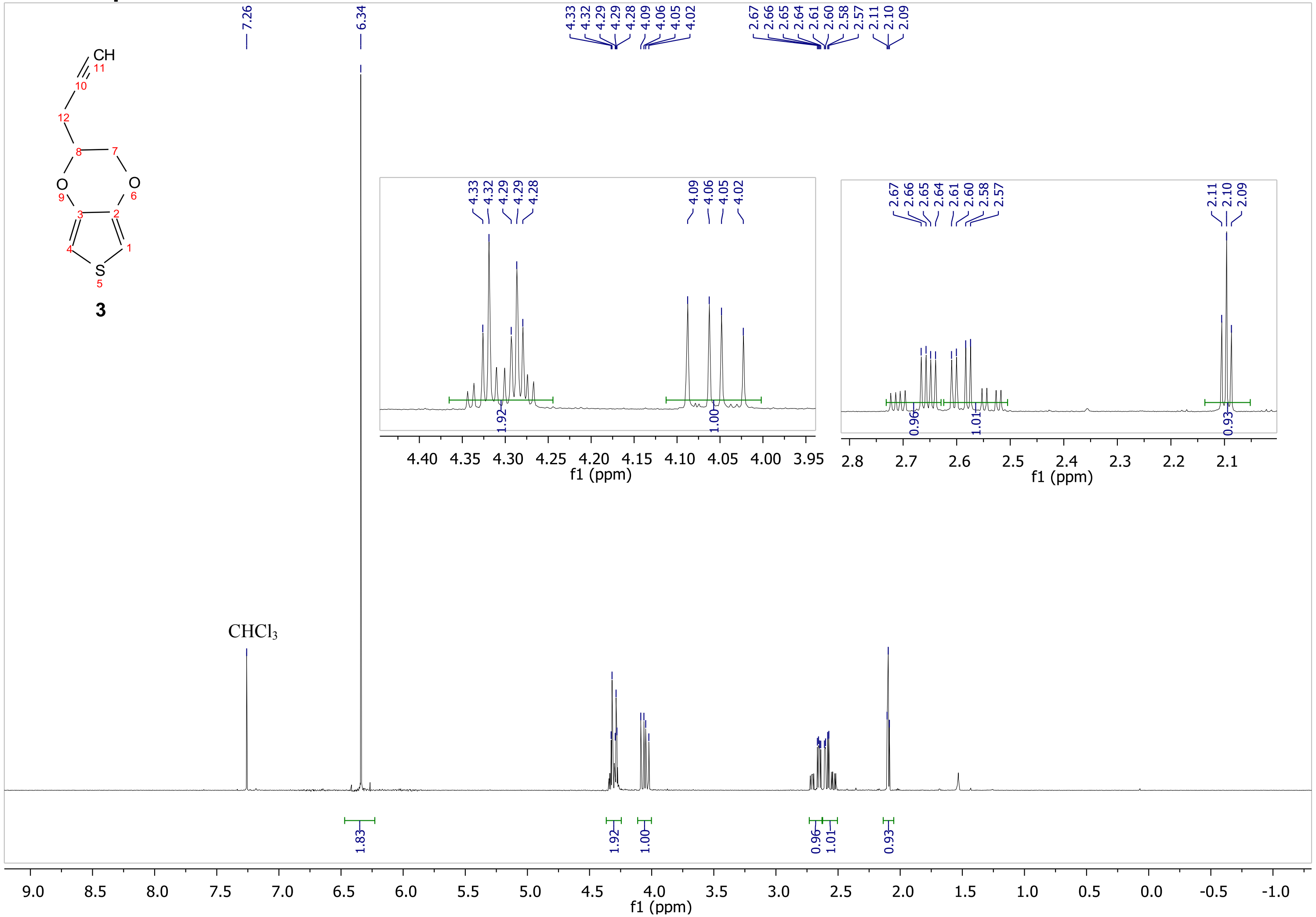

Figure S1: ^{1}H NMR spectrum of **3** (499.93 MHz, $CDCl_3$ solution, 25°C).

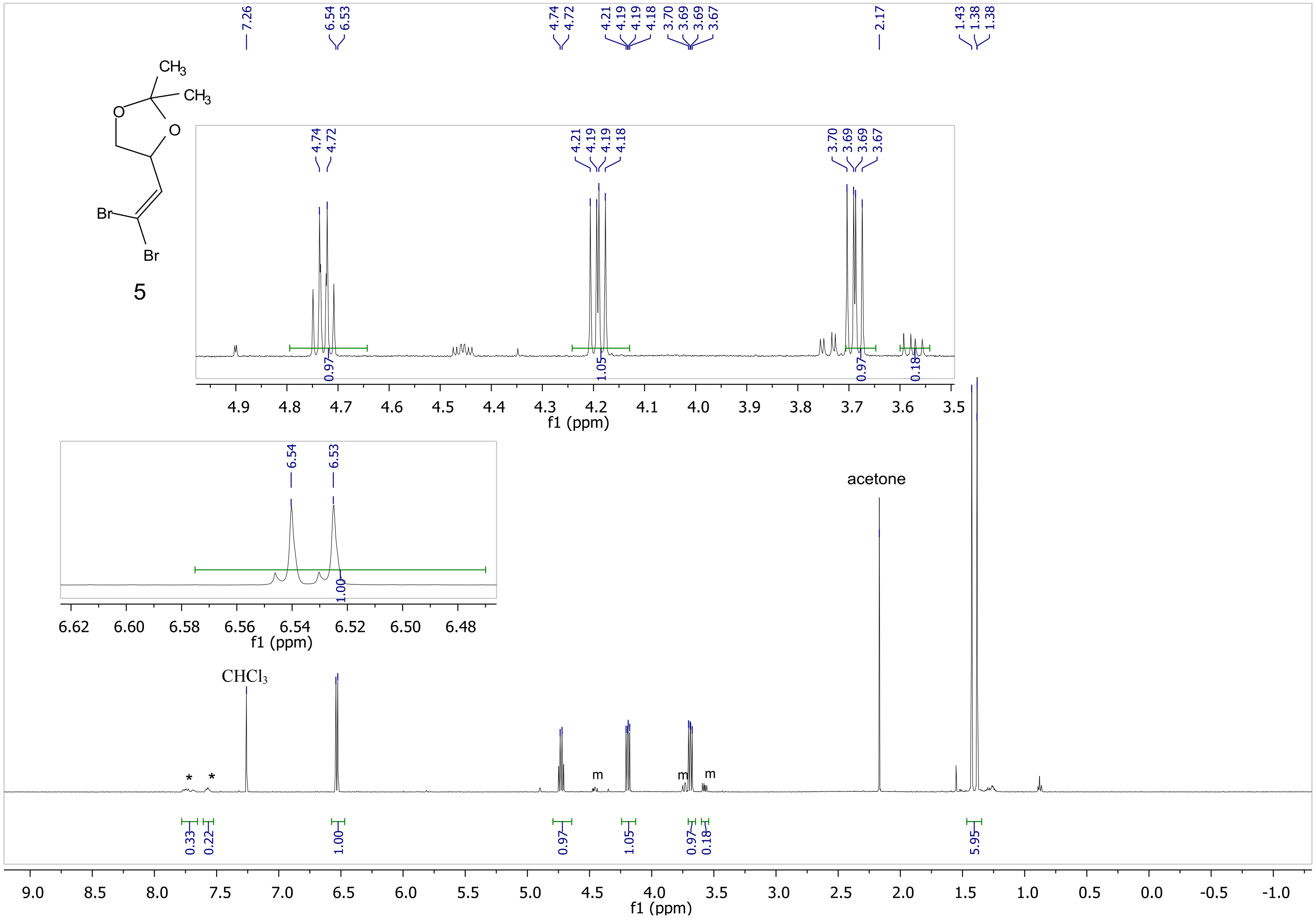

Figure S2: ^{1}H NMR spectrum of **5** (499.93 MHz, $CDCl_3$ solution, 25°C). *: Ph_3PO; ^{m}: impurity

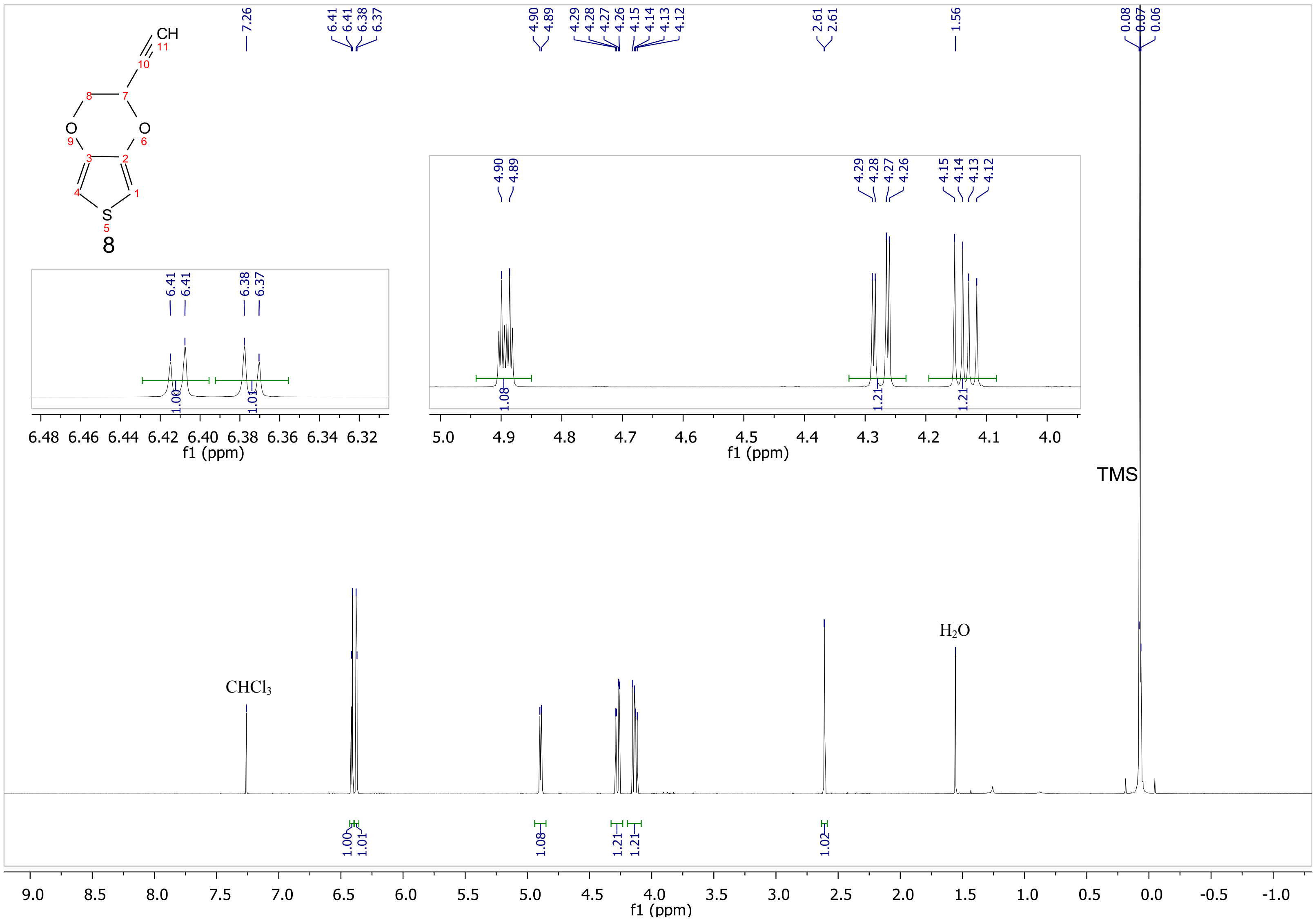

Figure S3: ^{1}H NMR spectrum of **8** (499.93 MHz, $CDCl_3$ solution, 25°C).

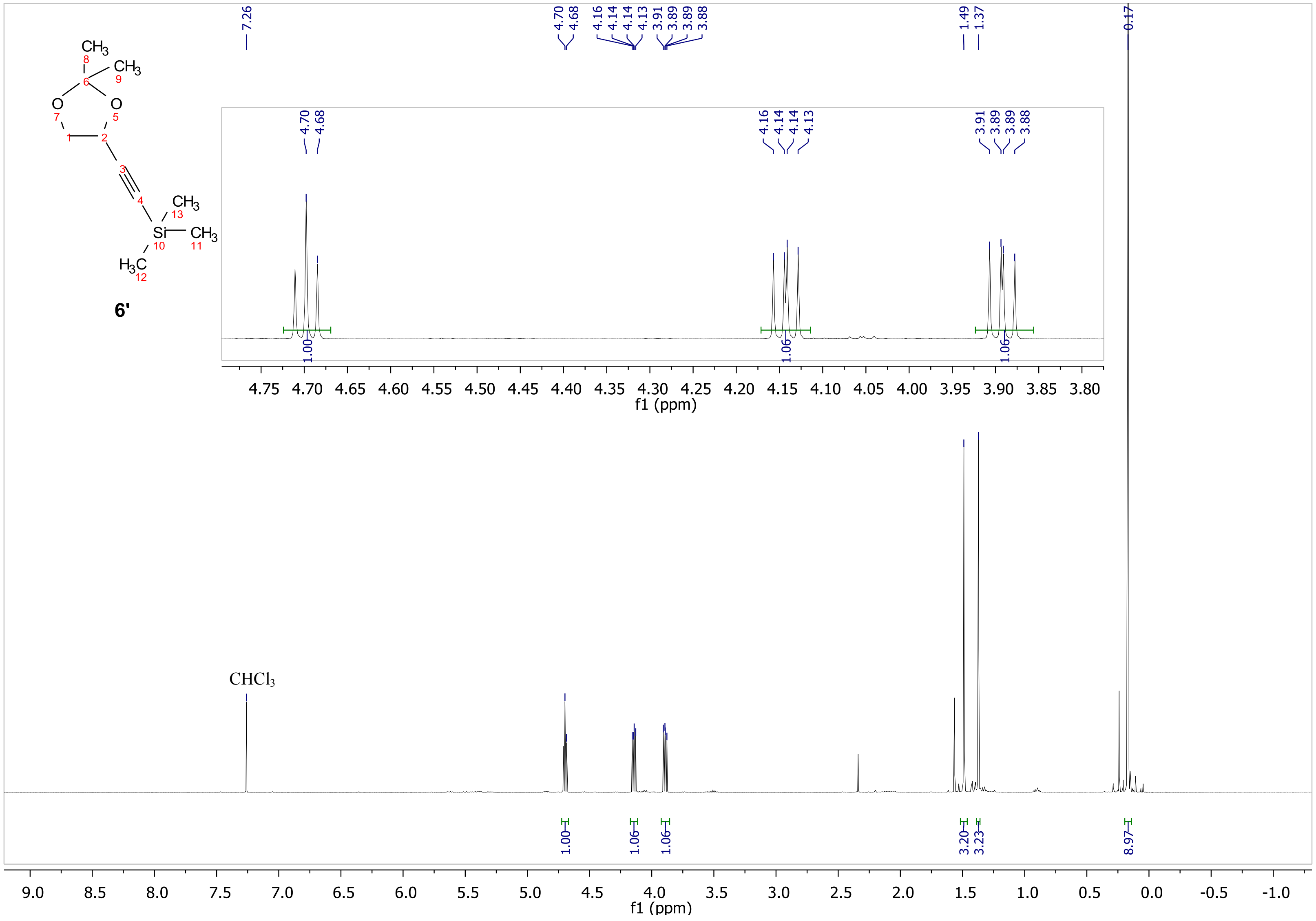

Figure S 4: ^{1}H NMR spectrum of **6’** (499.93 MHz, $CDCl_3$ solution, 25°C).

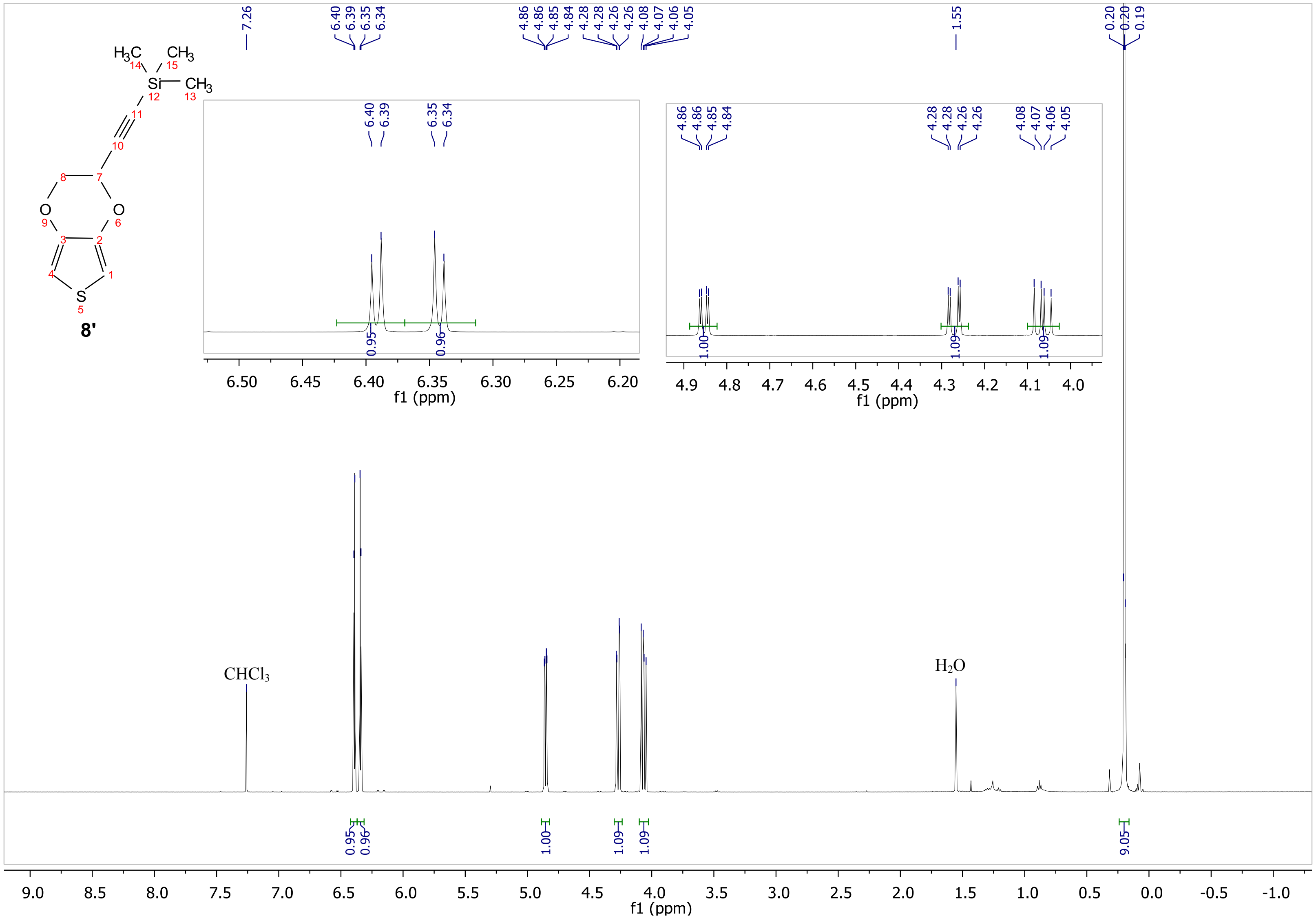

Figure S5: ^{1}H NMR spectrum of **8’** (499.93 MHz, $CDCl_3$ solution, 25°C).

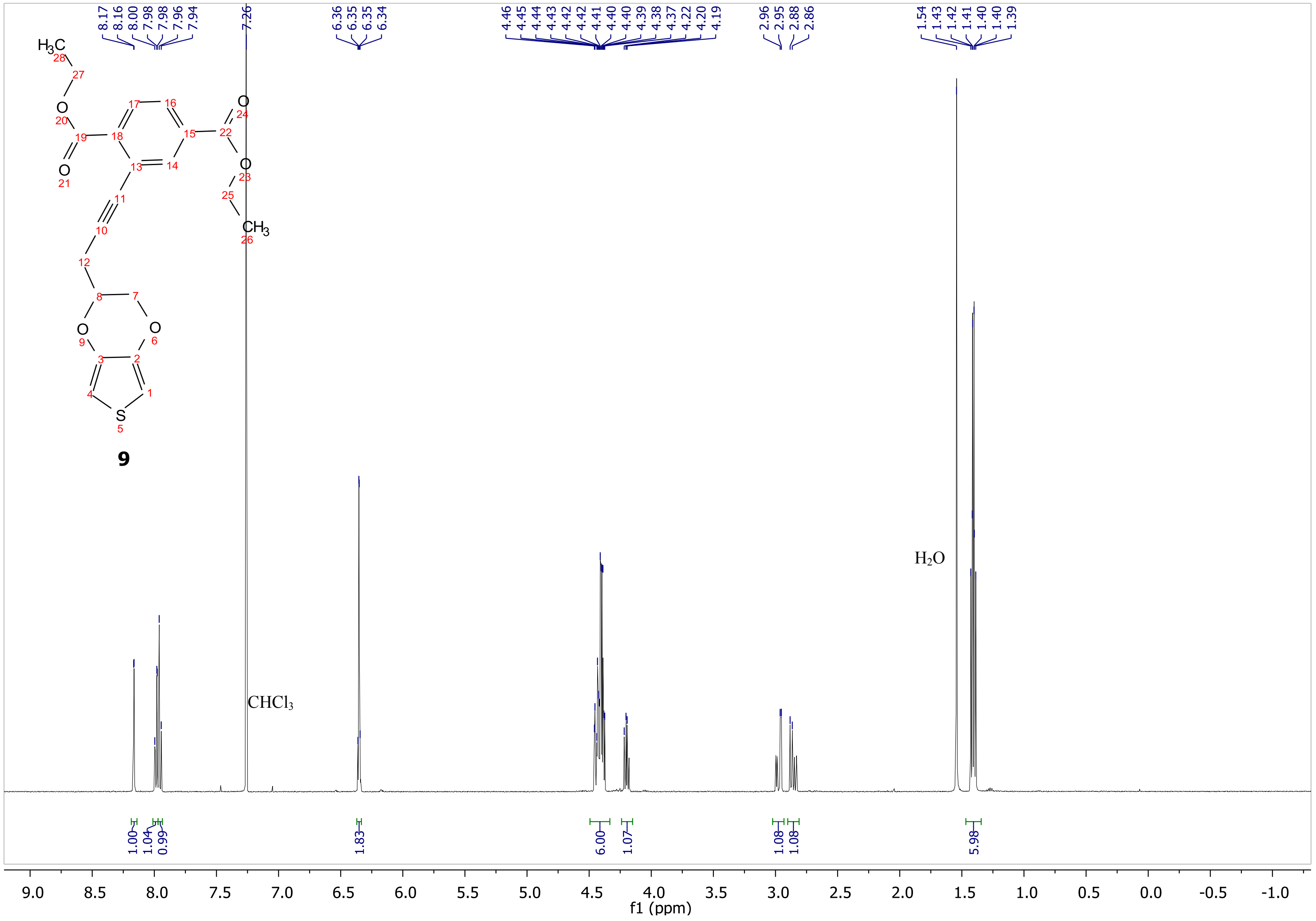

Figure S6: ^{1}H NMR spectrum of **9** (499.93 MHz, $CDCl_3$ solution, 25°C).

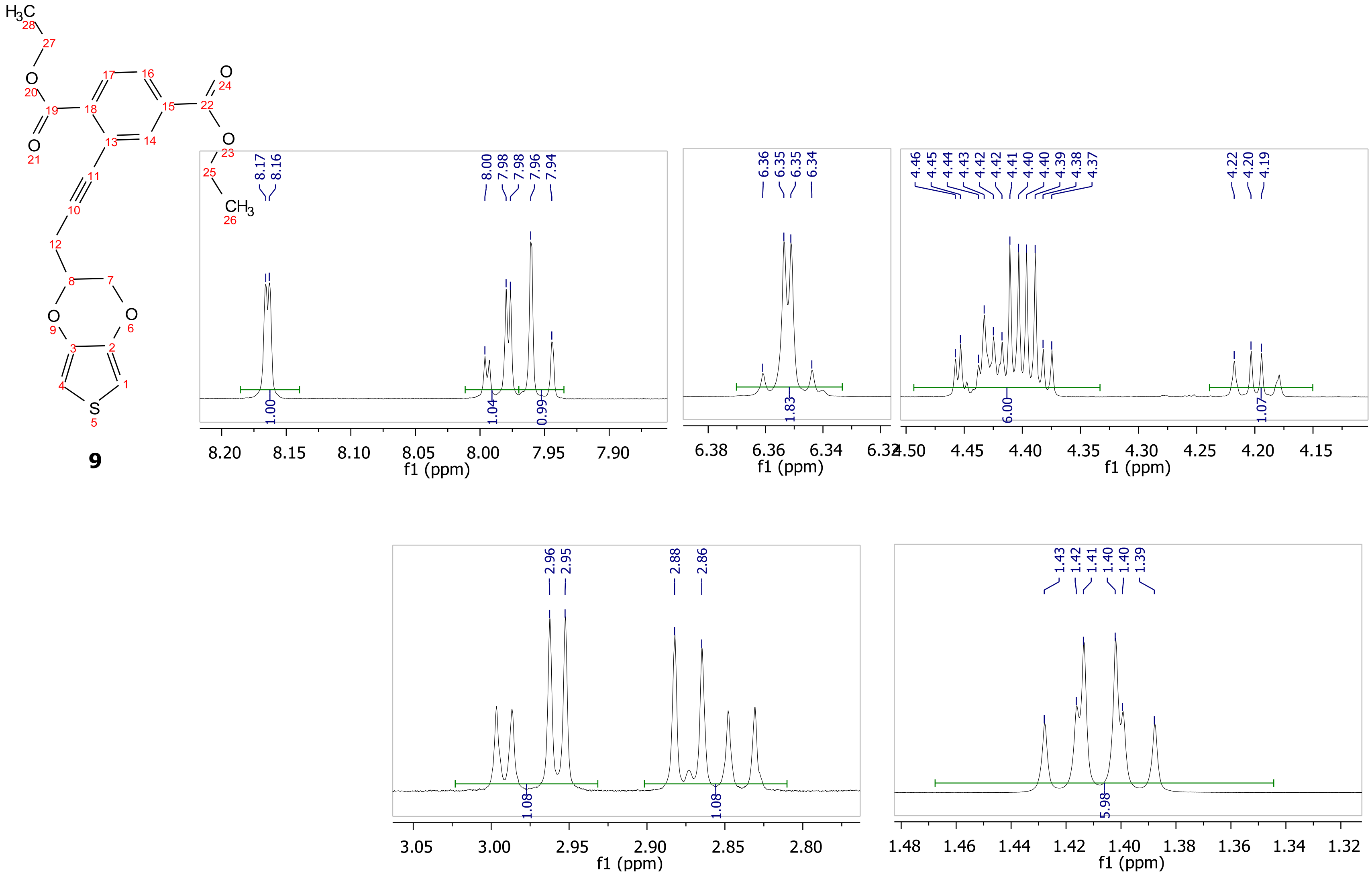

9

Expansion of ^{1}H NMR spectrum of **9** (499.93 MHz, $CDCl_3$ solution, 25°C).

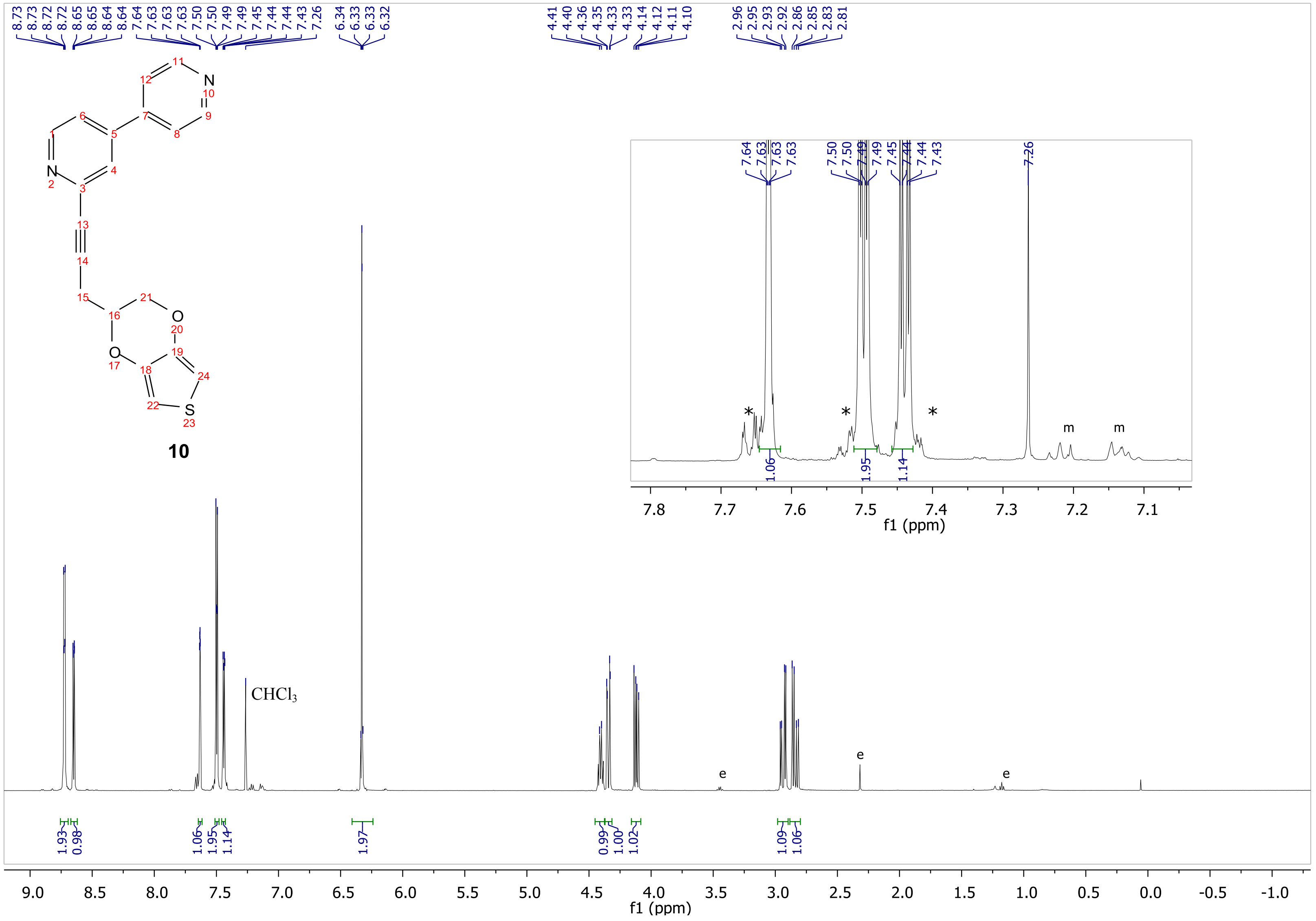

Figure S7: ^{1}H NMR spectrum of **10** (499.93 MHz, $CDCl_3$ solution, 25°C). *: Ph_3PO; ^{m}: Ph_3P; ^{e}: ethyl acetate.

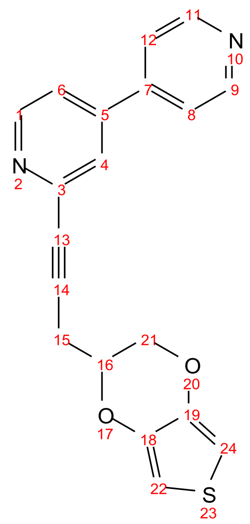

10

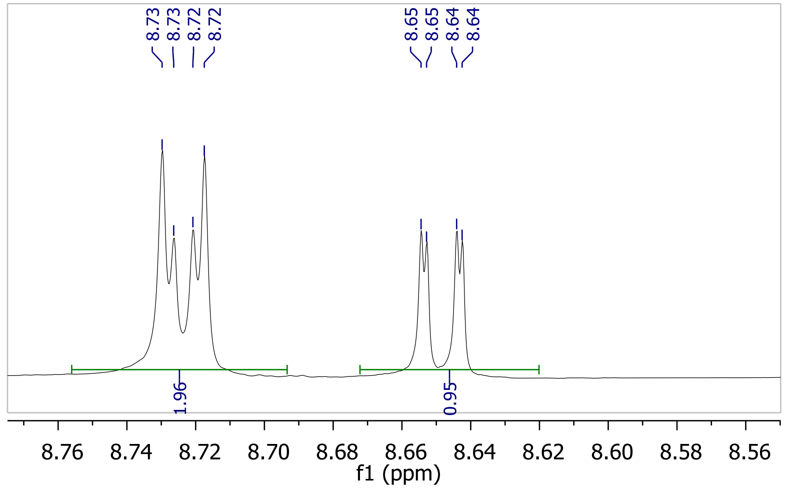

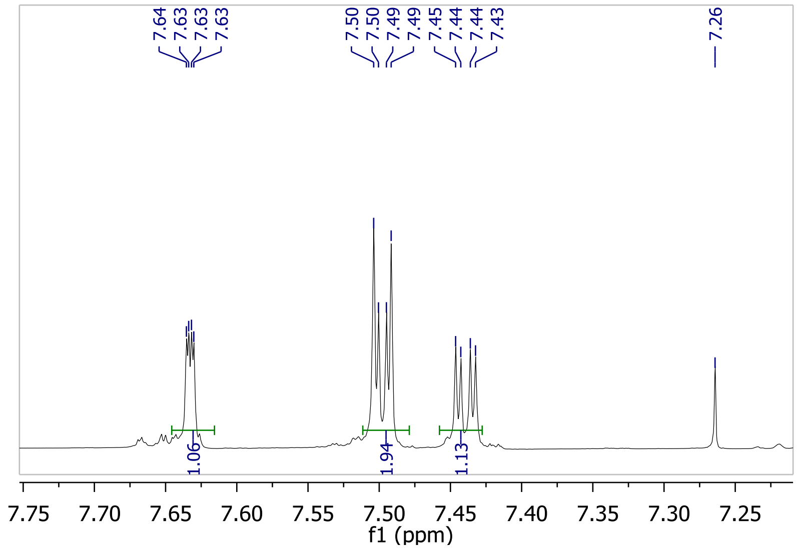

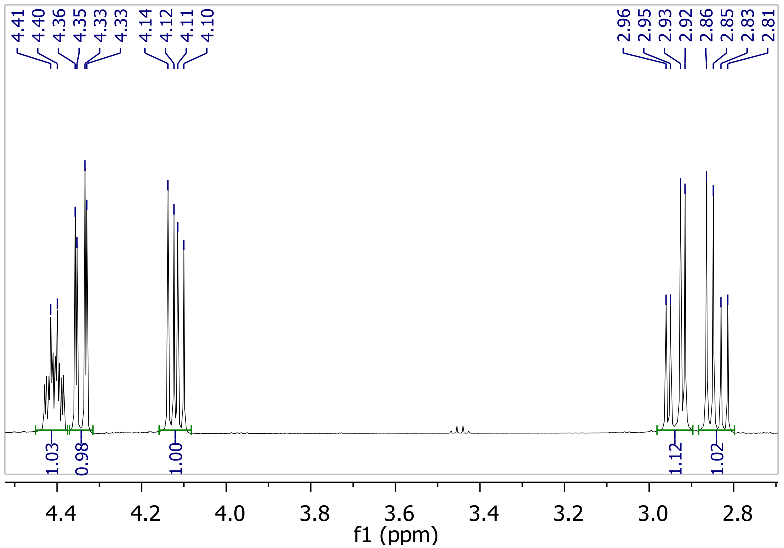

Expansion of ^{1}H NMR spectrum of **10** (499.93 MHz, $CDCl_3$ solution, 25°C).

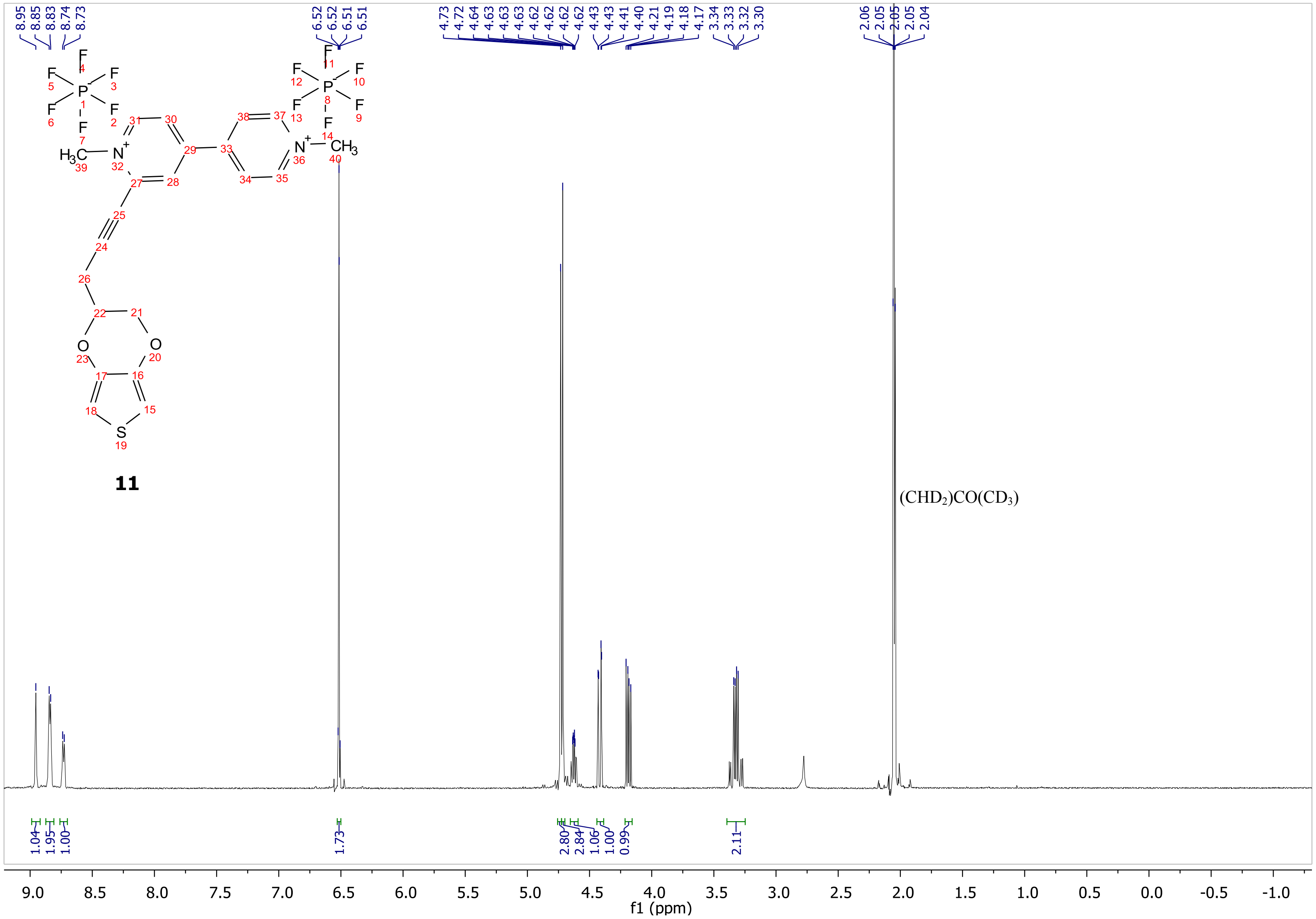

Figure S8: ^{1}H NMR spectrum of **11** (499.93 MHz, $(CD_3)_2CO$ solution, 25°C).

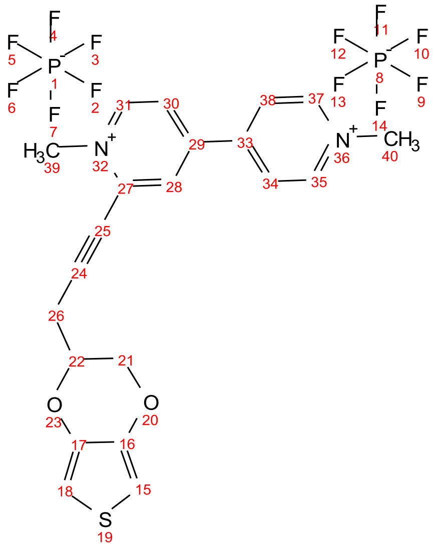

11

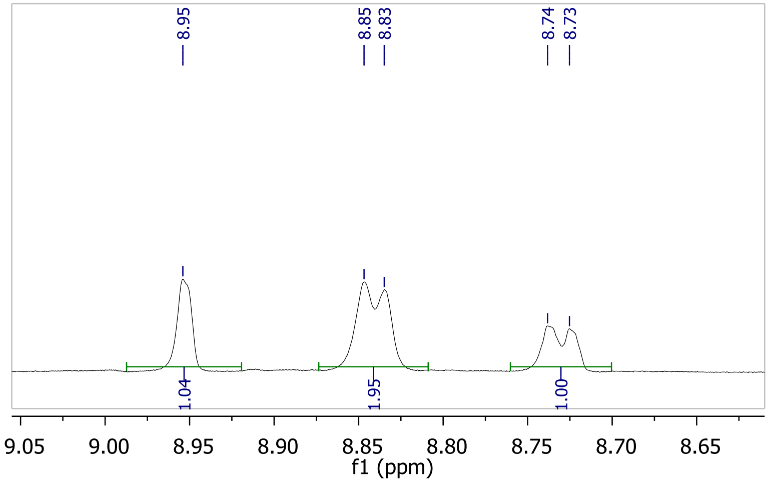

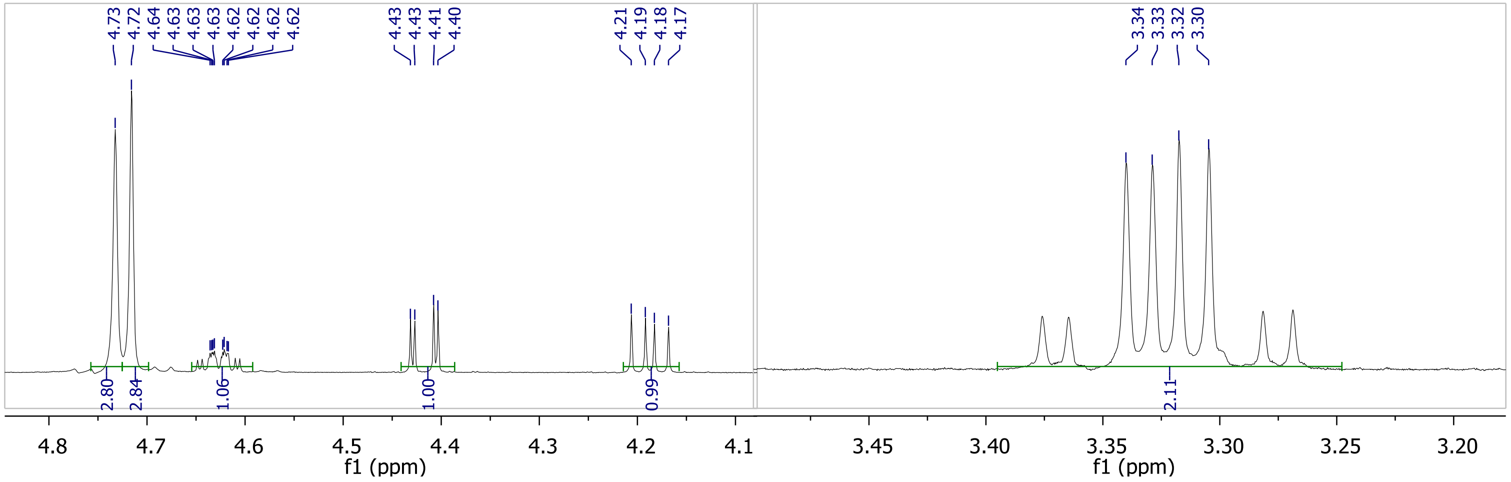

Expansion of ^{1}H NMR spectrum of **11** (499.93 MHz, $(CD_3)_2CO$ solution, 25°C).

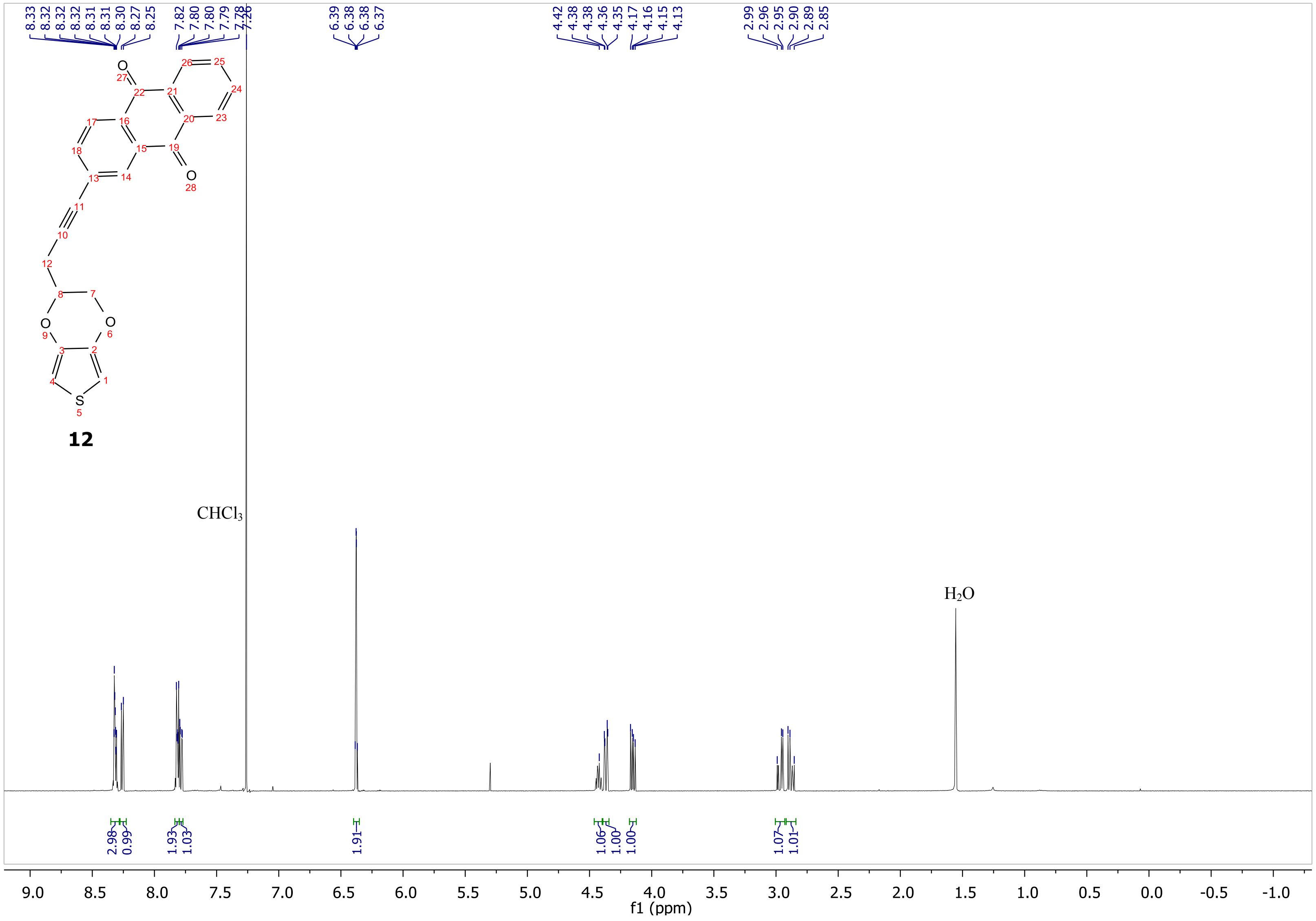

Figure S9: ^{1}H NMR spectrum of **12** (499.93 MHz, $CDCl_3$ solution, 25°C).

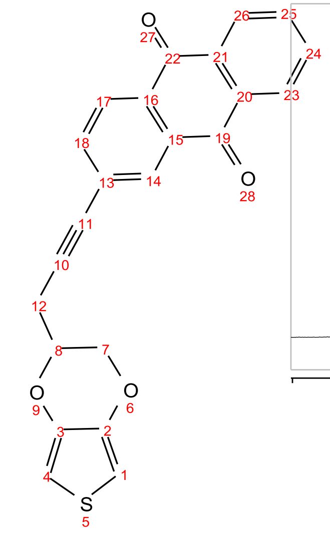

12

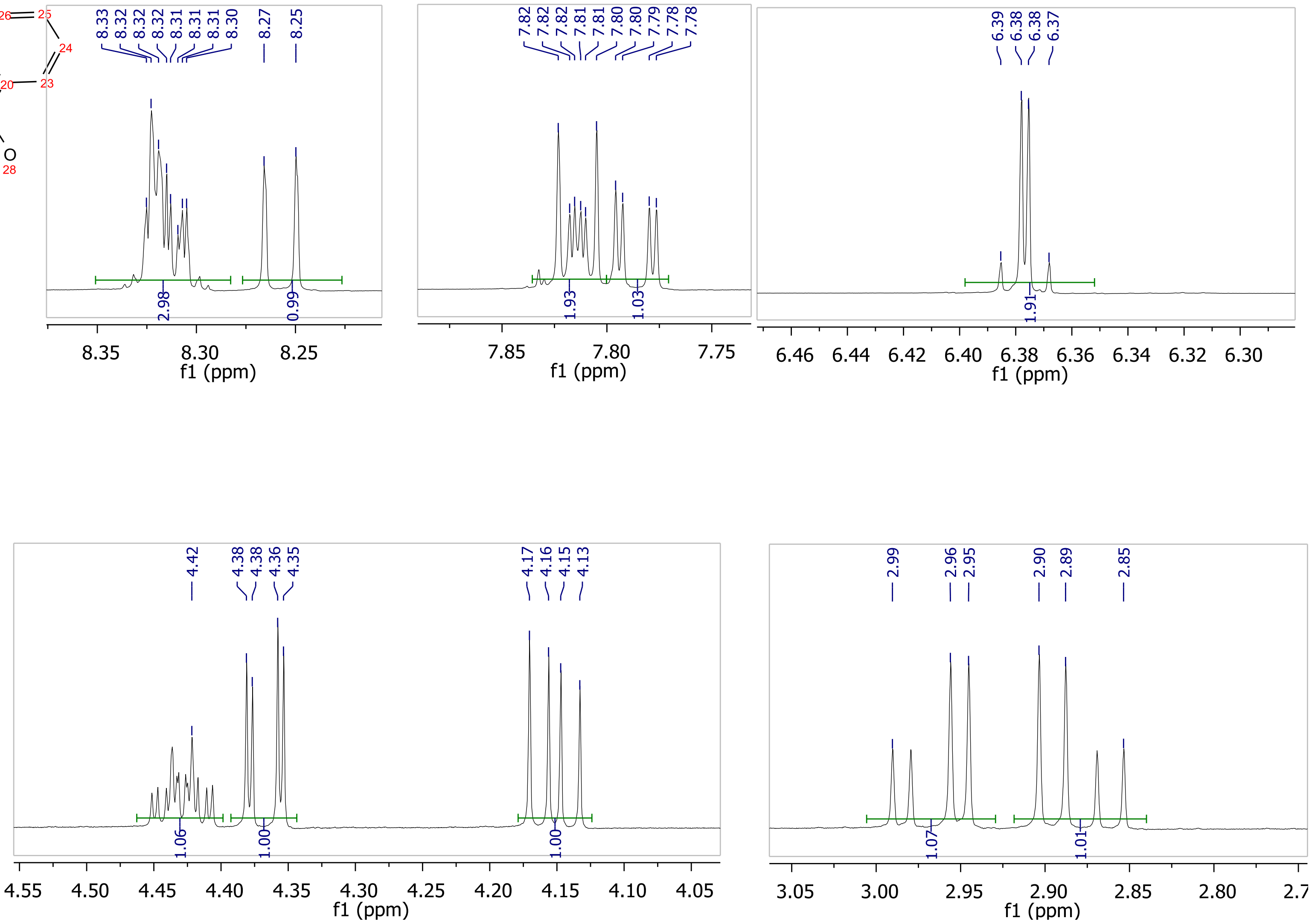

Expansion of ^{1}H NMR spectrum of **12** (499.93 MHz, $CDCl_3$ solution, 25°C).

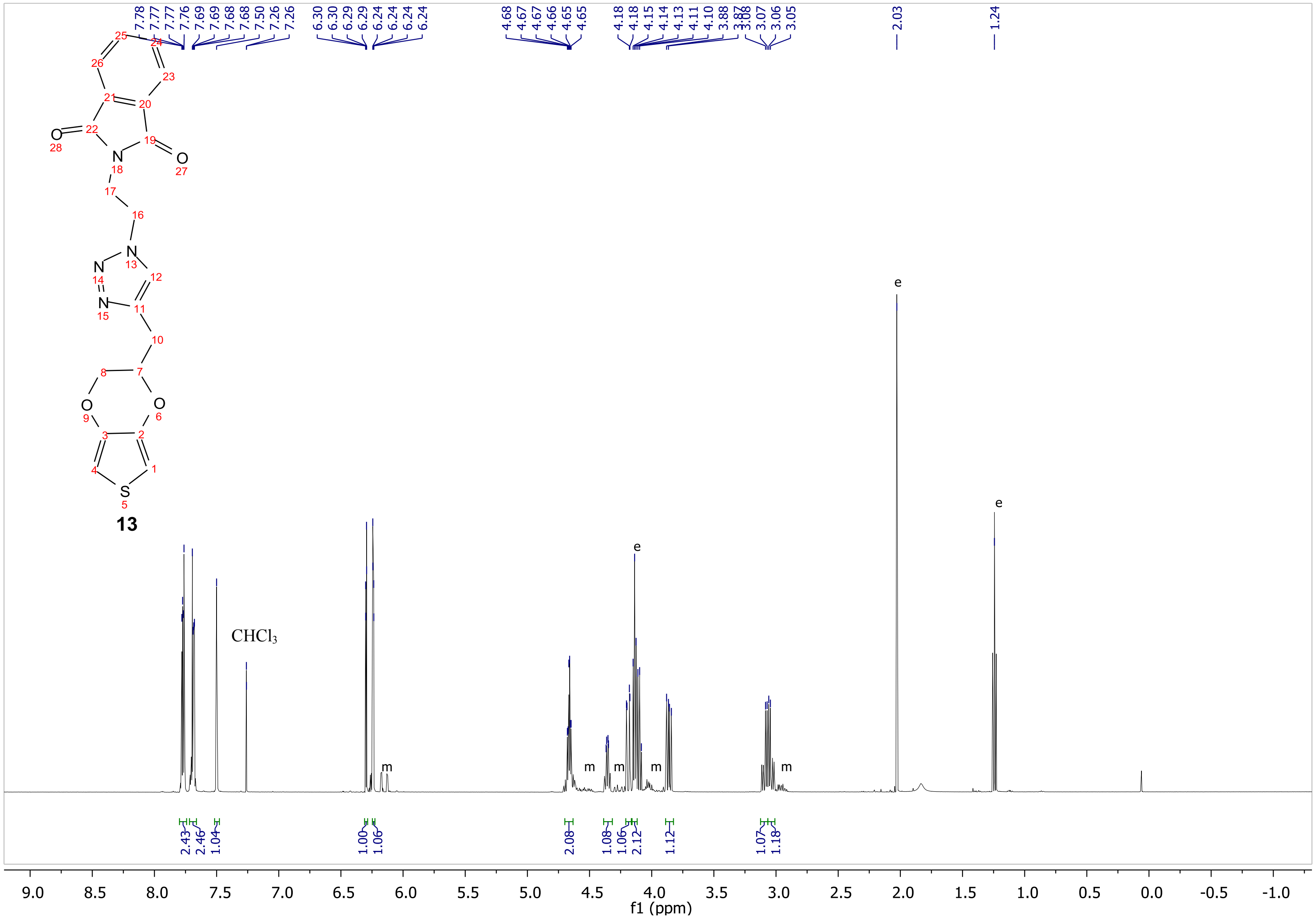

Figure S10: ^{1}H NMR spectrum of **13** (499.93 MHz, $CDCl_3$ solution, 25°C). ^{m}: minor regioisomer; ^{e}: ethyl acetate.

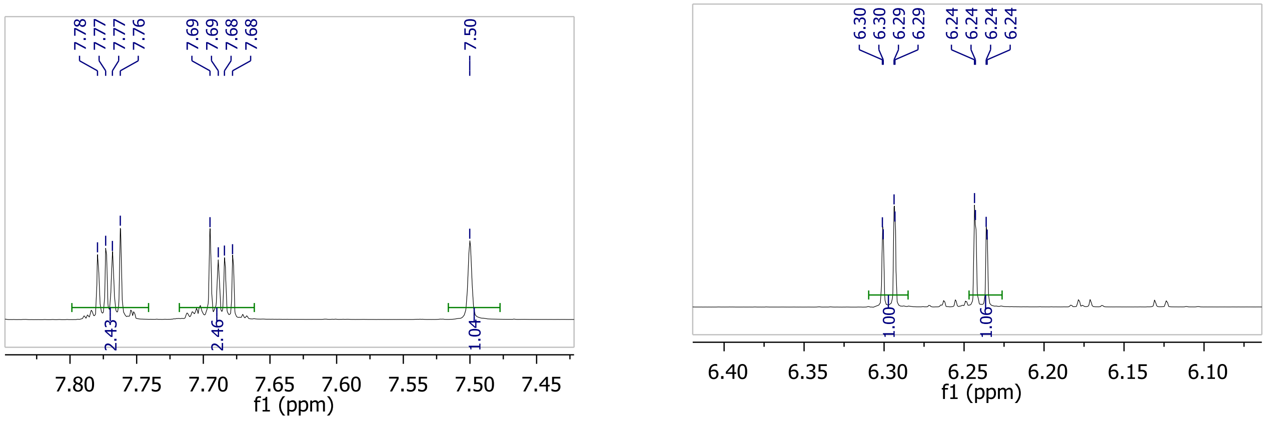

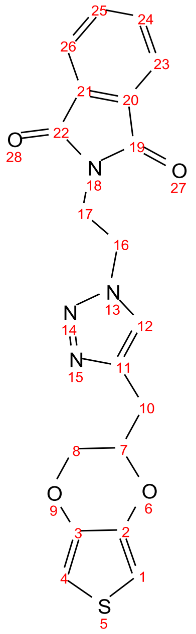

13

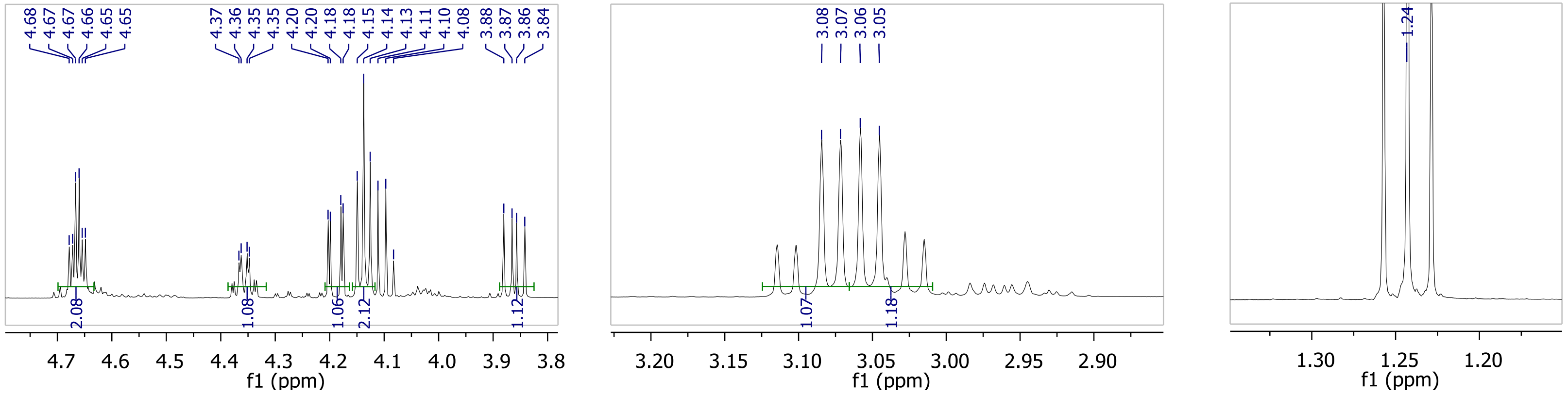

Expansion of ^{1}H NMR spectrum of **13** (499.93 MHz, $CDCl_3$ solution, 25°C).

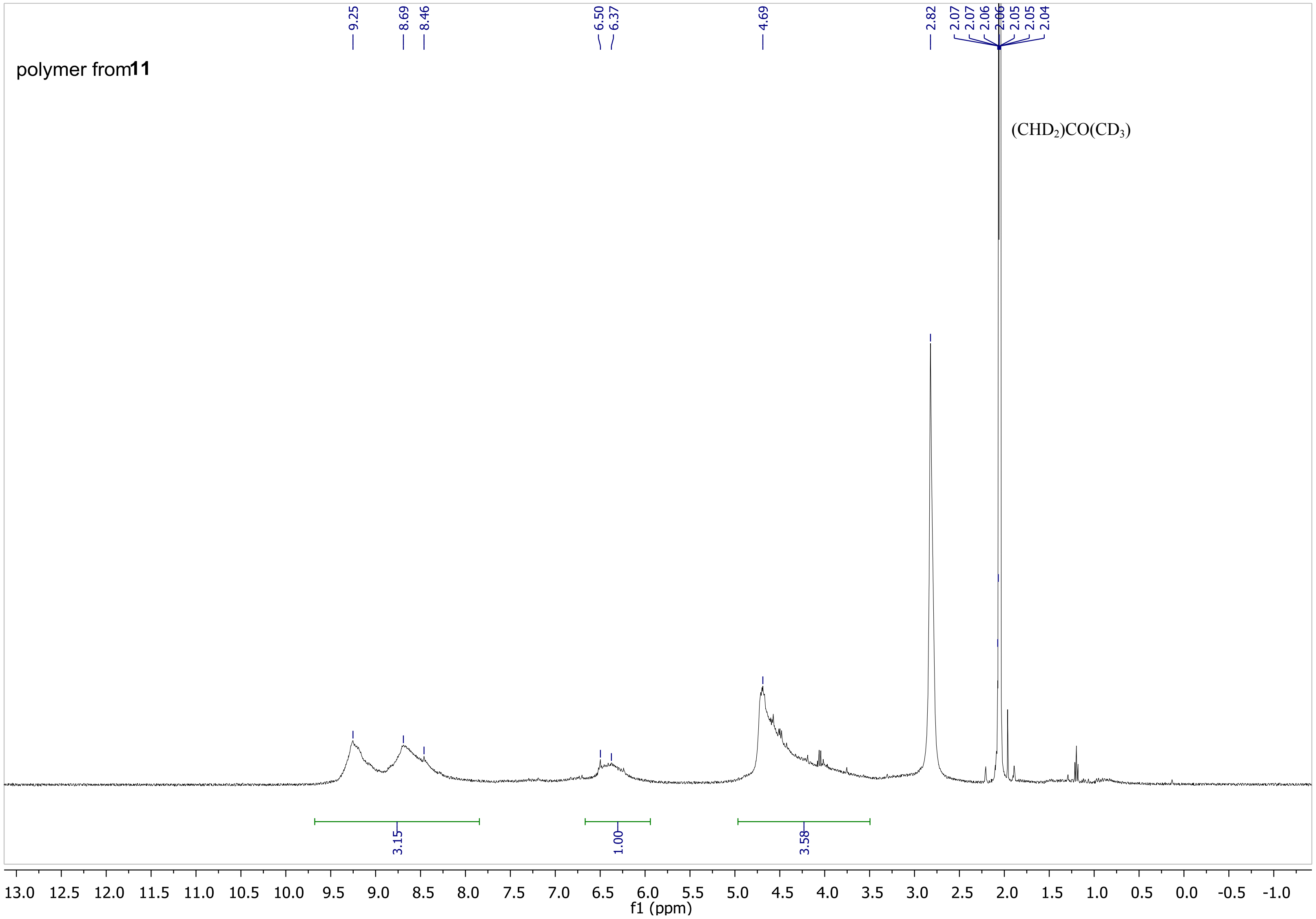

Figure S11: ^{1}H NMR spectrum of polymer from **11** (399.97 MHz, $(CD_3)_2CO$ solution, 25°C).

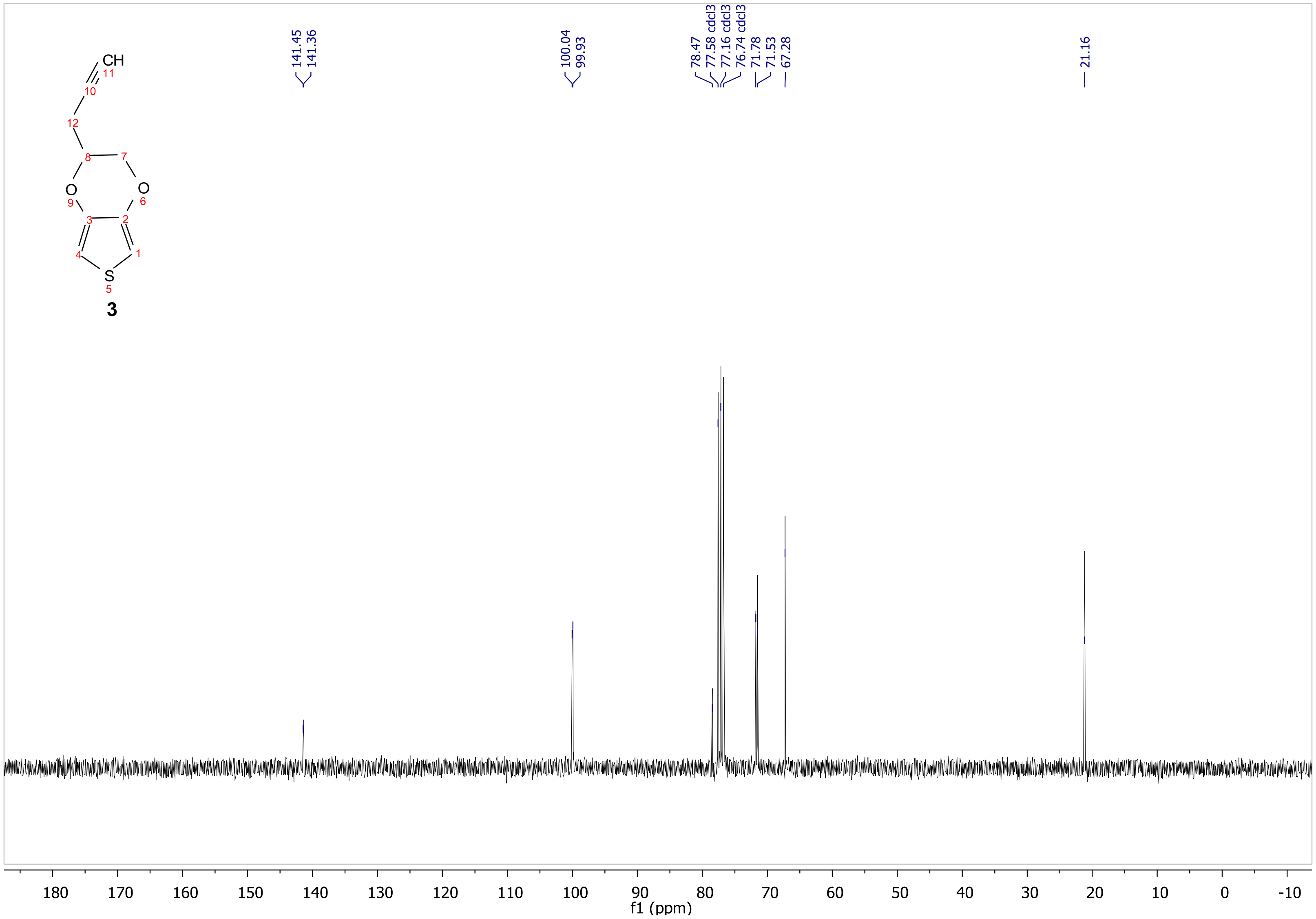

Figure S12: ^{13}C NMR spectrum of **3** (125.7 MHz, $CDCl_3$ solution, 25°C).

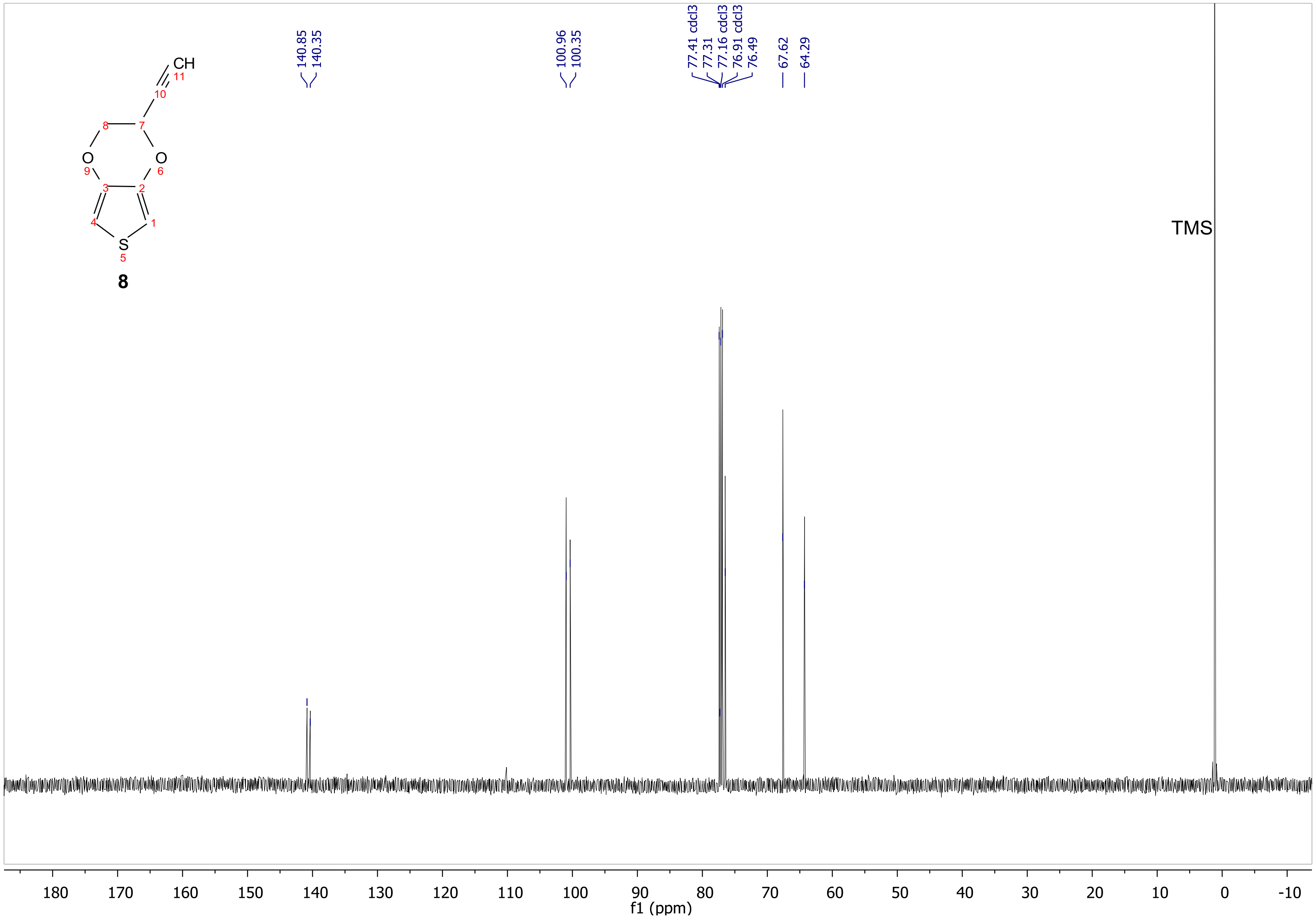

Figure S13: ^{13}C NMR spectrum of **8** (125.7 MHz, $CDCl_3$ solution, 25°C).

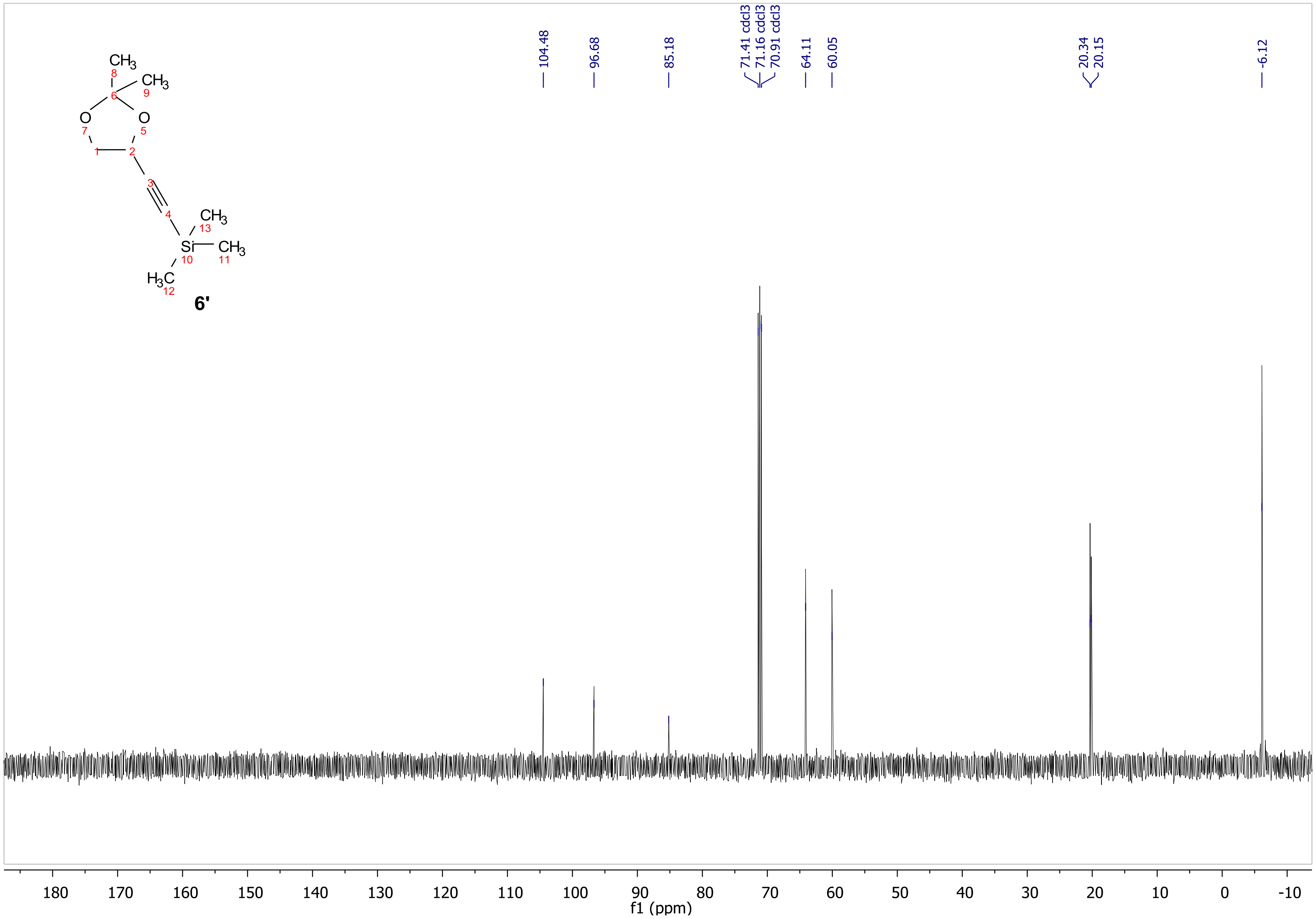

Figure S14: ^{13}C NMR spectrum of **6’** (125.7 MHz, $CDCl_3$ solution, 25°C).

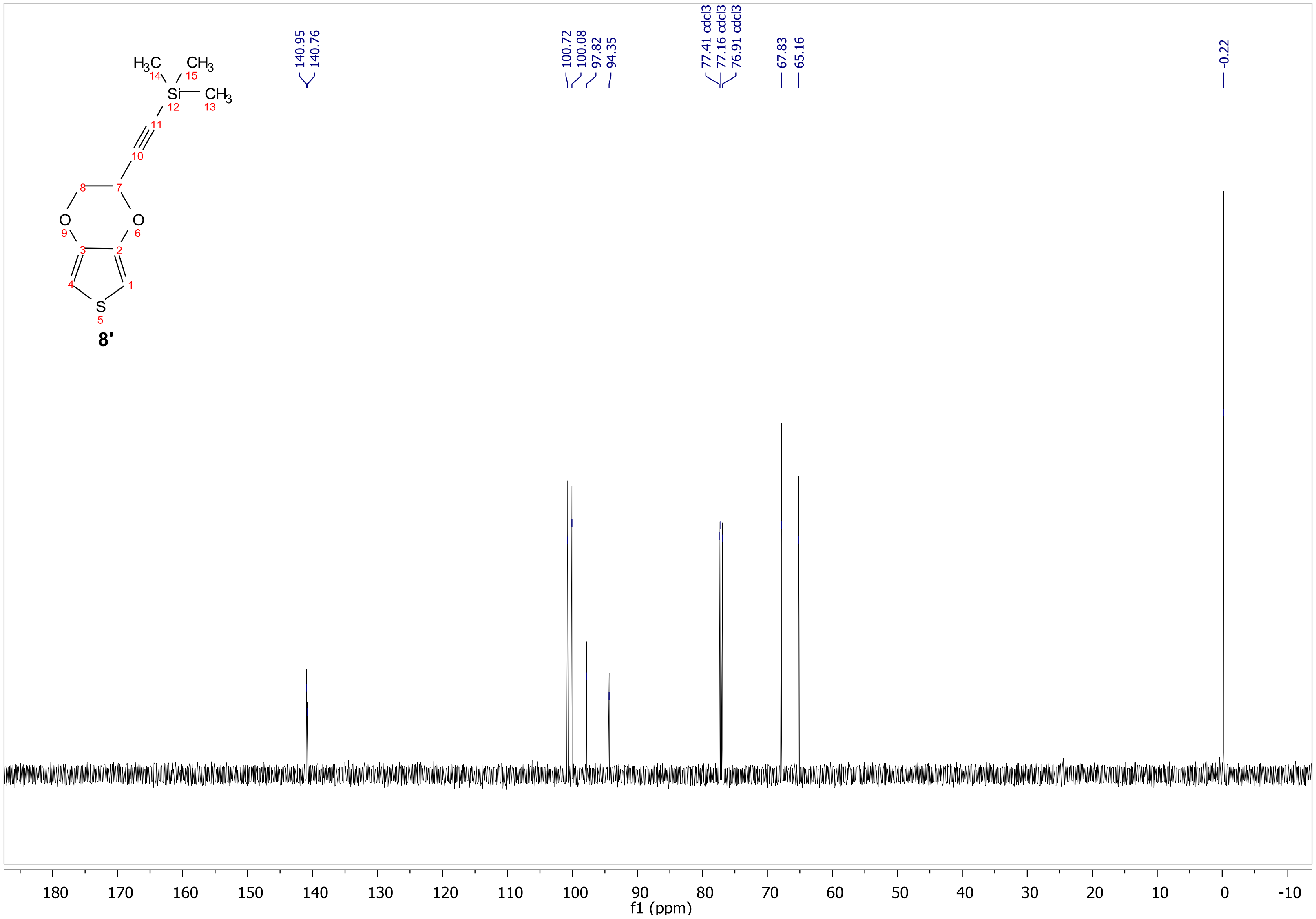

Figure S15: ^{13}C NMR spectrum of **8’** (125.7 MHz, $CDCl_3$ solution, 25°C).

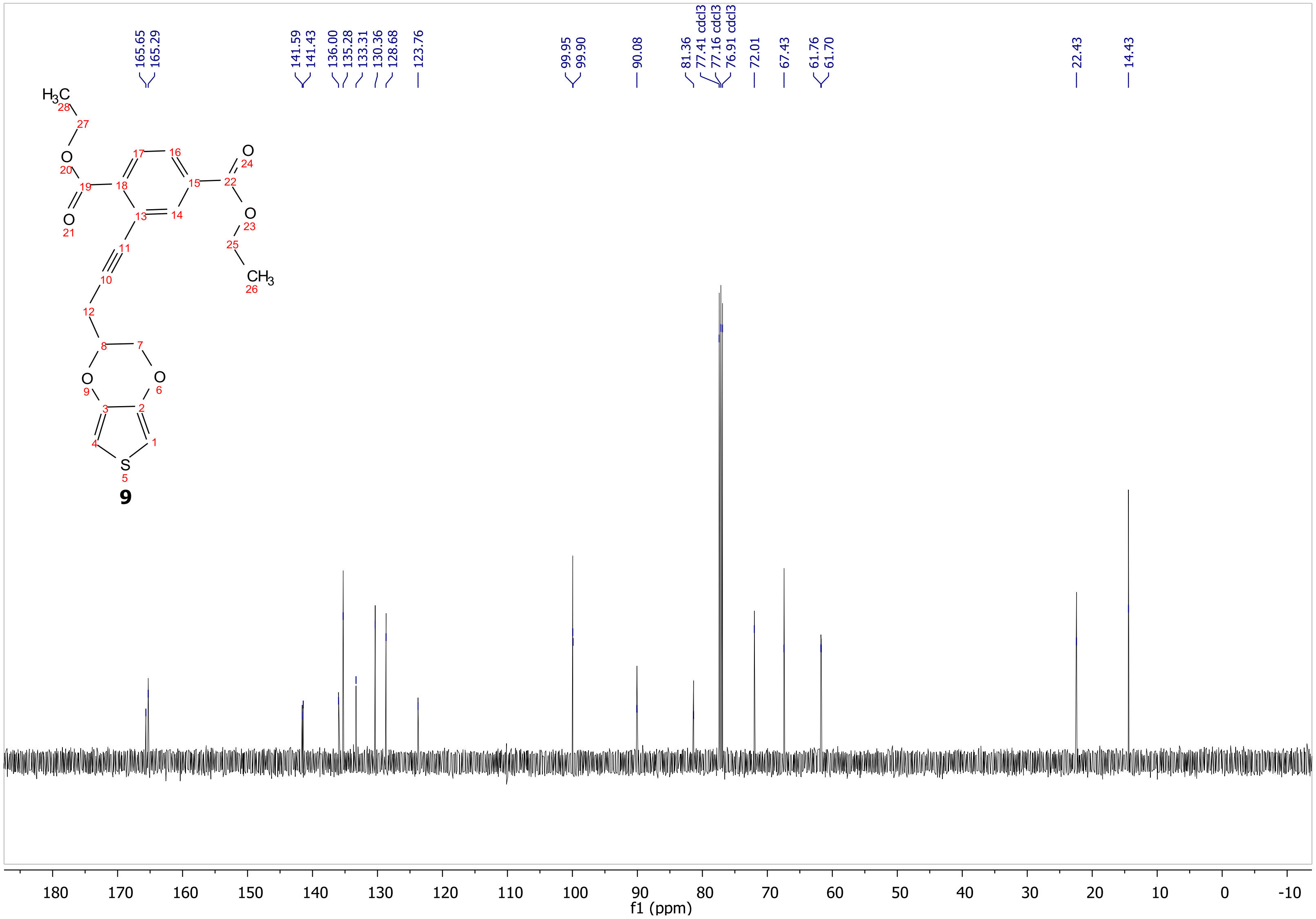

Figure S16: ^{13}C NMR spectrum of **9** (125.7 MHz, $CDCl_3$ solution, 25°C).

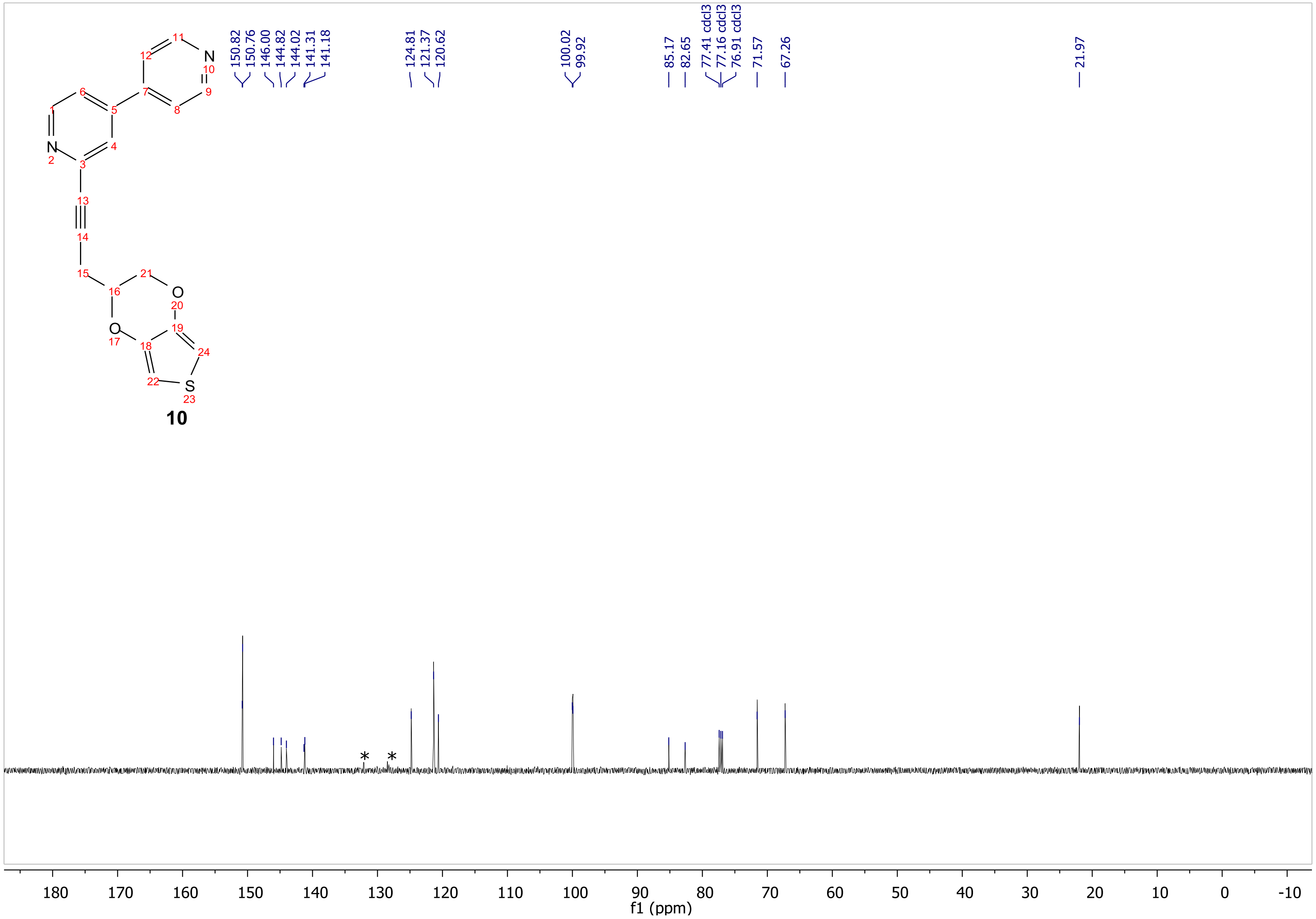

Figure S17: ^{13}C NMR spectrum of **10** (125.7 MHz, $CDCl_3$ solution, 25°C). *: Ph_3PO.

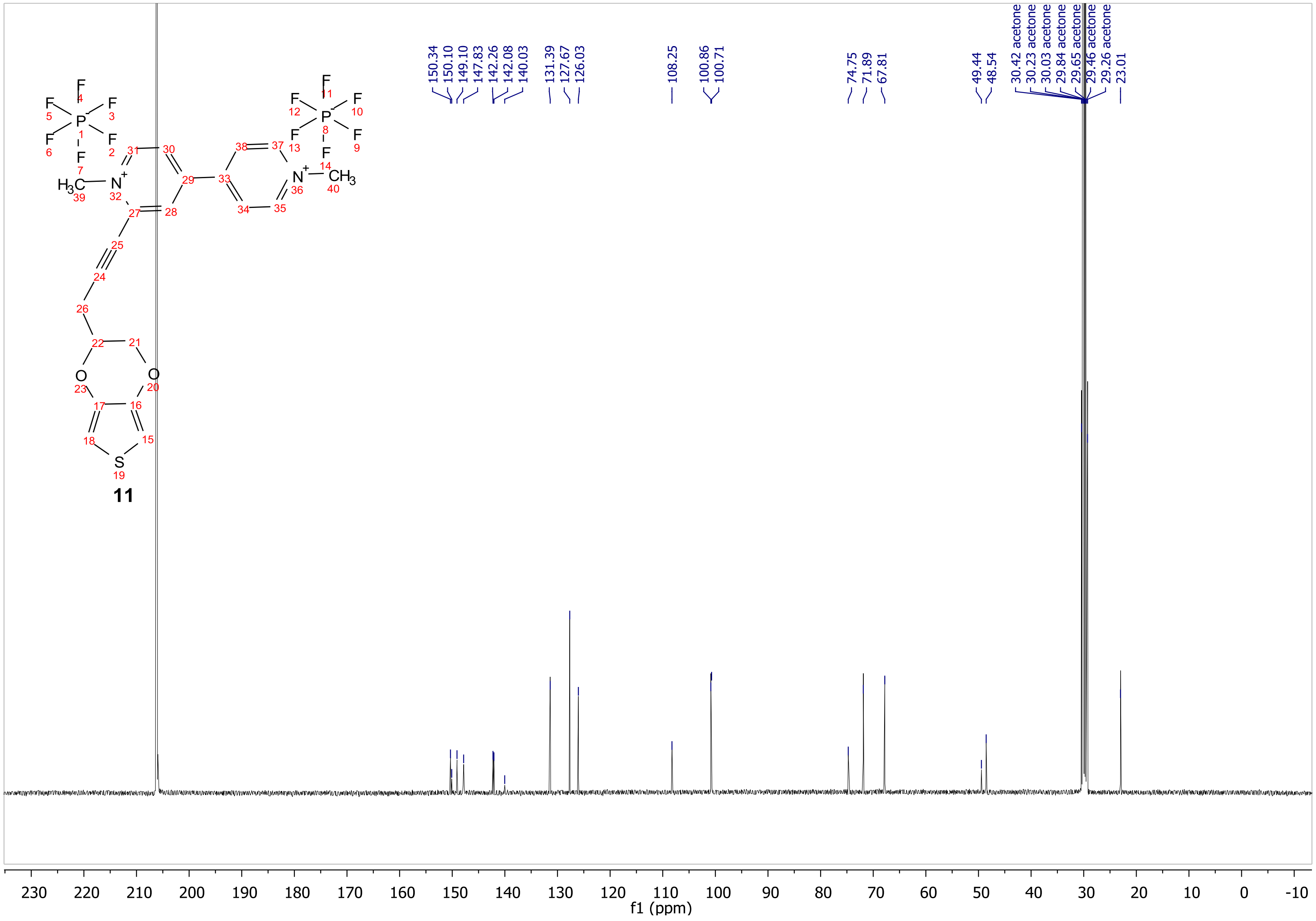

Figure S18: ^{13}C NMR spectrum of **11** (100.58 MHz, $(CD_3)_2CO$ solution, 25°C).

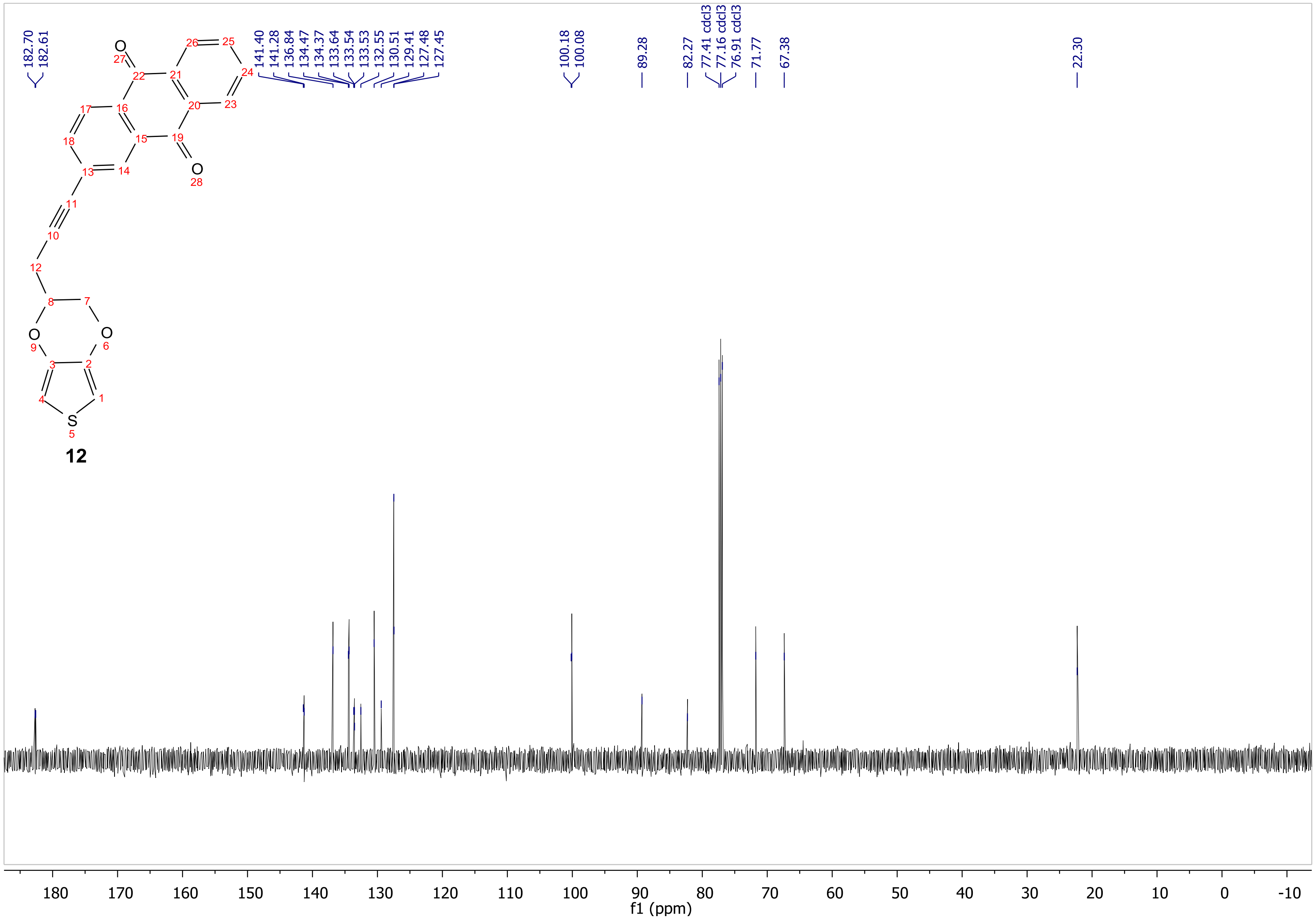

Figure S19: ^{13}C NMR spectrum of **12** (125.7 MHz, $CDCl_3$ solution, 25°C).

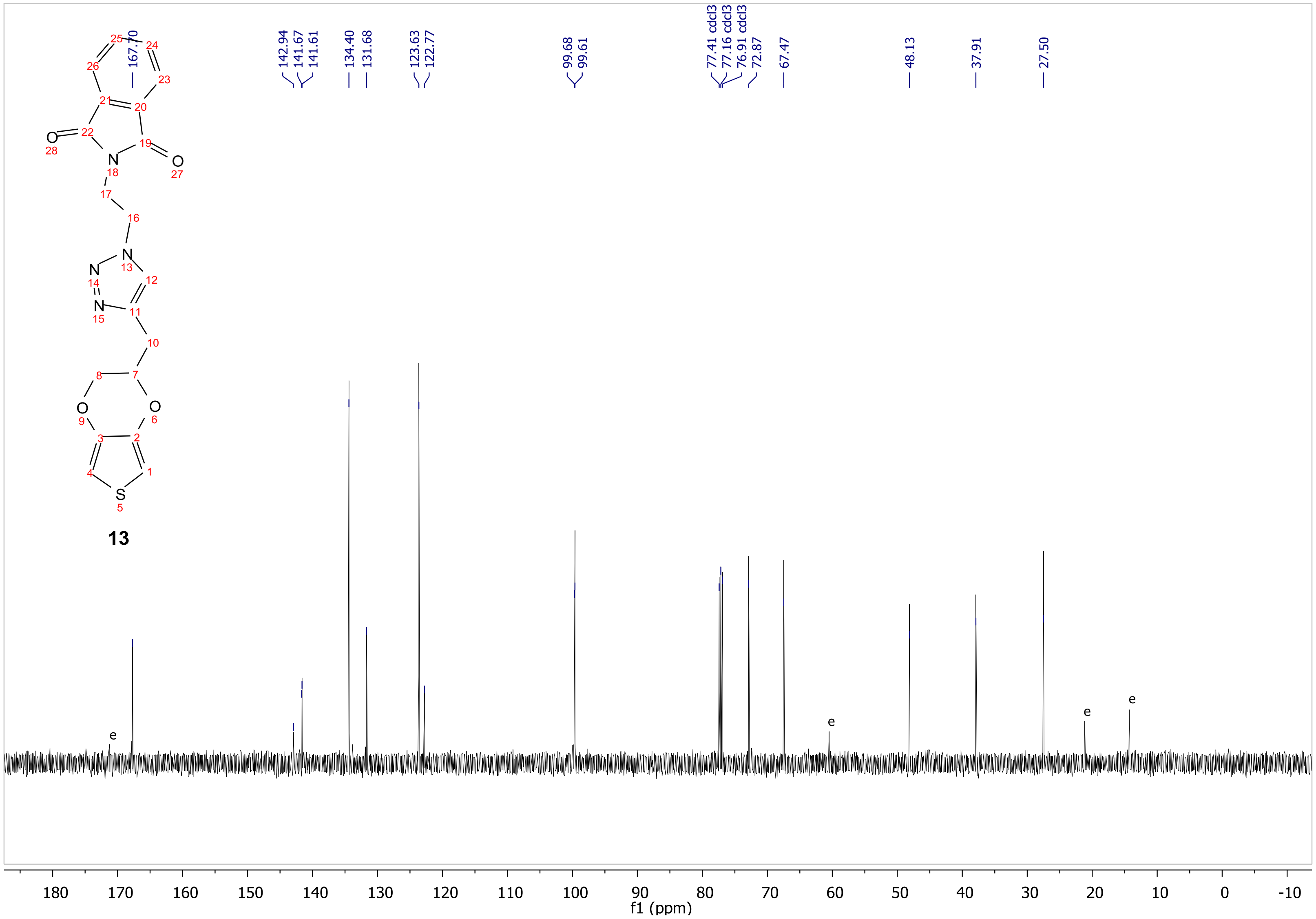

Figure S20: ^{13}C NMR spectrum of **13** (125.7 MHz, $CDCl_3$ solution, 25°C). e: ethyl acetate.

IR spectra

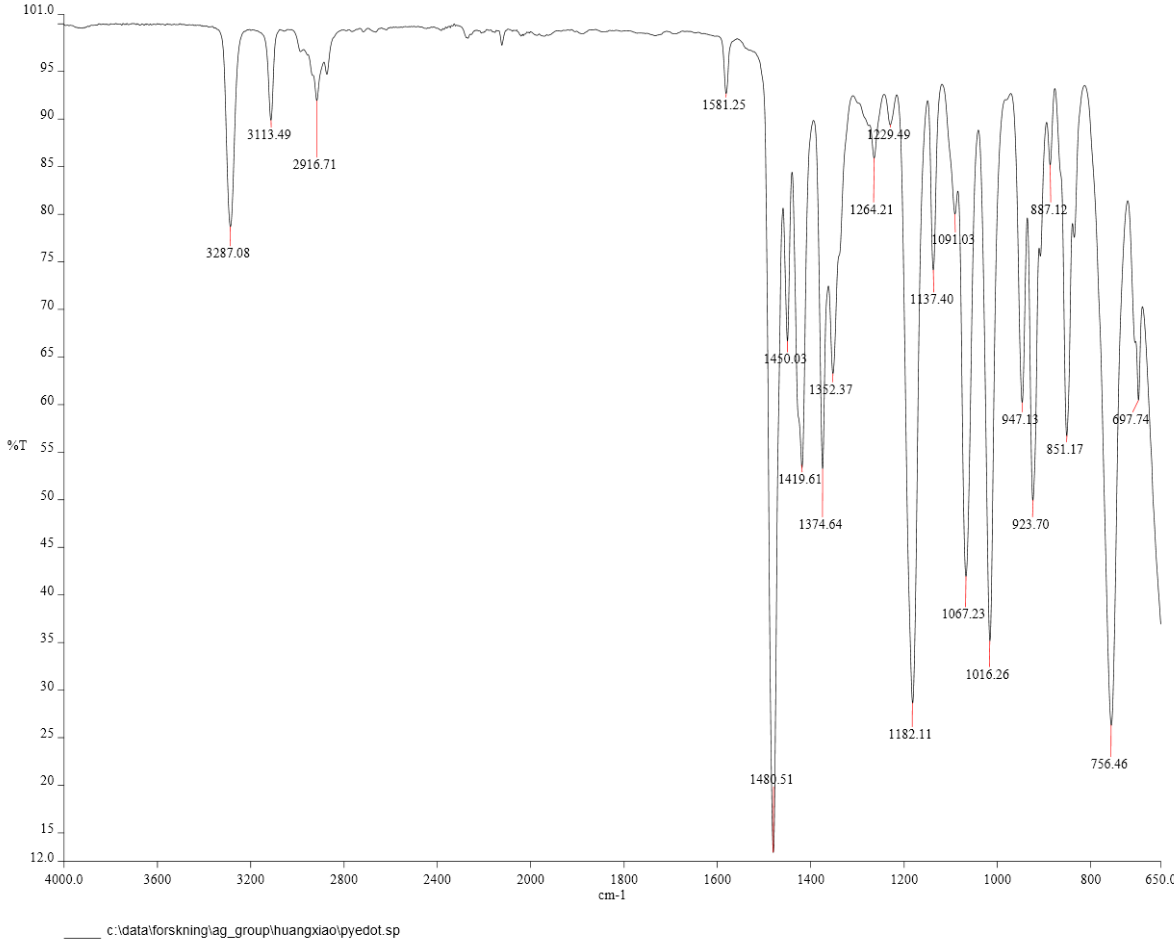

Figure S21: IR spectrum of **3**.

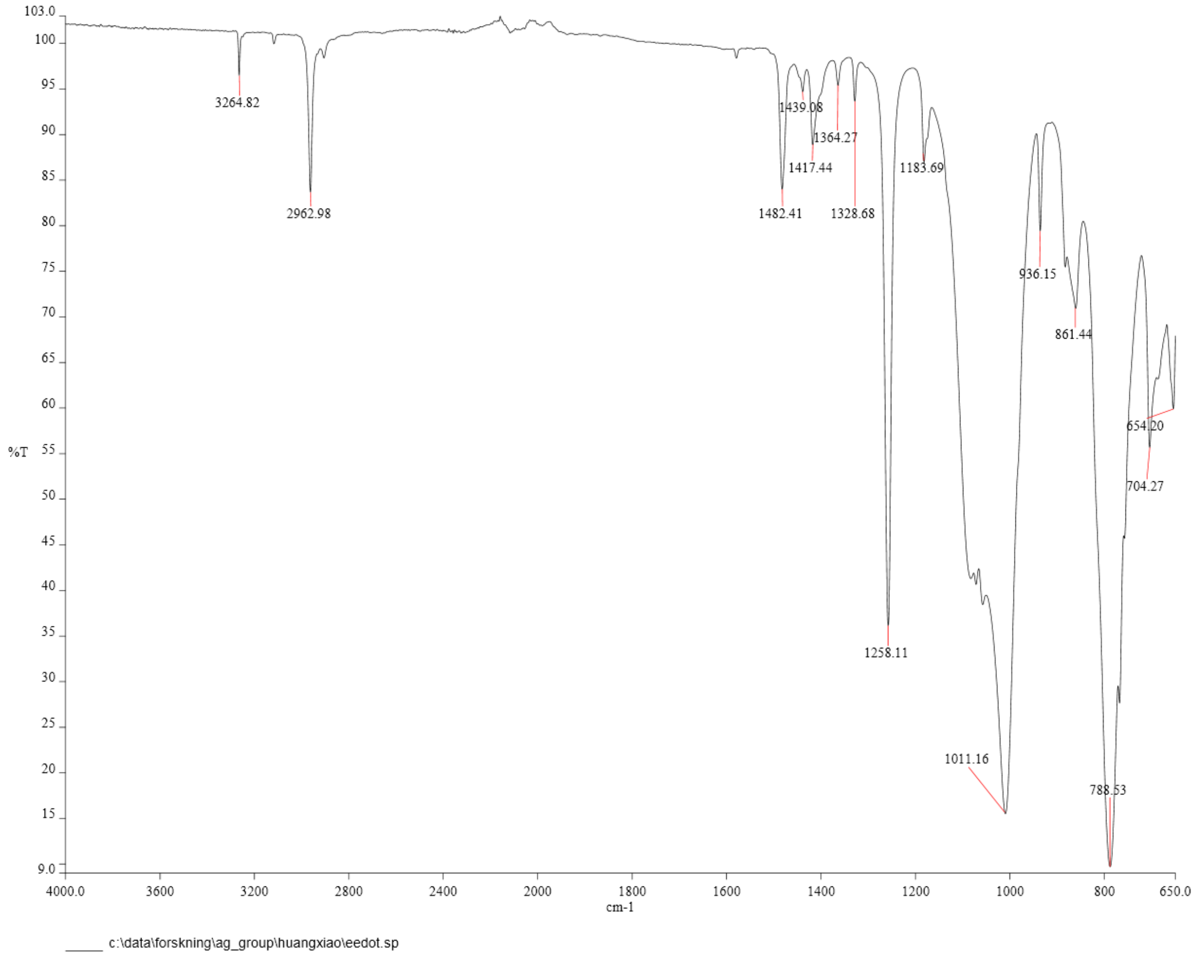

Figure S22: IR spectrum of **8**.

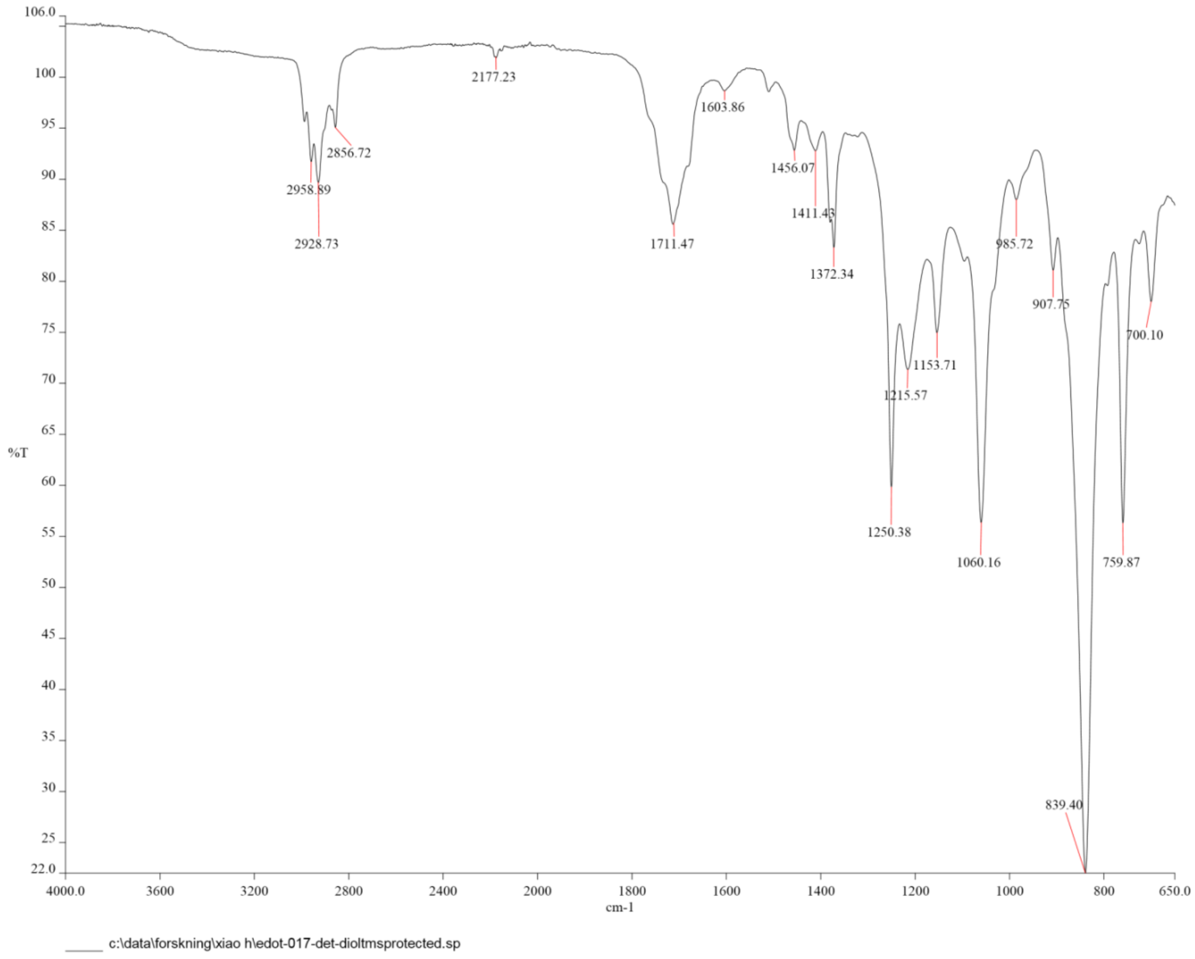

Figure S23: IR spectrum of **6'**.

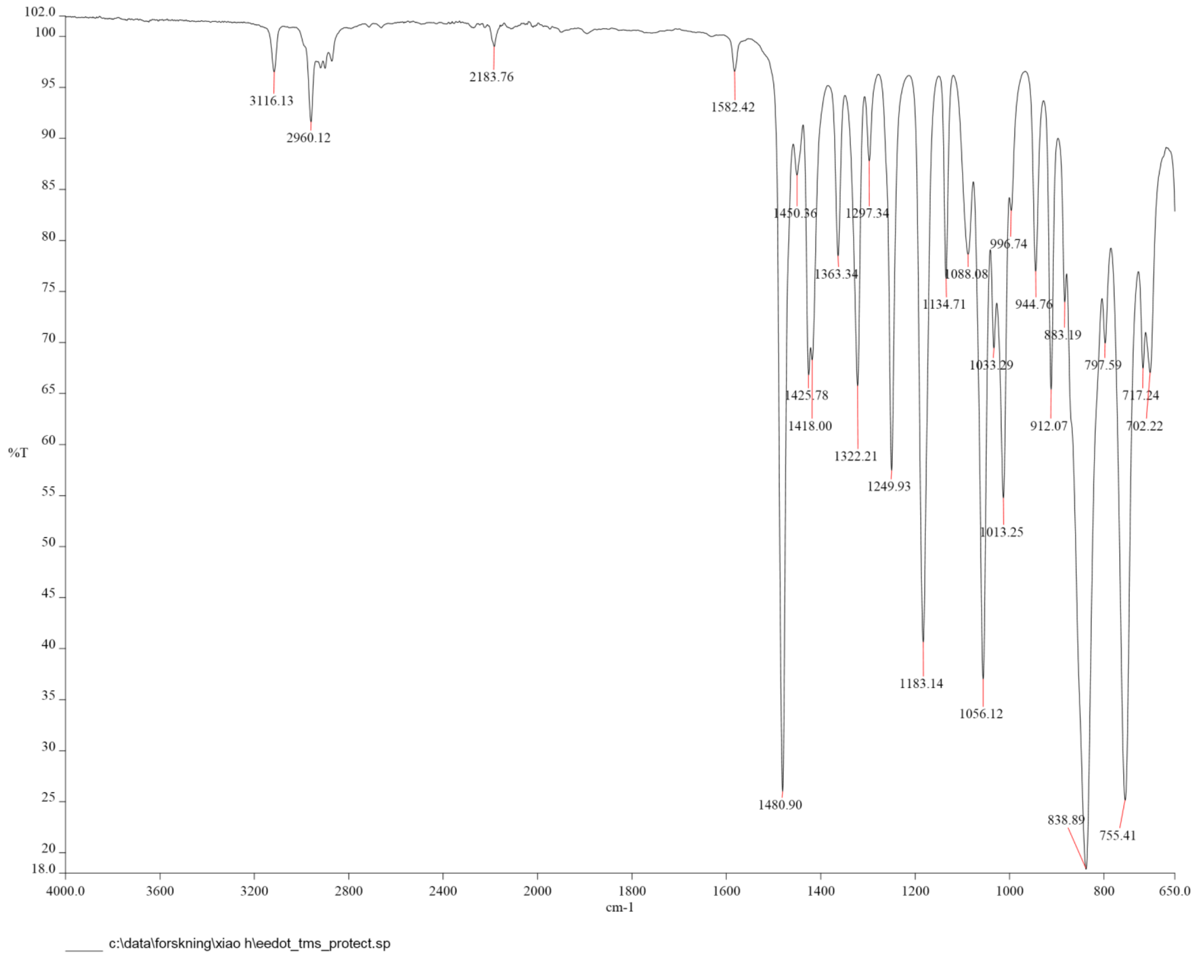

Figure S24: IR spectrum of **8’**.

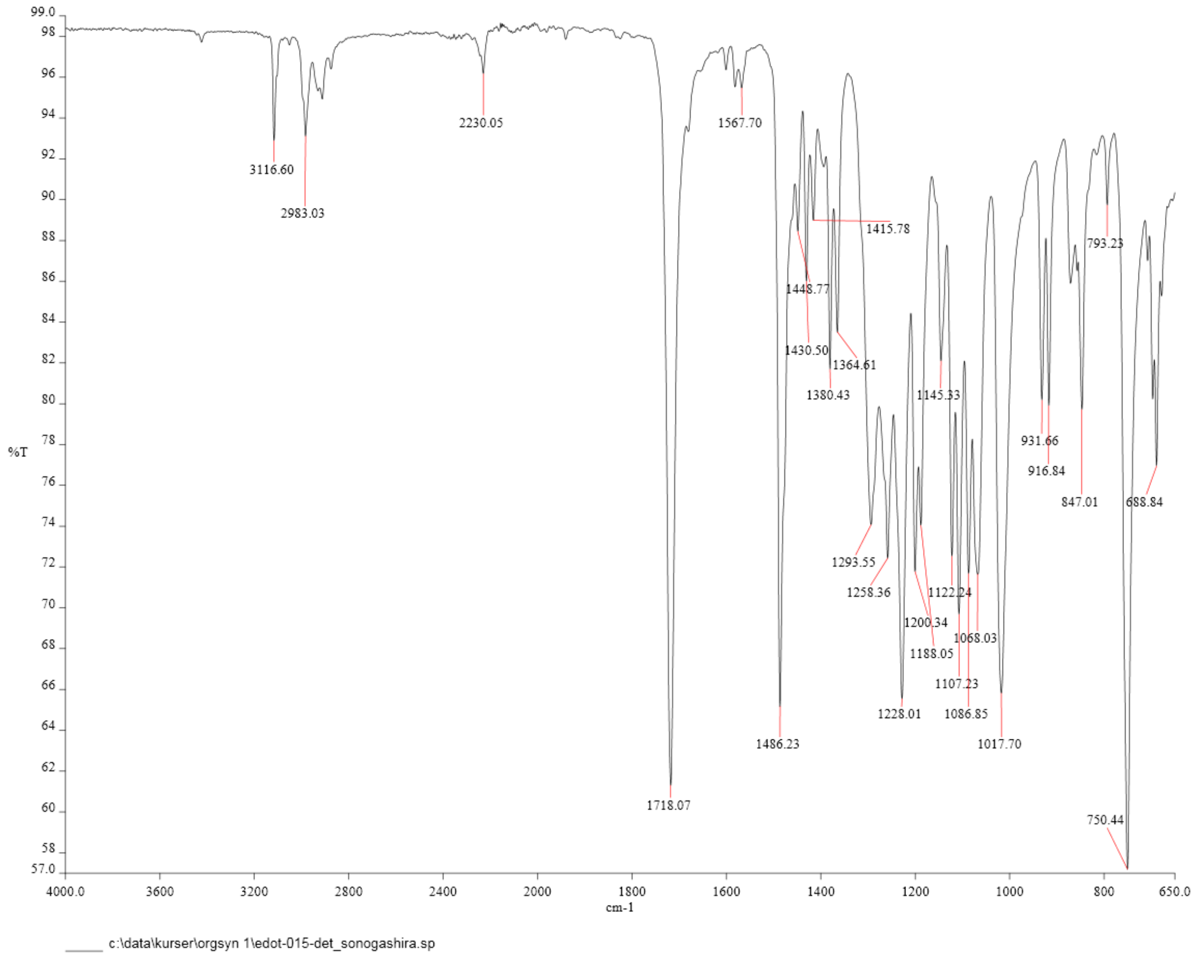

Figure S25: IR spectrum of **9**.

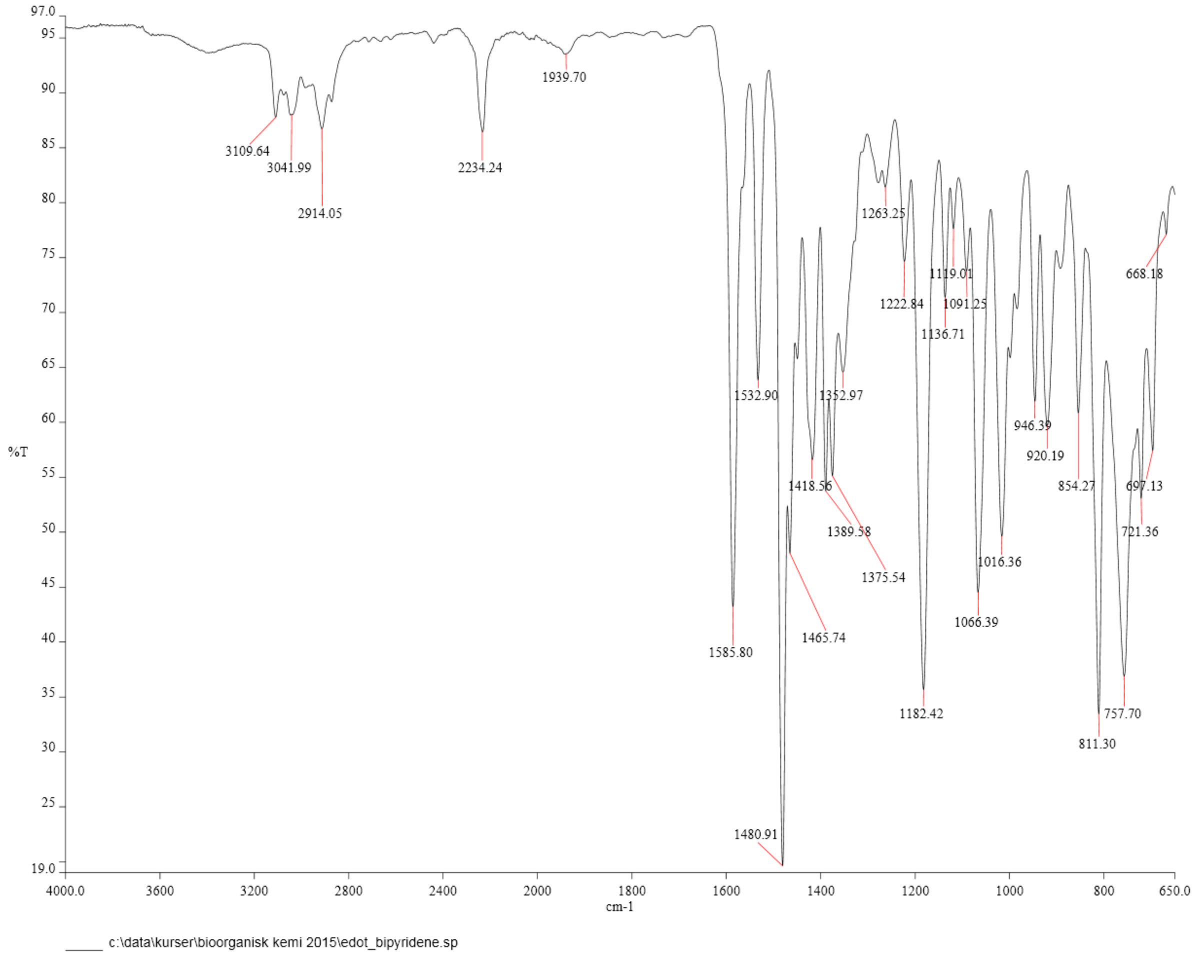

Figure S26: IR spectrum of **10**.

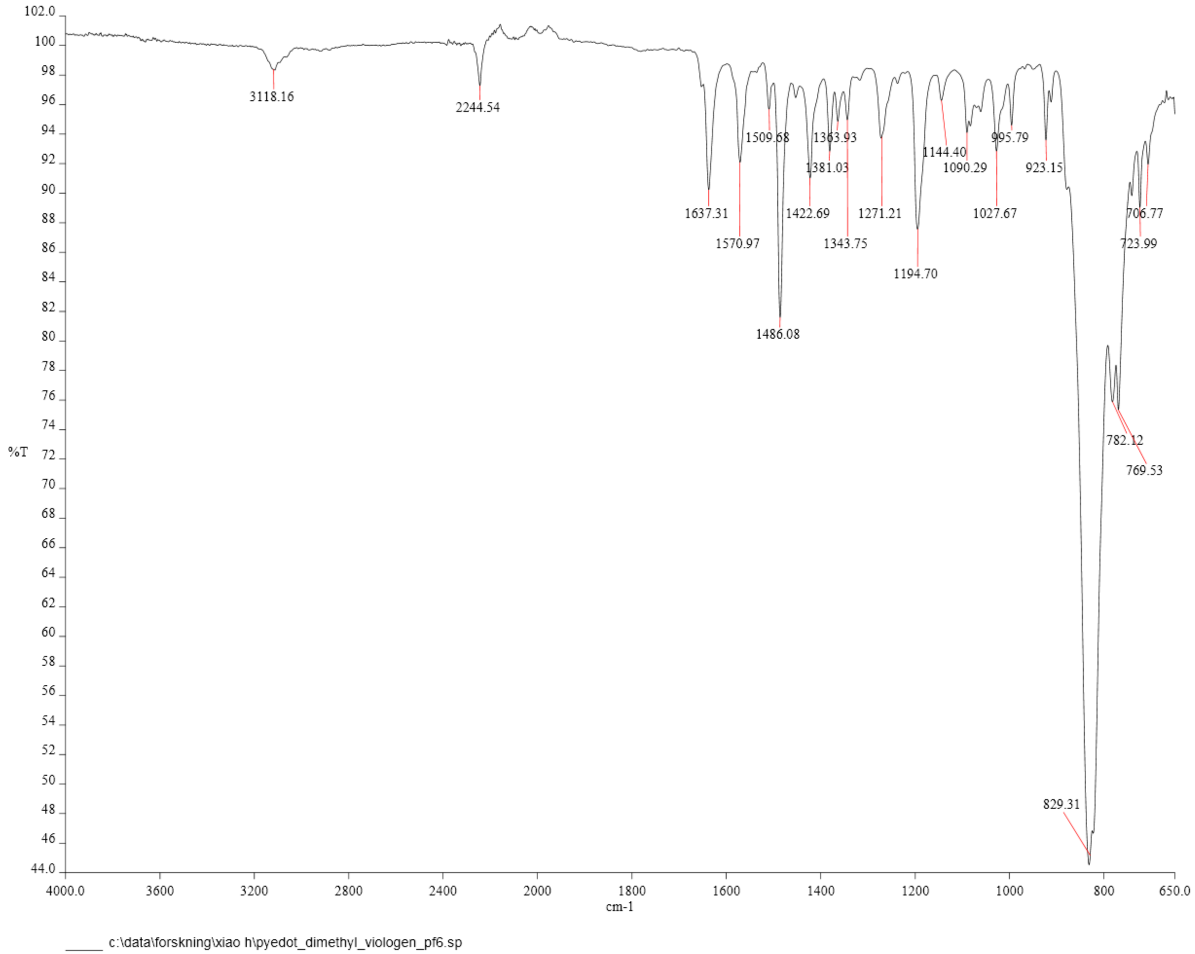

Figure S27: IR spectrum of **11**.

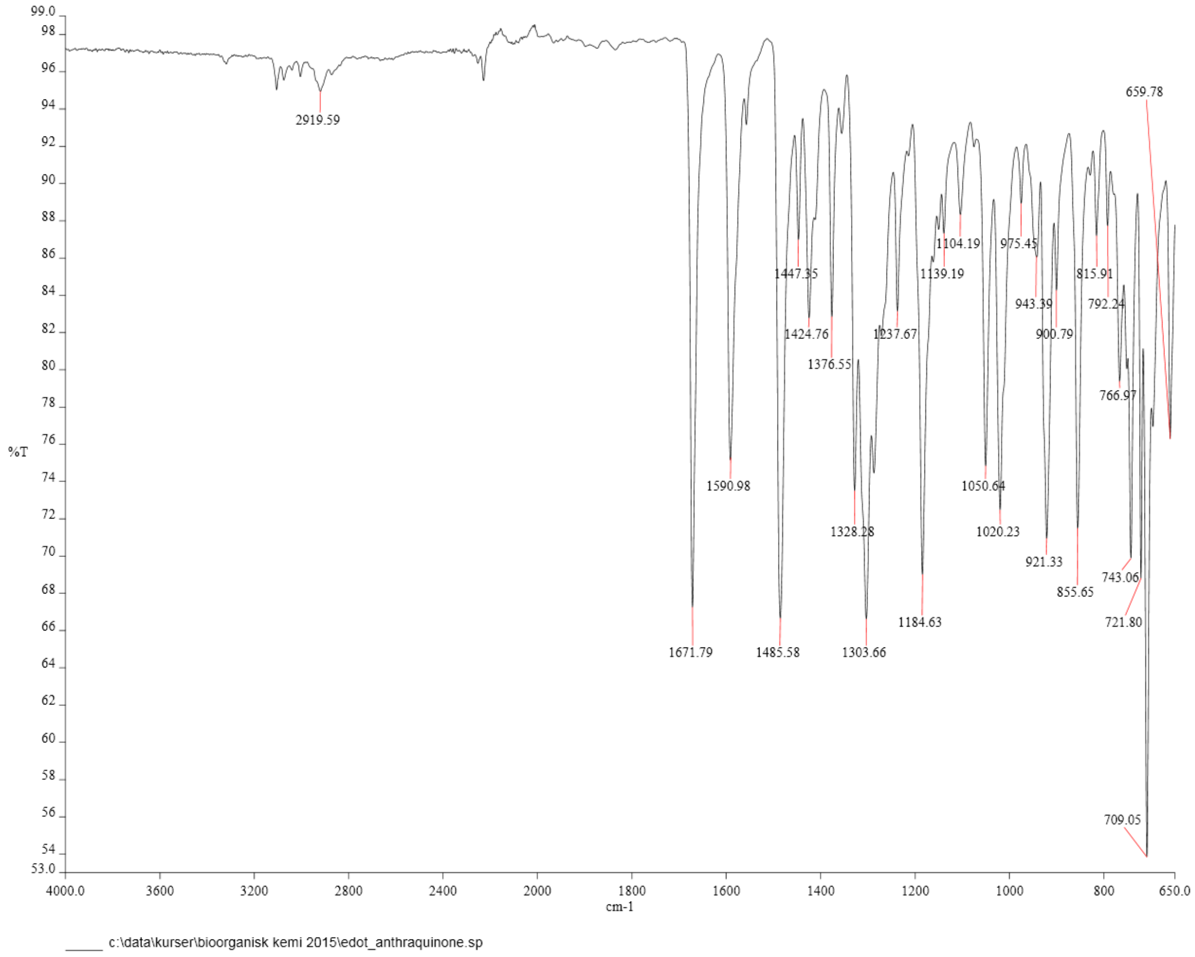

Figure S28: IR spectrum of **12**.

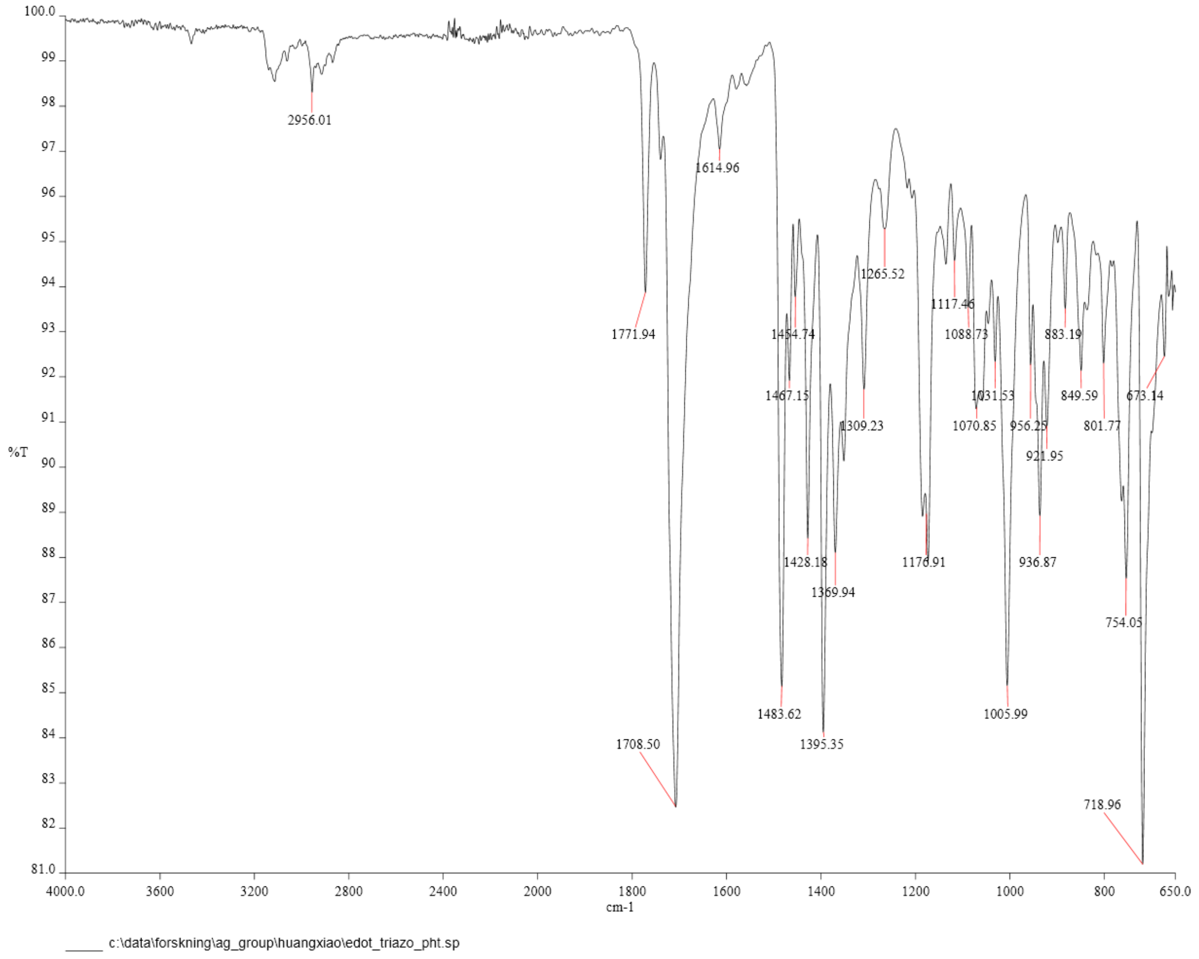

Figure S29: IR spectrum of **13**.

HRMS spectra

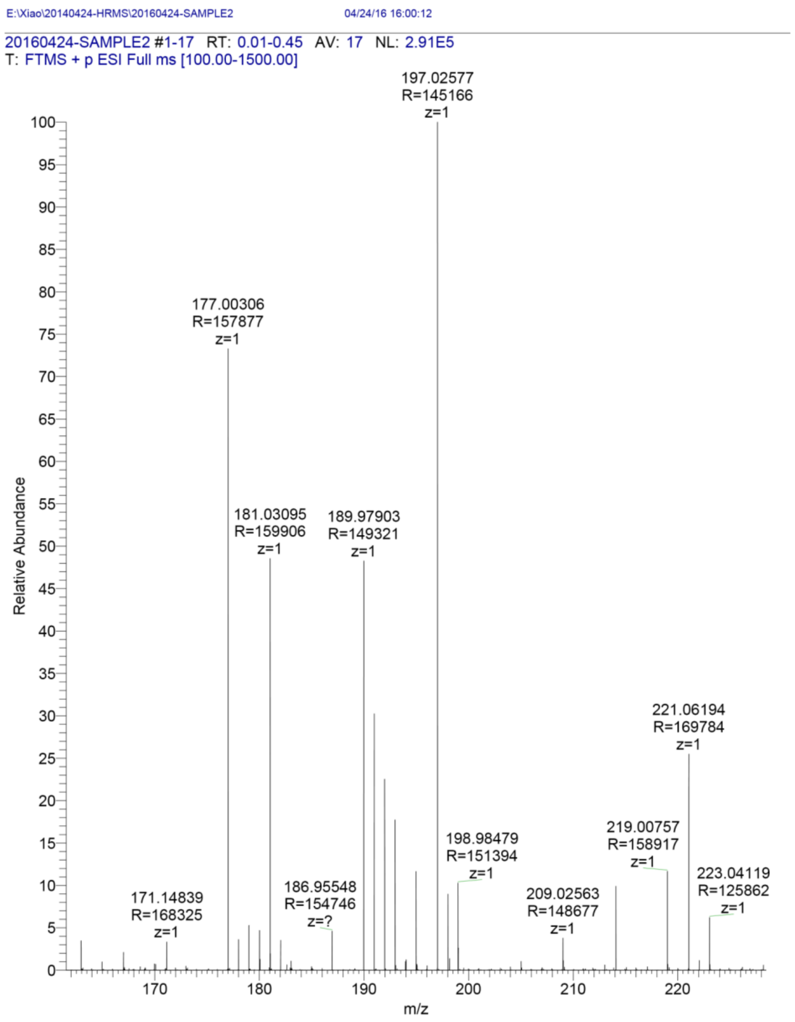

Figure S30: HR-MS of **3**.

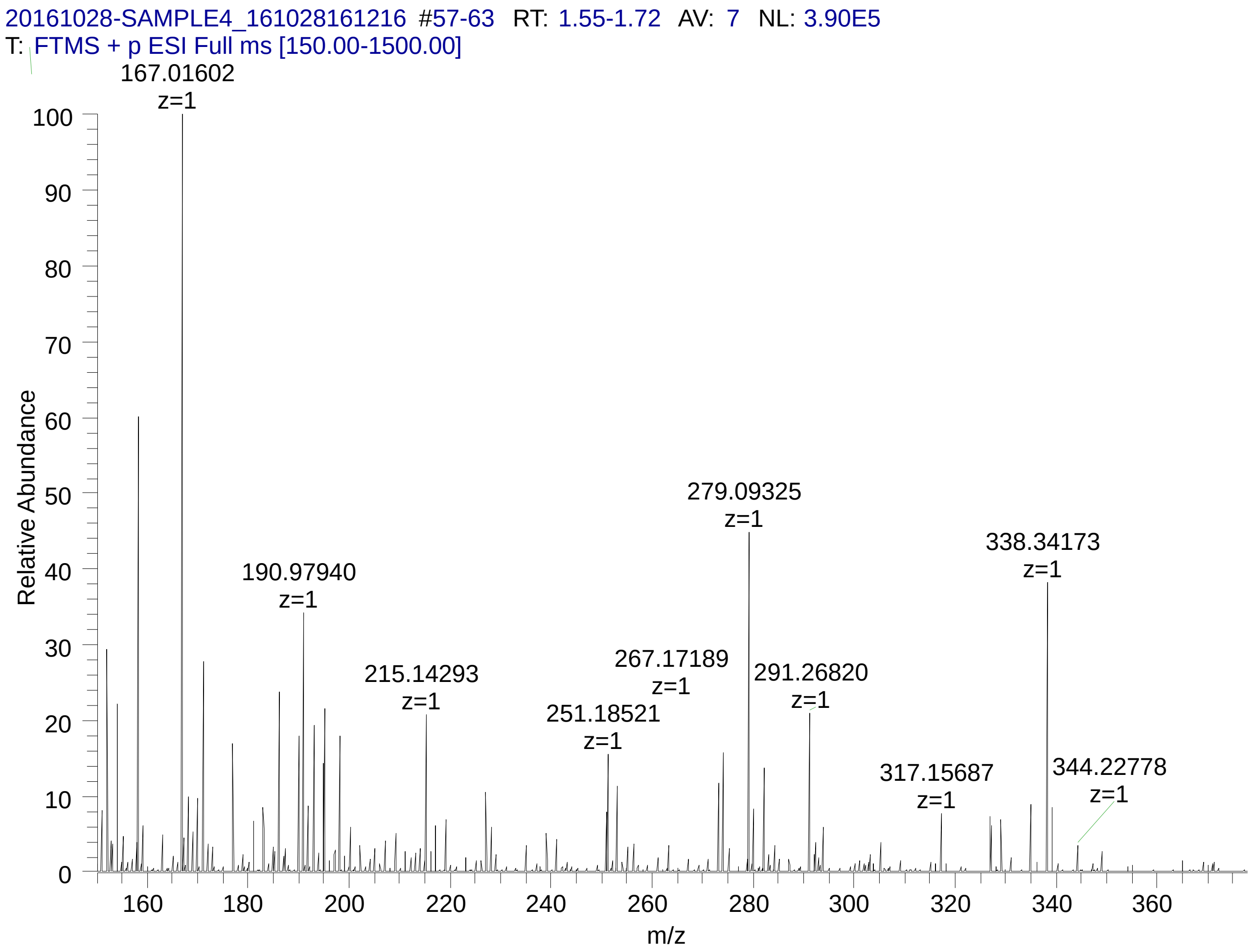

Figure S31: HR-MS of **8**.

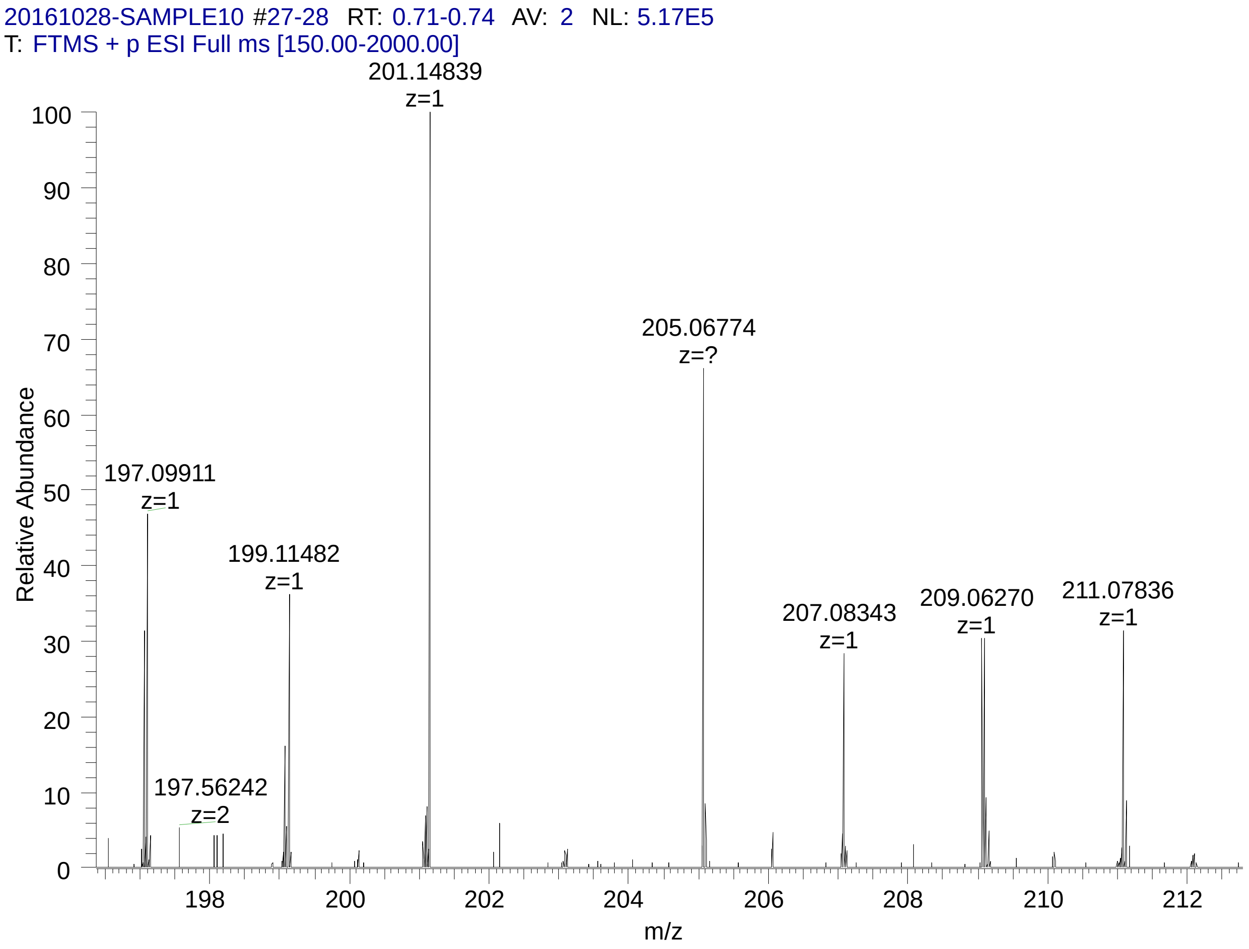

Figure S32: HR-MS of **6'**.

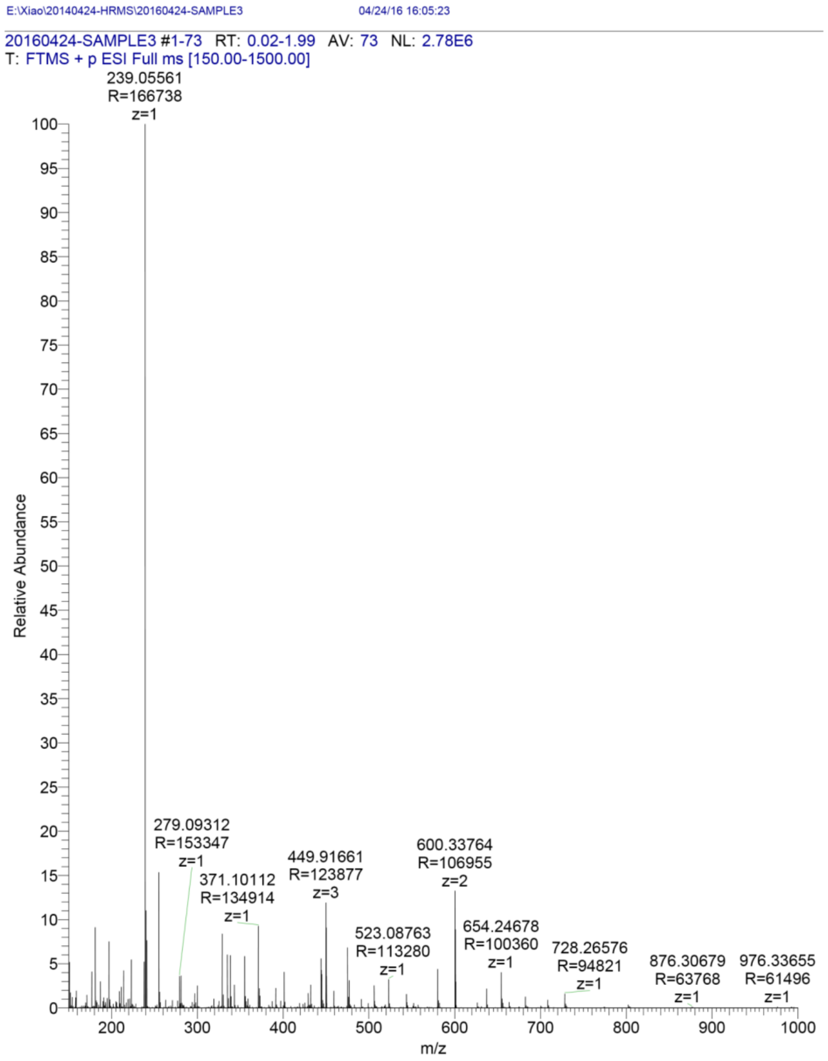

Figure S33: HR-MS of **8’**.

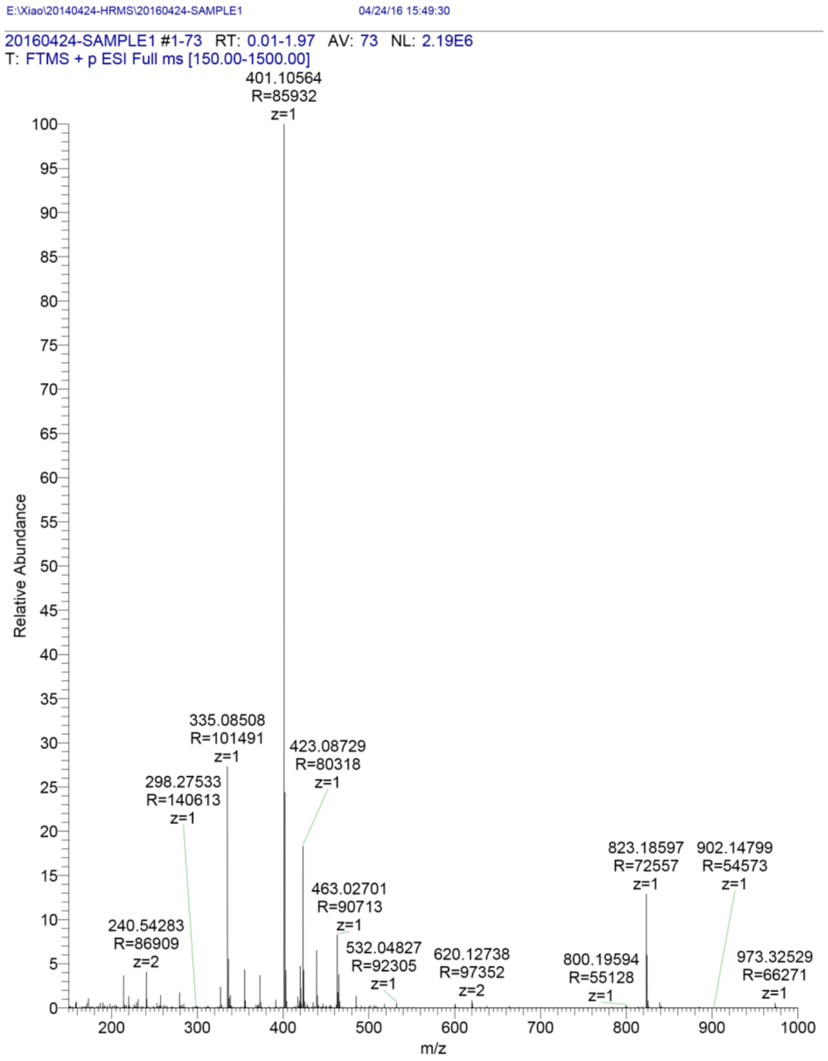

Figure S34: HR-MS of **9**.

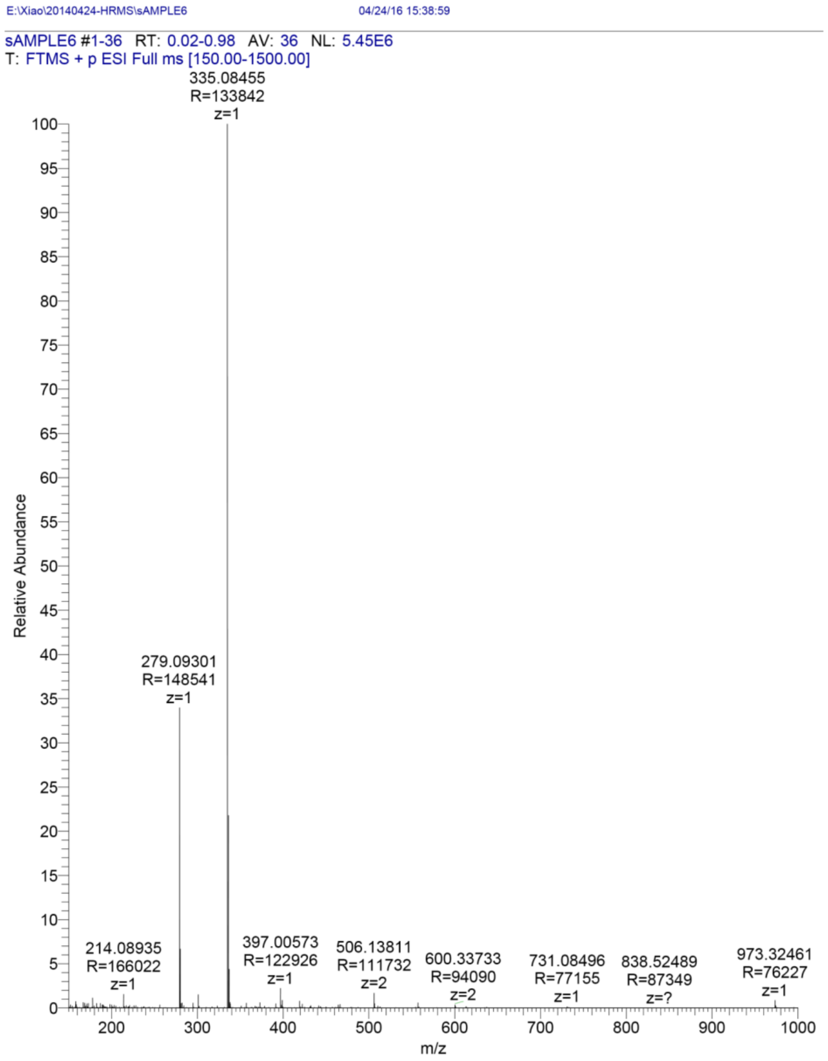

Figure S35: HR-MS of **10**.

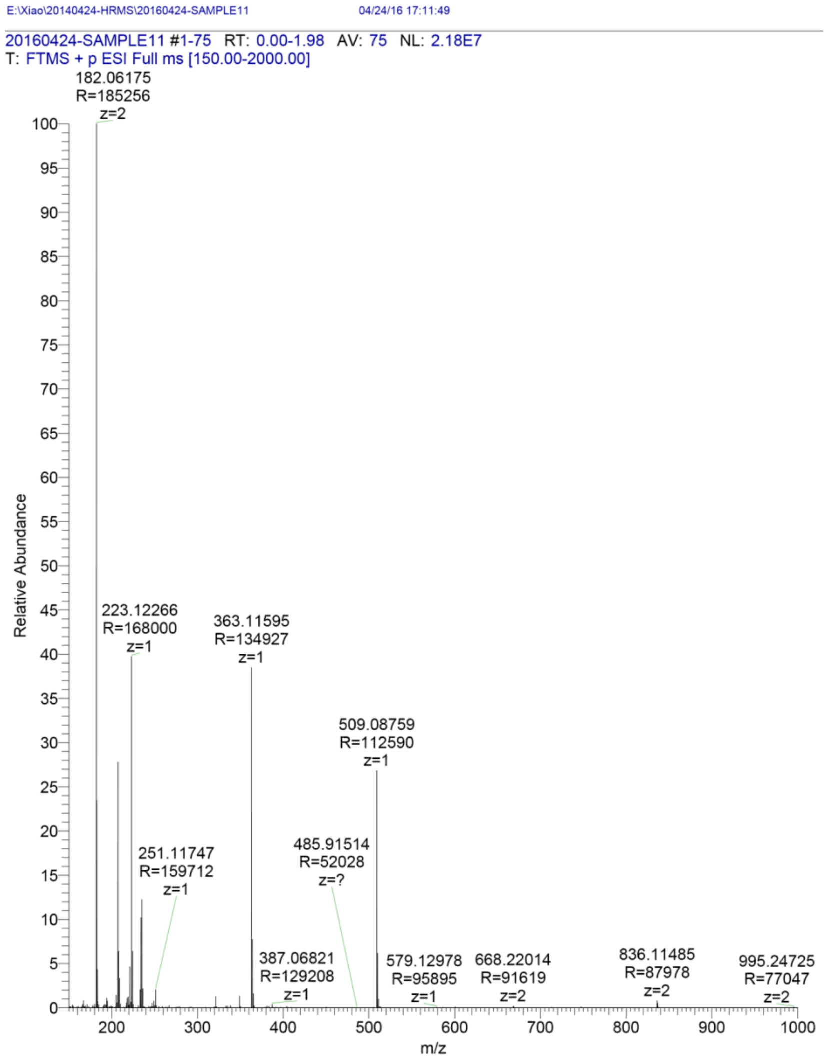

Figure S36: HR-MS of **11**.

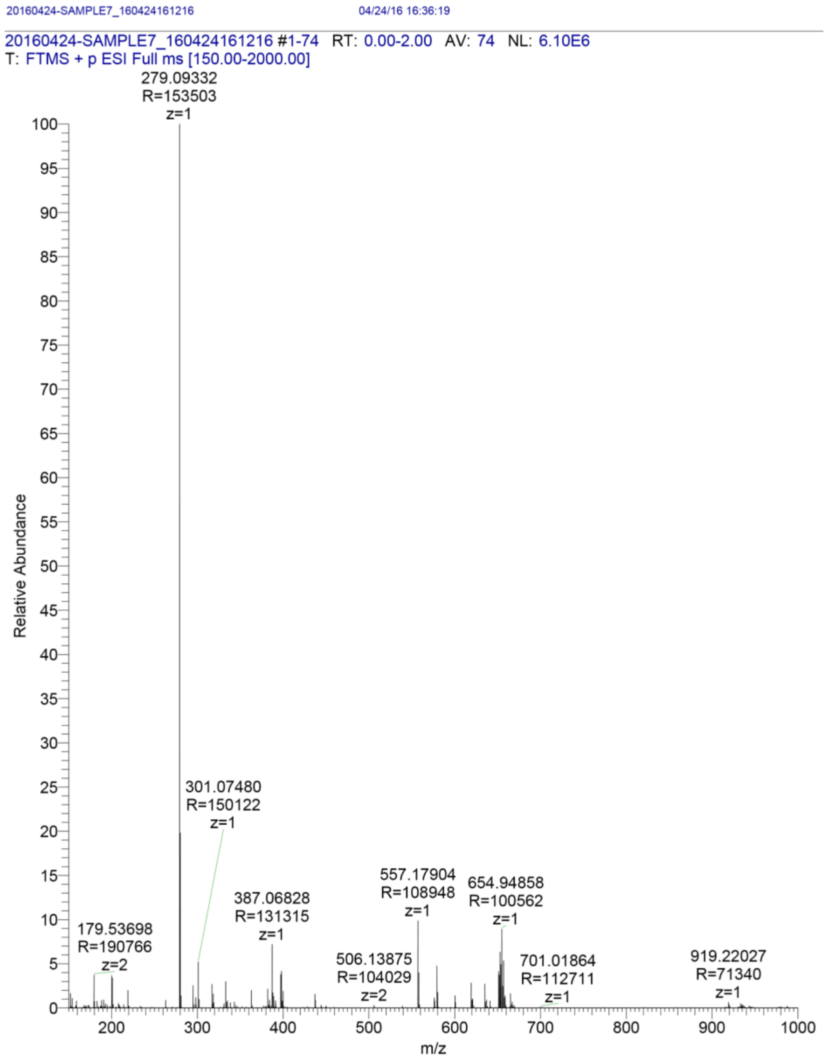

Figure S37: HR-MS of **12**.

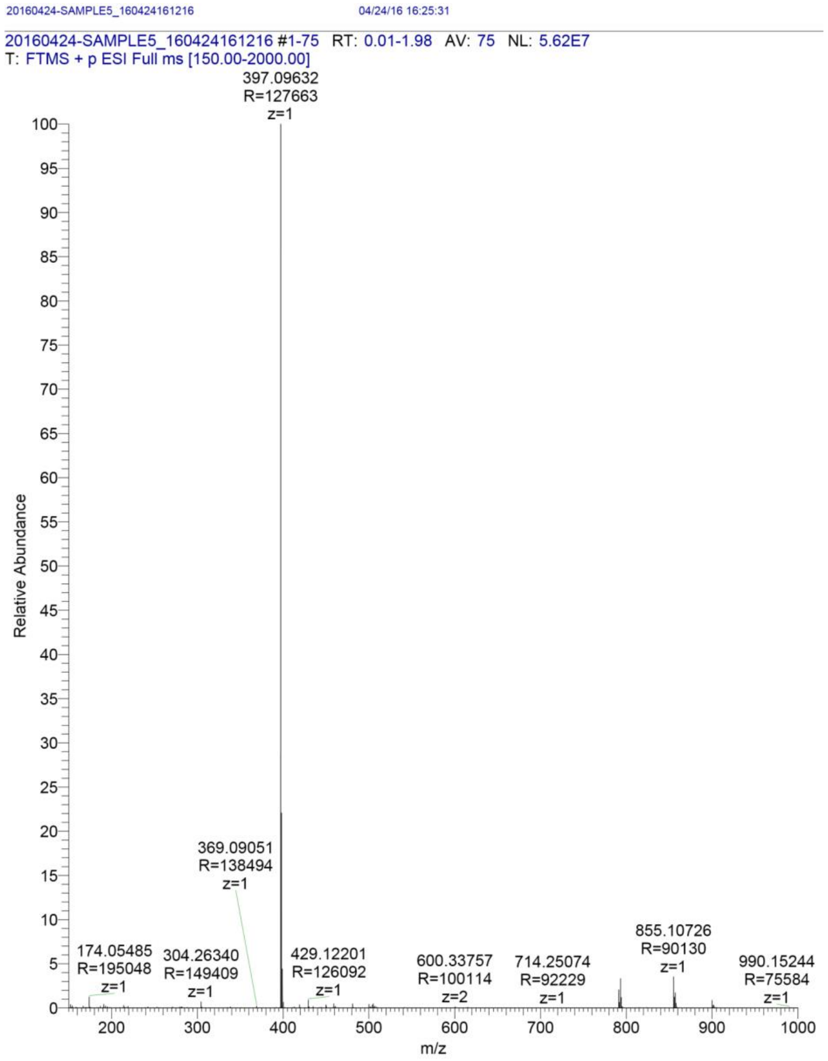

Figure S38: HR-MS of **13**.

Eletrochemistry data

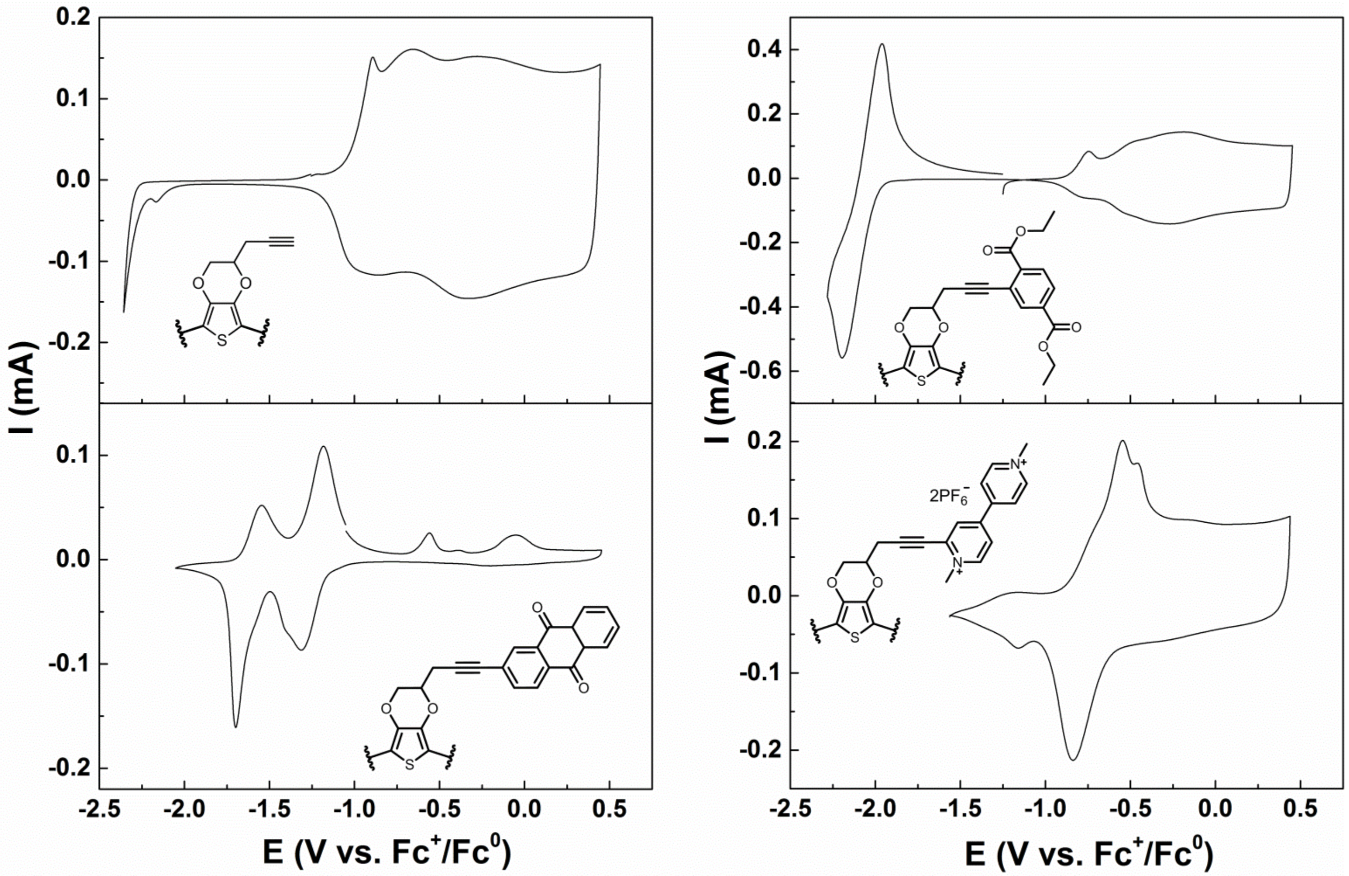

Figure S39: The CVs of the polymers: Poly(**pyEDOT**), Poly(**9**), Poly(**11**), Poly(**12**).

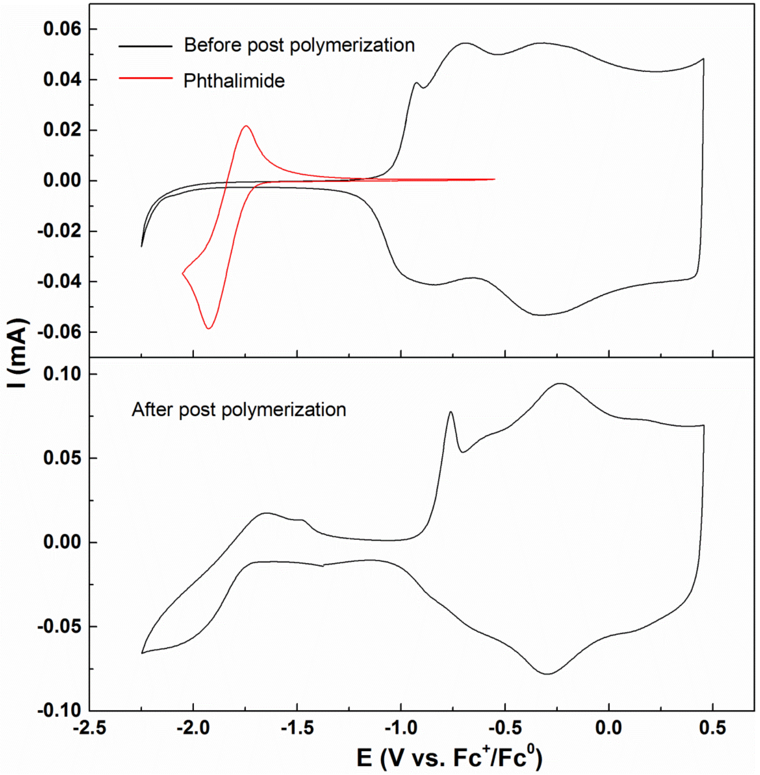

Figure S40: The CVs of post-polymerization functionalization of **pyEDOT** by azide-phthalimide. Top: The black curve shows the backbone redox reaction of poly(**pyEDOT**) before functionalization; the red curve shows the redox reaction of phthalimide. Bottom: Redox behavior of poly(**pyEDOT**) after “click” functionalization with N-(2-azidoethyl)phthalimide.

References

1. Wokaun, A.; Ernst, R. R. *Chem. Phys. Lett.* **1977,** *52*, 407-412.
2. Shaka, A. J.; Freeman, R. *J. Magn. Reson.* **1983,** *51*, 169-173.
3. Mueller, L. *J. Magn. Reson.* **1987,** *72*, 191-196.
4. Davis, A. L.; Keeler, J.; Laue, E. D.; Moskau, D. *J. Magn. Reson.* **1992,** *98*, 207-216.
5. Hurd, R. E.; John, B. K. *J. Magn. Reson.* **1991,** *91*, 648-653.
6. Wagner, R.; Berger, S. *J. Magn. Reson. Ser. A* **1996,** *123*, 119-121.
7. Bax, A.; Davis, D. G. *J. Magn. Reson.* **1985,** *63*, 207-213.
8. Braunschweiler, L.; Ernst, R. R. *J. Magn. Reson.* **1983,** *53*, 521-528.
9. Yang, L.; Huang, X.; Gogoll, A.; Strømme, M.; Sjödin, M. *J. Phys. Chem. C* **2015,** *119*, 18956-18963.
10. Donohoe, T. J.; Ironmonger, A.; Kershaw, N. M. *Angew. Chem. Int. Ed.* **2008,** *47*, 7314-7316.
11. Sugisaki, C. H.; Ruland, Y.; Baltas, M. *Eur. J. Org. Chem.* **2003**, 672-688.
12. Gung, B. W.; Kumi, G. *J. Org. Chem.* **2003,** *68*, 5956-5960.
13. Taylor, E. C.; Ray, P. S.; Darwish, I. S.; Johnson, J. L.; Rajagopalan, K. V. *J. Am. Chem. Soc.* **1989,** *111*, 7664-7665.
14. Gooding, O. W.; Beard, C. C.; Jackson, D. Y.; Wren, D. L.; Cooper, G. F. *J. Org. Chem.* **1991,** *56*, 1083-1088.
15. Sirotkina, J.; Grigorjeva, L.; Jirgensons, A. *Eur. J. Org. Chem.* **2015**, 6900-6908.
16. Huang, X.; Yang, L.; Bergquist, J.; Strømme, M.; Gogoll, A.; Sjödin, M. *J. Phys. Chem. C* **2015,** *119*, 27247-27254.
17. Duan, X. F.; Li, X. H.; Li, F. Y.; Huang, C. H. *Synthesis-Stuttgart* **2004**, 2614-2616.
18. Iordache, A.; Oltean, M.; Milet, A.; Thomas, F.; Baptiste, B.; Saint-Aman, E.; Bucher, C. *J. Am. Chem. Soc.* **2012,** *134*, 2653-2671.
19. Pyta, K.; Klich, K.; Domagalska, J.; Przybylski, P. *Eur. J. Med. Chem.* **2014,** *84*, 651-676.
